# Glycometabolism change during *Burkholderia pseudomallei* infection in RAW264.7 cells by proteomic analysis

**DOI:** 10.1038/s41598-022-16716-z

**Published:** 2022-07-22

**Authors:** Xuexia Li, Yingfei Zeng, Shengnan Guo, Chen Chen, Lin Liu, Qianfeng Xia

**Affiliations:** Key Laboratory of Tropical Translational Medicine of Ministry of Education, NHC Key Laboratory of Tropical Disease Control, School of Tropical Medicine and The Second Affiliated Hospital, Hainan Medical University, Haikou, 571199 Hainan China

**Keywords:** Cell biology, Microbiology

## Abstract

*Burkholderia pseudomallei* is a Gram-negative intracellular bacterium that causes melioidosis, a life-threatening disease. The interaction of *B. pseudomallei* with its host is complicated, and cellular response to *B. pseudomallei* infection is still largely unknown. In this study, we aimed to determine host-cell responses to *B. pseudomallei* at the proteomics level. We performed proteomic profiling of *B. pseudomallei* HNBP001-infected mouse macrophage RAW264.7 cells to characterize the cellular response dynamics during infection. Western blot analysis was utilized for the validation of changes in protein expression. Gene Ontology (GO) and Kyoto Encyclopedia of Genes and Genomes (KEGG) pathway analyses were conducted using the clusterProfiler R package. Compared with the negative control (NC) group, 811 common proteins varied over time, with a cut-off level of two fold change and an adjusted *P*-value less than 0.05. The bioinformatics analysis revealed that the proteins significantly changed in the *B. pseudomallei* HNBP001 infection group (*Bp* group) were enriched in glycometabolism pathways, including glycolysis, fructose and mannose metabolism, pentose phosphate pathway, galactose metabolism, and carbon metabolism. Western blot analysis verified three selected proteins involved in glycometabolism pathways, namely PGM1, PKM, and PGK1 were increase over time post the infection. Furthermore, in vitro functional analysis revealed an increased glucose uptake and decreased ATP production and O-GlcNAcylation in the *Bp* group compared with control group, suggesting that *B. pseudomallei* HNBP001 infection induces changes in glycometabolism in RAW264.7 cells. These results indicate that glycometabolism pathways change in RAW264.7 cells post *B. pseudomallei* HNBP001 infection, providing important insights into the intimate interaction between *B. pseudomallei* and macrophages.

## Introduction

Melioidosis is a fatal infectious disease caused by the Gram-negative bacterium *Burkholderia pseudomallei*. Humans and animals acquired infections as a result of exposing broken skin and inhalation or ingestion of *B. pseudomallei*^1^. In patients with melioidosis, the most common clinical manifestations are acute pneumonia and septicemia, and they are associated with a high mortality rate (10–30%)^2^. Based on the increase in the number of reported cases and the expansion of the endemic area over the past decades, melioidosis is considered as a re-emerging infectious disease in many tropical countries^3^. It is commonly reported in Africa, South America, Northern Australia and Asia^4,5^. In China, melioidosis is primarily reported in the southeast coastal regions, including Hainan, Guangdong, Fujian, and Taiwan^6,7^. In recent years, there has been a rise in the number of melioidosis cases witnessed in this region, particularly in Hainan.

Between 2002 and 2016, a total of 392 culture-confirmed melioidosis cases were identified in Hainan. Furthermore, among them, 289 cases have the available medical records, and were reviewed to establish information about the demographics, clinical features, and outcomes^8^. In the early study, we isolated the *B. pseudomallei* strain HNBP001 from the blood of a melioidosis patient with pneumonia in Hainan and reported the genome sequence of the HNBP001^9^. Thus, it is a significant public health concern when many individuals are at risk of being infected by *B pseudomallei*^10^.

Most infection mechanisms involve alterations in protein expression; proteins are essential for cell growth, proliferation, and survival^11,12^. Identifying the differentially expressed proteins of host cells and examining their interrelationships, such as enriched biological processes and pathways, will provide insight into the combined influence of *B. pseudomallei* on body function. There have been some studies explored the interaction between hosts and *B. pseudomalle i*^13–15^. For example, Loaiza et al. predicted of host- pathogen protein interactions in *B. pseudomallei* and human in silico, discovered the host targets of the pathogen proteins and proteins that form T3SS and T6SS in *B. pseudomallei.* Schmidt et al. found that *B. pseudomallei* modulated host iron homeostasis to facilitate intracellular survival.

Given that RAW264.7 cells have been widely used for modeling *B. pseudomallei* in vitro^16–18^, in this study we aimed to establish the *B. pseudomallei* HNBP001 infection model of RAW264.7 cells (*Bp*) and utilize this model to investigate the host-cell responses. For this purpose, we performed high-resolution liquid chromatography-tandem MS (LC–MS/MS) in the *Bp* group and negative control (NC) group^19^. Our results demonstrate that a large number of proteins in host cells are altered significantly after infection with *B. pseudomallei* HNBP001, which might provide a new direction for future work to reveal the pathogenic mechanism of *B. pseudomallei* in macrophages.

## Results

### RAW264.7 cell *B. pseudomallei* HNBP001 infection model

**Figure 1:**
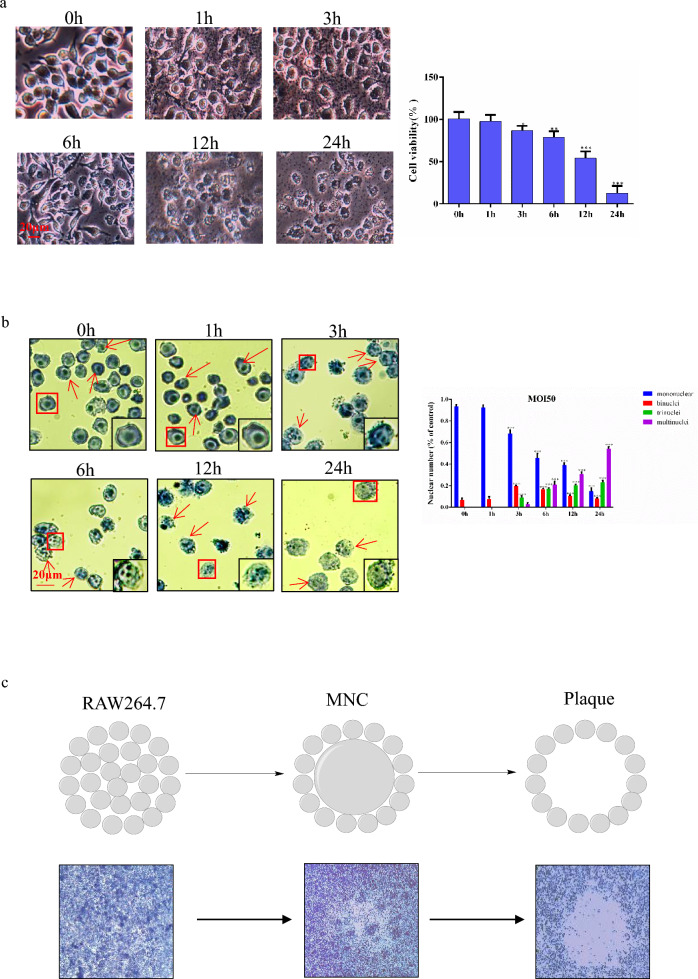

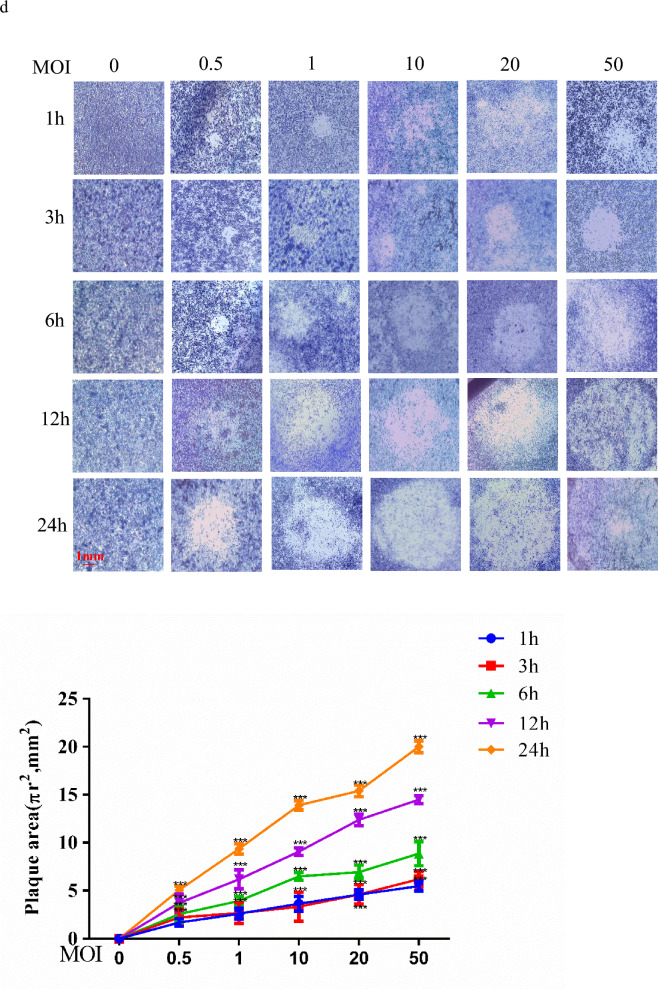
RAW264.7 cell B. pseudomallei HNBP001 infection model. (**A**) RAW264.7 cells were infected with B. pseudomallei HNBP001 MOI 50 at 0, 1, 3, 6, 12, or 24 h; cell morphology was observed under the microscope; and quantification of cell viability was indicated. (**B**) RAW264.7 cells were stained with Giemsa, and MNGCs were counted. Quantification of MNGCs was indicated. Mononuclear means the number of cells only with one nuclear, binucleus means the number of cells with two nuclei, trinucleus means the number of cells with three nucleus, and the multinuclus means the number of cells with more than three nucleus. (**C**) Cell fusion assay with example well images show RAW264.7 cell monolayers infected with B. pseudomallei HNBP001 and stained with Giemsa. (**D**) The relative abundance and size of plaques while infected with B. pseudomallei HNBP001 was assessed at different MOI or times. We conducted the same experiment three times independently.

To establish an in vitro* B. pseudomallei* HNBP001 infection model, we infected RAW264.7 cells with *B. pseudomallei* HNBP001 at different MOI or time-points following the protocol described in a previous study^20^. We determined the cell morphology under a microscope and noted cell viability decreasing with the duration of infection (Fig. 1A). As shown in Fig. 1B, at 6 h post-infection, *B. pseudomallei* HNBP001 induced 50% MNGC (multinuclear giant cell) formation, with 80% MNGC formation at 12 h post-infection, and 95% MNGC formation at 24 h post-infection, where almost all the cells died at this time. We adapted the cell fusion assay shown in Fig. 1C for screening by seeding RAW264.7 cells into a 10 cm dish, infecting cells with *B. pseudomallei* HNBP001, then staining them with Giemsa and imaging them using a microscope to assess the relative abundance and size of plaques (Fig. 1D). According to the results, the RAW264.7 cell *B. pseudomallei* infection model was established successfully.

When infected with *B. pseudomallei* HNBP001 MOI 50 for 12 h, the cell viability decreased 40%, MNGCs increased nearly 80%, and the plaque area increased 2.5 fold, suggesting that the proteins in RAW264.7 cells have changed. Thus RAW264.7 cells infected with *B. pseudomallei* HNBP001 MOI 50 for 12 h (*Bp* group), and matching RAW264.7 cells (NC group), were subjected to proteome profiling analysis.

### Proteome profiling of *B. pseudomallei* HNBP001 infected RAW264.7 cells

To determine the proteome profiling, we employed a LC–MS/MS for proteome profiling and digested extracted proteins from cells with trypsin. The resulting peptides were labeled with TMT/iTRAQ and analysed on QE Plus mass spectrometer. The LC–MS/MS platform was stable and repeatable, as judged by quality control runs during the overall data-collecting period (Supplementary Fig. 1).

**Figure 2:**
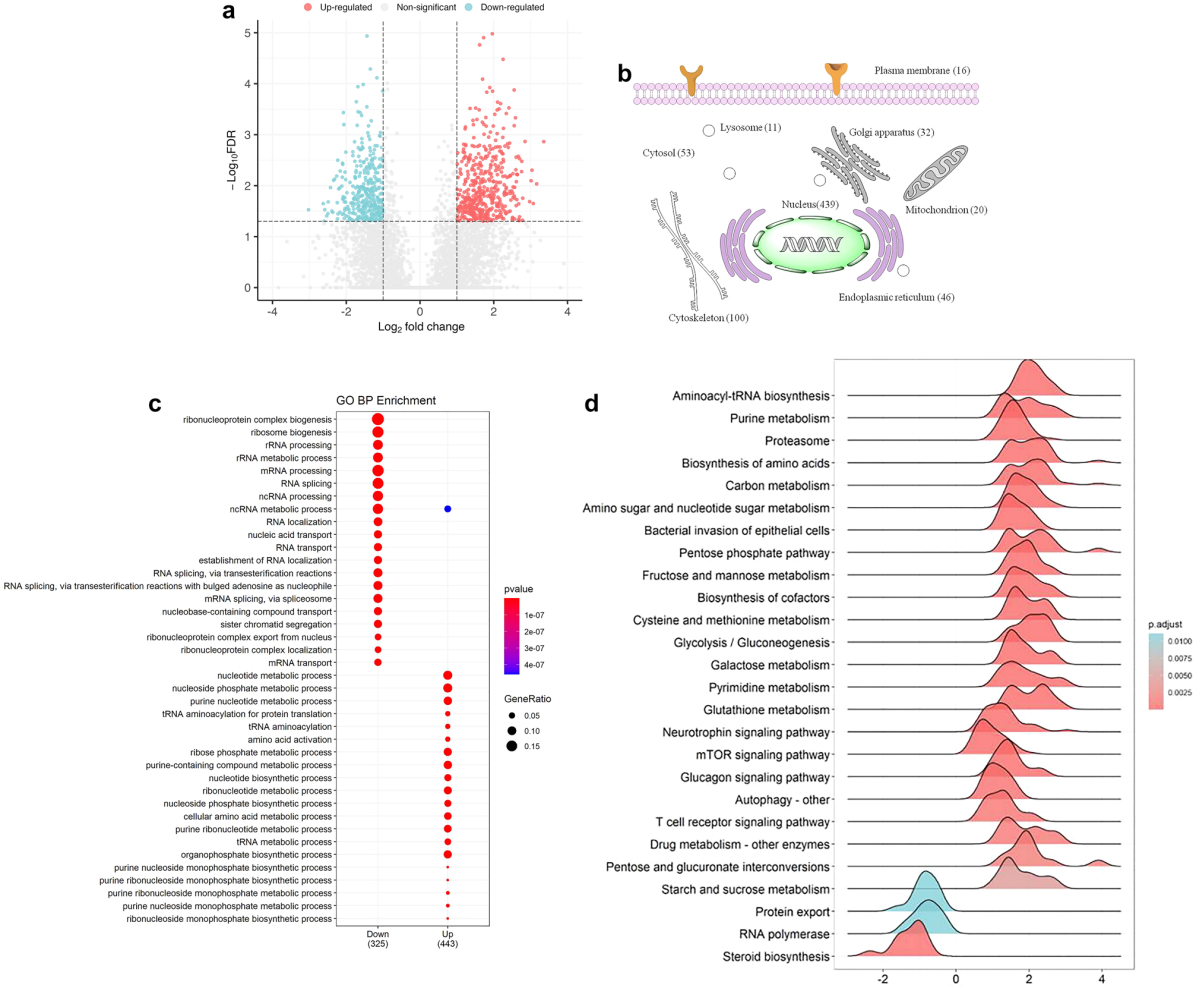
Proteome profiling of B. pseudomallei HNBP001 infected RAW264.7 cells. (**A**) Volcano plot of 811 differential expressed proteins. The Log2-transformed fold change and − log 10-transformed adjusted *P*-value were denoted by the x-axis and y-axis, respectively. (**B**) Subcellular distribution of proteins was annotated with Gene Ontology. Many proteins were annotated as the intracellular matrix, and 50% of proteins were located in the nucleus. (**C**) The Biological Processes altered in the Bp group compared with the NC group. The top 20 significantly altered terms of up-regulated and down-regulated proteins were depicted in the picture. (**D**) The ridgeline plot for expression distribution of core-enriched proteins in KEGG categories, where the x-axis represented Log2-transformed fold change.

We analyzed the raw files together for uniformed quality control, and adjusted protein identification with *P*-values using the Holm method^21^. In total, we identified a total of 6671 proteins and quantified 5924 proteins by the LC–MS/MS. A SAM (significance analysis of microarray)^22^ analysis identified 811 proteins as differentially expressed between *Bp* group and NC group, with statistical significance (Holm adjust *P* < 0.05, Fold Change > 2 or < 0.5) (Fig. 2A, Supplementary Table 1). As shown in Fig. 2B, many proteins were annotated as the intracellular matrix, and 50% of proteins were located in the nucleus, suggesting that our proteome dataset includes the cellular metabolism.

The Gene Ontology annotation indicated that the up-regulated proteins were significantly enriched in ribonucleoprotein complex biogenesis, ribosome biogenesis, rRNA processing, and mRNA processing (Fig. 2C, Supplementary Table 2). The KEGG GSEA enrichment analysis showed that purine metabolism, carbon metabolism, pentose phosphate pathway, fructose and mannose metabolism, and glycolysis pathways were significantly enriched (Fig. 2D, Supplementary Table 3).

### Change of proteins involved in glycometabolism pathways

We next characterized those differentially expressed proteins which are involved in glycommetabolism pathways, including those involved in glycolysis (-LDHA, PGM2, PKM, PGM1-), carbon metabolism (-ADH5, GOT1, PGK1, G6PDX-), pentose phosphate pathway (-ALDOA, PGLS, TALDO1-), fructose and mannose metabolism (-PGLS, TALDO1-), and galactose metabolism (-AKR1B7, AKR1B1, UGP2, GALK1-). As summarized in Table 1, all those proteins were up-regulated in the *Bp* group, suggesting that the glycometabolism of host cells changed while being infected with *B. pseudomallei* HNBP001.

**Table 1:** Chnage of proteins involved in glycometabolism pathways.

Gene name	Express ratio (*Bp* group/NC group)	*P* value	FDR	Involved in signaling pathway
LDHA	5.385	2.64E−07	0.001462986	glycolysis
PGM2	5.377	1.55E−09	8.71E−06	
PKM	3.923	4.17E−06	0.021022669	
PGM1	6.486	5.40E−07	0.002948511	
ADH5	3.084	3.17E−06	0.016275224	carbon metabolism
GOT1	4.225	8.19E−07	0.004426946	
PGK1	4.935	3.02E−07	0.001669392	
G6PDX	4.816	9.46E−06	0.044698985	
ALDOA	4.151	9.39E−07	0.005056988	pentose phosphate pathway
PGLS	3.153	3.87E−06	0.019611519	
TALDO1	5.062	6.88E−08	0.000383949	
PGLS	3.153	3.87E−06	0.019611519	fructose and mannose metabolism
TALDO1	5.062	6.88E−08	0.000383949	
AKR1B7	4.035	5.08E−07	0.002780961	galactose metabolism
AKR1B1	6.11	1.58E−06	0.008361881	
UGP2	3.782	7.14E−07	0.003872956	
GALK1	3.06	2.92E−07	0.001615636	

**Figure 3:**
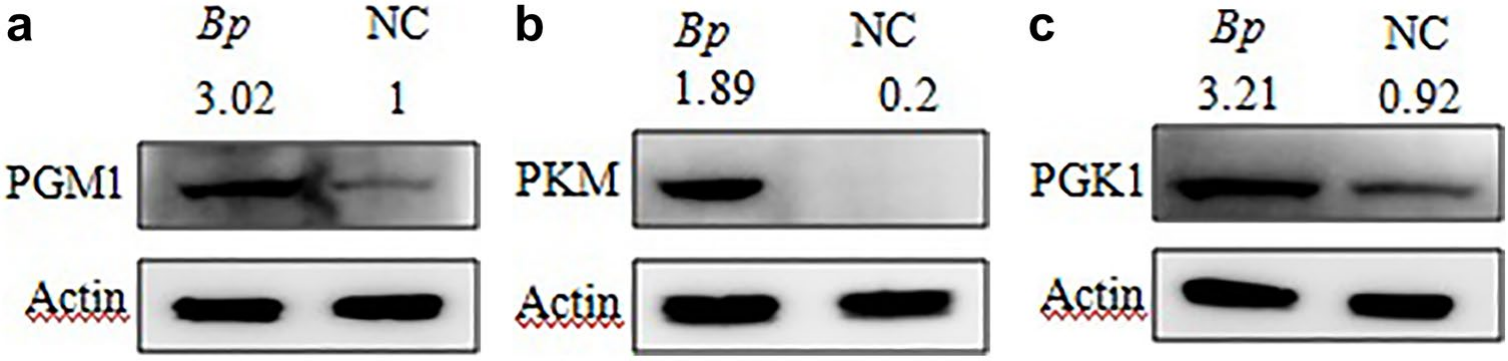
Changes in the expression of enzymes related to glucose metabolism on the Western Blot. (**A**) PGM1 increased 3.02-fold in *BP* group. (**B**) PKM increased 9.45-fold in *BP* group. (**C**) PGK1 increase 3.50-fold in *BP* group.

To validate the proteomic changes, we selected three enzymes in the glycolysis and carbon metabolism pathway—PGM1, PKM, and PGK1—for verification using the Western Blot. As expected, the expression levels of PGM1, PKM, and PGK1 in the *Bp* group were increased about 3.02-fold, 9.45-fold, and 3.5-fold respectively, compared to the NC group (Fig. 3A,B,C).

### Correlation of *B. pseudomallei* HNBP001 model proteomes with glycometabolism

**Figure 4:**
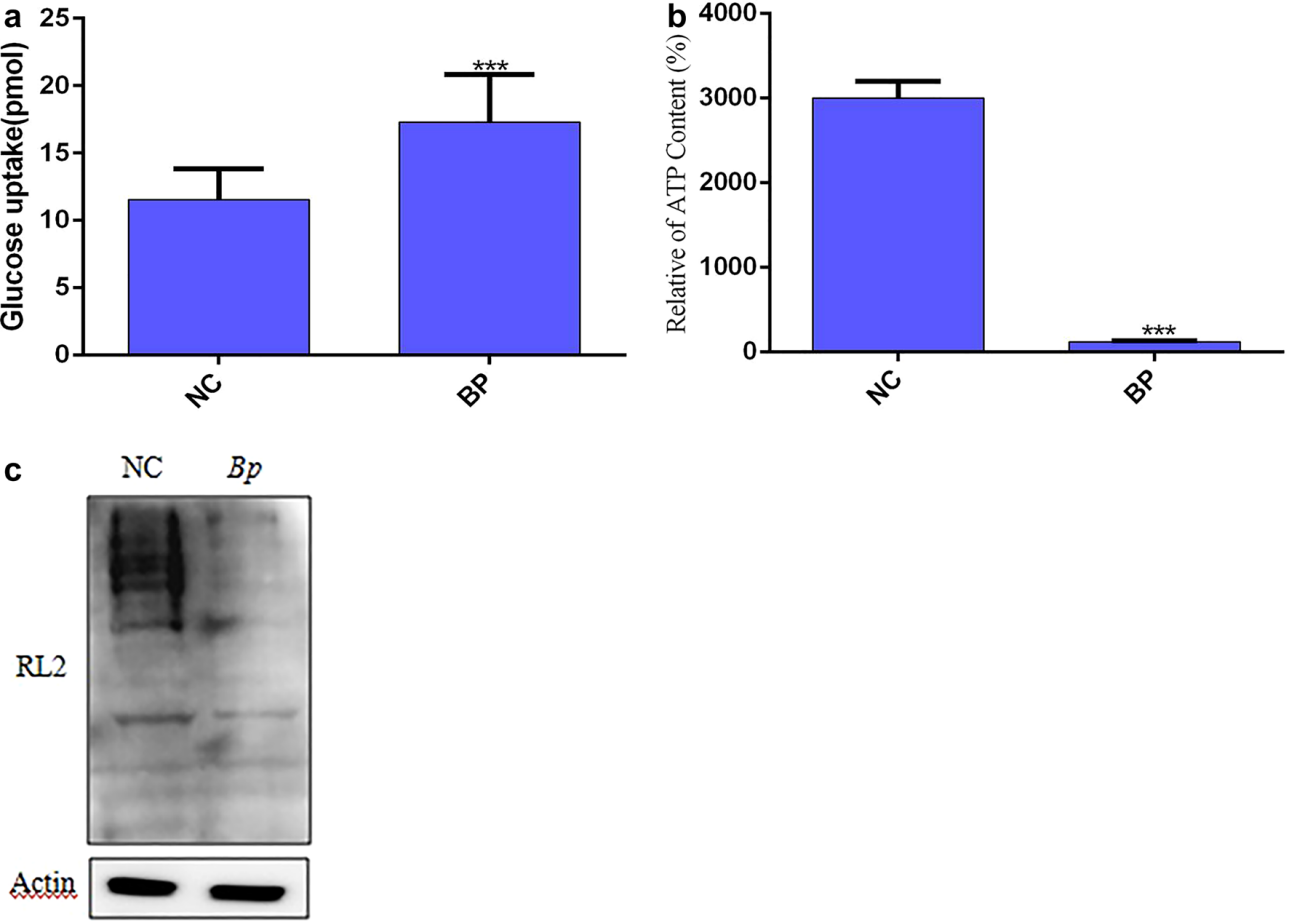
Correlation of *B. pseudomallei* HNBP001 model proteomes with glycometabolism. (**A**) Glucose uptake was significantly altered in the *Bp* group compared with the NC group. The assay was performed according to the Glucose Uptake Assay Kit (Abcam, ab136955). (**B**) The production of ATP was significantly decreased in the *Bp* group compared with the NC group. (**C**) O-GlcNAcylation (RL2) was down-regulated in the *BP* group compared with the NC group. We conducted the same experiment three times independently.

The elevated expression of PGM1, PKM, and PGK1 accelerate the glycolysis pathway^23–25^; thus, we speculated that the *B. pseudomallei* HNBP001 infection accelerated glucose uptake in host cells. As expected, the *Bp* group takes more glucose compared with the NC group (Fig. 4A). We then detected ATP production in the NC and *BP* group, and the production of ATP was significantly reduced in the *Bp* group (Fig. 4B). Glucose also is a substrate of O-GlcNAcylation, a post-translation modification. Therefore, we further examined the level of O-GlcNAcylation in the NC and *Bp* groups. The results showed that the level of O-GlcNAcylation was significantly decreased in the *Bp* group (Fig. 4C), suggesting that *B. pseudomallei* HNBP001 strictly regulates the glucose metabolism of host cells.

## Discussion

Melioidosis is an infectious disease caused by the Gram-negative bacteria *B. pseudomallei*. Elucidating the cellular response to *B. pseudomallei* infection will aid in unraveling the pathogenic mechanisms of the pathogen, and most cellular responses to pathogens involve alterations in protein expression. Thus, we explored host-cell responses to *B. pseudomallei* at the proteomics level in this study. In an earlier study^26^, *B. pseudomallei* infection induced hosts’ membrane fusion, and multiple cell fusion events resulted in the formation of large multinucleate cells, which eventually lyse to form lesions on cell monolayers. It was consistent with the RAW264.7 cell *B. pseudomallei* HNBP001 infection model established by us, and this model is subjected to proteomic analysis successfully.

LC–MS/MS was performed in our study, and we identified 811 proteins when the *P*-values were adjusted using the Holm method. Additionally, 275 proteins increased more than three-fold and 123 proteins decreased more than three-fold in *B. pseudomallei* HNBP001 infected RAW264.7 cells. The bioinformatics analysis showed that many proteins involved in glycometabolism are changed when infected with *B. pseudomallei* HNBP001, which contain proteins involved in glycolysis, carbon metabolism, galactose metabolism, pentose phosphate pathway, and the fructose and mannose metabolism pathway. In early study, Welkos et al.^27^ have identified 160 *B. pseudomallei*’s proteins in the infected RAW 264.7 cells, and 274 host proteins that were exclusively present or absent in infected cells. However, they focused on *B. pseudomallei*’s proteins, constructed mutation genes and assessed their relative virulence. There is a lack of information on the detailed functions of host proteins and relative signaling pathways in their study. Similarly, Mariappan et al.^20^ have identified 44 up regulated and 43 down regulated proteins in A549 cells infected with *Bp*_*MARAN*,_ which related to metabolism, stress response, virulence, signal transduction, or adhesion. Their study targeted to lung cells, however, *B. pseudomallei* usually attacks the host’s immune system first, then causing lesions in organs^4^. Thus, it is more relevant to explore the changes of proteins in macrophages, which can contribute to the study of the infection mechanism of *B. pseudomallei.* To our knowledge, the present study for the first time shows that melioidosis is associated with glycometabolis in immune cells. Additionally, our results provided a platform for the protein expression library of RAW264.7 cells infected with *B. pseudomallei* HNBP001, and deciphering the alterations of proteomic profiling provides insight into the response mechanism of hosts.

Alterations in the expression of proteins involved in glycometabolism can lead to glycometabolism abnormally, as well as diseases. For example, high PGK1 expression resulted in the promotion of glycolysis, enhancing the stem cell-like properties and EMT by activating AKT signaling in oral squamous cell carcinoma cells^28^. High expression of ALDOA is also related to galactose metabolism and colorectal cancer, lung cancer, and gastric cancer^29–31^. Additionally, PKM activation increases glucose metabolic flux, inhibiting the production of toxic glucose metabolites in diabetics^32^. These changes increase glucose uptake because most substrates of glycometabolism are derived from glucose^33^, this is consistent with our results.

Unexpectedly, the production of ATP was lower in the *Bp* group. Mitochondria, as the main cellular organ producing ATP, could be damaged due to bacterial toxicity in the *Bp* group^34^. *B. pseudomallei* produce bongkrekic acid (BA) in the host cell during the infection stage, and BA is toxic to the mitochondrial of host cells by inhibiting adenine nucleotide translocase^35^. Which resulted in a significant decrease in ATP production in *Bp* group, but glucose was taken up to help the adhesion and invasion of *B. pseudomallei* HNBP001^36^.

There is a strong correlation between diabetes mellitus and *B. pseudomallei* infection, as 23–60% of patients with melioidosis also have diabetes^3^. But it is currently unknown why are people with diabetes are more susceptible to *B. pseudomallei*^37^. A previous study showed that diabetes results in blunted *B. pseudomallei*-specific cellular responses during acute infection^38^ and possibly due to glucose affecting the thioester bond of neutrophils complement C3 and thereby preventing binding to the bacterial surface^39^. Another study showed that glucose significantly increased adherence and invasion of *B. pseudomallei* to A549 cells^36^, but the mechanism is still unclear. Here, we present a new potential mechanism: abnormal glucose metabolism leads to O-GlcNAc modification abnormally; O-GlcNAcylation may affect the ability of host cells to respond to *B. pseudomallei* through regulating the function and structure of host cell proteins.

O-GlcNAcylation is a posttranslational modification of serine and/or threonine residues of nuclear and cytosolic proteins. Glucose in cells can enter the hexosamine synthesis pathway which converts glucose to uridine-5ʹ-diphosphate-N-acetylglucosa-mine (UDP-GlcNAc), as the substrate for O-GlcNAcylation.Increasing evidence has shown that O-GlcNAcylation regulates various important biological processes, including transcription, stem-cell differentiation, signal transduction, cell cycle progression, and metabolic reprogramming via regulating the function of proteins^40,41^. Aberrant O-GlcNAcylation on various protein substrates has been implicated in many diseases, including cancers^42^, neurodegenerative diseases^43^, diabetes^44^, and immune diseases^45^. The O-GlcNAcylation level was decreased in *B. pseudomallei* HNBP001 infected RAW264.7 cells in our study, which verified that O-GlcNAcylation was associated with melioidosis for the first time. However, the proteins and mechanisms that O-GlcNAcylation regulates in melioidosis warrant further investigation in the future. As an extension of this study, it would be intriguing to investigate the molecular mechanism by which glycometabolism signal pathway is regulated in host cells by *B. pseudomallei* HNBP001.

## Conclusion

Herein, we provide proteomic profiling of *B. pseudomallei* HNBP001-infected mouse macrophage RAW264.7 cells to characterize the cellular response dynamics during infection. The results in this study indicated that glycometabolism changed during *B. pseudomallei* HNBP001 infection, and they provide important insights into the intimate interaction between *B. pseudomallei* and macrophages, which can be further explored to elucidate the cellular mechanisms of *B. pseudomallei* infections.

## Materials and methods

### Bacterial strains, cell lines, and culture conditions

*B. pseudomallei* HNBP001 was isolated from the serum of a melioidosis patient with pneumonia in Hainan and cultured it using Luria–Bertani (LB) broth, according to the protocols earlier described^46^. Bacterial growth curve and viable counts were performed, according to Mariappan et al.^46^, in three independent experiments. In brief, *B. pseudomallei* HNBP001 was cultured in LB liquid medium (GM^+^) at 37 °C, 220 rpm for about 18 h, until the OD reached 0.88. All the experiments of *B. pseudomallei* HNBP001 were performed in P3 laboratory. Mouse macrophages cell line RAW264.7 cell was obtained from ATCC and cultured with DMEM medium (10% FBS), 5%CO_2_ at 37 °C.

### Cell infection of *B. pseudomallei* HNBP001

RAW264.7 cells were cultured in 6 well plate (1.2 × 10^6^ per-well) for 24 h and challenged them via the infected with MOI (multiplicity of infection: the number of *B. pseudomallei /* the number of RAW264.7 cells) 0, 0.5, 1, 10, 20 or 50 of *B. pseudomallei* HNBP001 for 1, 3, 6, 12, or 24 h. Then cells were washed three times with phosphate-buffered saline (PBS) and observed cell morphology by microscope.

### Quantification of viability cell number

The number of cells were counted by cell counter under microscope (Nikon E200) after stained with trypan blue (Beyotime, C0011). Dead cells can be stained blue by Trypan blue due to ruptured membranes, while living cells are colorless. And the percentage of colorless cells (the number of colorless cells / the total number of cells) represents cell viability.

### Quantification of MNGCs

RAW264.7 cells were cultured in 10 cm dish (5 × 10^6^) for 24 h and challenged them via the infected with MOI 50 of *B. pseudomallei* HNBP001 for 0, 1, 3, 6, 12, or 24 h. Then assessment of the percentage of MNGC formation via staining with Giemsa and counted with cell counter under a microscope (Nikon E200), uninfected cells were included as a control. All the living cells can be stained blue with Giemsa, and the number of nuclei can be observed under microscope. The total number of cells and the number of cells within MNGCs (binuclei, trinuclei, multinuclei) were counted with cell counter and examined at least 500 cells in each condition. The percentage of MNGC was calculated according to the following equation: [number of nuclei within MNGCs (binuclei, trinuclei, multinuclei) /total number of nuclei] × 100%. The same experiment performed three times independently.

### Plaque formation assay

RAW264.7 cells were cultured in 10 cm dish (5 × 10^6^) for 24 h and challenged them via the infected with MOI 0, 0.5, 1, 10, 20, 50 of *B. pseudomallei* HNBP001 for 1, 3, 6, 12, or 24 h. Then plaque forming efficiency was calculated by the equation: the number of plaques (plaque-forming units: PFU)/the number of colony-forming units initially used for the infection, assessed by plating dilutions of the bacterial suspension. We defined plaques as defects in the monolayer resulting from MNGCs that have enlarged and are about to burst, as well as MNGCs that have already lysed^47^.

### Protein extraction and trypsin digestion

RAW264.7 cells were cultured in 10 cm dish (5 × 10^6^) for 24 h and challenged them via the infected with MOI 50 of *B. pseudomallei* HNBP001 for 12 h. Then cells were washed with PBS and collected by centrifuging at 1000 × g for 5 min. The volume of 10% TCA/acetone was added to the cell sample for four times and left it at − 20 °C, 4 h. The precipitate was washed three times with cold acetone after centrifuging at 4500 × g for 5 min. The precipitate was lysed in 8 M Urea again, and the supernatant was collected as whole RAW246.7 cells extract. The protein concentration was determined by the BCA kit and digested samples (100 μg) at 4 °C overnight with trypsin in a ratio of 1:50 (trypsin: protein, m/m), then reduced with 5 mM dithiothreitol at 56 °C for 30 min.

The proteins of human serum samples (melioidosis and health controls) were extracted with the ProteoPrep Total Extraction Sample Kit (Merck), and the protein concentration was determined by the BCA kit. The sample (100 μg) was reduced with 5 mM dithiothreitol at 56 °C for 30 min and then used ultrafiltration with 8 M Urea. Then they were digested at 4 °C overnight with trypsin in a ratio of 1:50 (trypsin: protein, m/m).

### TMT labelling

The tryptic peptides were separated in Strata × C18 (Phenomonex) and dissolved them with 0.5 M TEAB. Then the peptides were incubated with TMT in a ratio of 1:2 (peptides: TMT, μg/M) at room temperature for 2 h then dried them in a vacuum concentrator.

### LC–MS/MS analysis

QE Plus mass spectrometer (Thermo Scientific Q Exactive Plus) was used for proteomic analysis. We graded the peptides by high pH reverse HPLC with Agilent 300 Extend C18 chromatographic column (particle size, 5 μm; inner size, 4.6 mm; length, 250 mm). The dried peptide samples were re-dissolved in Solvent A (0.1% formic acid and 2% acetonitrile in water) and loaded them to UHPLC for separation at a flow rate of 500 nL/min for 40 min with phase B (0.1% formic acid and 90% acetonitrile in water). During separation, the phase gradient was 0–20 min, 7–20% B; 20–33 min, 20–32% B; 33–37 min, 32–80% B; 37–40 min, 80% B. After separated by UHPLC, the peptides were loaded into an NSI ion source for ionization at 2.1 kV and analyzed them by Q Exactive™ Plus mass spectrometry. The scan range of the mass spectrometry, scan resolution was 400–1500 m/z and 70,000, respectively. The start of the two-stage mass spectrometry, and the scan resolution was 100 m/z and 17,500, respectively.

To improve the effective utilization of MS, the automatic gain control (AGC) was set to 3E6. Additionally, the signal threshold was set to 7.8E4 ions/s, the maximum loaded time was 50 ms, and the dynamic exclusion time of tandem mass spectrometry was 30 s. Data were acquired using the data dependency scanner.

### Proteome data analysis

The MS/MS data were processed using Maxquant search engine (v.1.5.2.8). Tandem mass spectra were searched against mouse or human Uniprot database concatenated with reverse decoy database. Trypsin/P was specified as cleavage enzyme allowing up to 4 missing cleavages. The mass tolerance for precursor ions was set as 20 ppm in First search and 5 ppm in Main search, and the mass tolerance for fragment ions was set as 0.02 Da. Carbamidomethyl on Cys was specified as fixed modification and acetylation modification and oxidation on Met were specified as variable modifications. FDR was adjusted to < 1% and minimum score for modified peptides was set > 40. Protein expressed profiles were normalized by log2-transformed, and the differentially expressed proteins were identified according to the Holm adjusted *P* < 0.05 in the student’s t-test and Fold Change ≥ 2 or ≤ 0.5 in which Fold Change values were calculated as the ratio of the mean protein expressed levels in Bp group to NC group. GO and KEGG enrichment analysis were performed and visualized by the R package “clusterProfiler” with the default cutoff threshold (Benjamini—Hochberg adjusted *P* < 0.05)^43^. We performed all statistical analyses using R software version 4.0.3 (http://www.rproject.org/).

### Western blot

RAW246.7 cells (*BP* group and NC group) were lysed in SDS lysis buffer (1% SDS, 50 mM Tris–HCl, pH 7.5, 100 mM NaCl, and complete protease inhibitors (Roche)), measured the protein concentration with BCA kit, resolved 40 μg protein on a 4–12% SDS-PAGE gel, transferred it to a nitrocellulose membrane, and immunoblotted it with the indicated antibodies. The lanes containing the target sample are cropped and incubated with corresponding antibodies. We obtained the antibodies used in this study from the following sources: anti-PGM1 (2 μg/ml, Santa Cruz, sc-373796), anti-PGK1 (2 μg/ml, Santa Cruz, sc-130335), anti-PKM (2 μg/ml, Santa Cruz, sc-365684), anti-O-GlcNAc antibody (2 μg/ml, RL2, clone18B10.C7, Thermo Scientific), and anti-Actin (2 μg/ml, Beyotime, AA128).

### ATP measurement

ATP content was measured using an ATP Assay Kit (Beyotime Institute of Biotechnology) according to the manufacturers’ instructions. In brief, RAW246.7 cells (*BP* group and NC group) were cultured in 96-well (1.5 × 10^3^ per-well) for 24 h. Cells were lysed by lysis buffer supported in the kit, then lysis supernatant was collected and precipitation was discarded by centrifuging at 12,000 g, 4 °C for 5 min. Mix 20 µl supernatant with ATP detection reagent at room temperature for 3–5 min, then detected by Infinite 200 PRO Multimode Microplate Reader (-TECAN, Switzerland). The ATP level results were cell number normalized. Extra spaces with parentheses removed here and throughout.

### Glucose uptake assay

The glucose uptake assay was performed according to the Glucose Uptake Assay Kit (Abcam, ab136955). RAW246.7 cells (*BP* group and NC group) were cultured in 96-well (2 × 10^3^ per-well) for 24 h, and starved in serum free DMEM medium overnight. Cells were washed with PBS for 3times and pre-incubating with 100 μl 2%BSA buffer for 40 min at 37 °C. Then added 10 μl of 10 mM 2-DG (total in 100 μl PBS buffer) to cells, incubated for 20 min at 37 °C. Lysed cells with 80 μl extraction buffer by pipetting up and down. The cell lysates were freeze/thaw once, heated at 85 °C for 40 min and cooled on ice for 5 min. Added 10 μl neutralizing buffer to cells lysates. Then 10 μl reaction mix A and 40 μl cell lysates at 37 °C for 1 h, added 90 μl extraction buffer to the each well and heat it at 90 °C for 40 min to degraded any unused NADP left. Added 38 μl reaction mix B to each well and mixed. Measured output at OD 412 nm on a microplate reader (Biotek) in a kinetic mode, every 2–3 min, at 37 °C.

### Statistical analysis

The *P*-values were calculated from a student’s paired *t*-test when comparing within groups and from a student’s unpaired *t-*test when comparing between groups. For analyses where more than one *t*-test was applied to the same data set, statistical analyses were performed via a one-way analysis of variance and Dunnett test comparison post-test.

## Supplementary Information

Supplementary Figure 1.

Supplementary Table 1.

Supplementary Table 2.

Supplementary Table 3.

## Data Availability

All date generated analysed during this study are included in this published article and its supplementary information files.
