# Author Correction: Glycometabolism change during *Burkholderia pseudomallei* infection in RAW264.7 cells by proteomic analysis

**DOI:** 10.1038/s41598-025-05634-5

**Published:** 2025-06-26

**Authors:** Xuexia Li, Yingfei Zeng, Shengnan Guo, Chen Chen, Lin Liu, Qianfeng Xia

**Affiliations:** Key Laboratory of Tropical Translational Medicine of Ministry of Education, NHC Key Laboratory of Tropical Disease Control, School of Tropical Medicine and The Second Afliated Hospital, Hainan Medical University, Haikou, 571199 Hainan China

Correction to: *Scientifc Reports* 10.1038/s41598-022-16716-z, published online: 22 July 2022

The original Article contained errors.

Due to an error while labelling the images during acquisition, in Fig. 1d the image for MOI 0.5-24 h was duplicated from the condition MOI 50-6 h. In addition, for the image MOI 10-12 h an alternative picture of the condition MOI 1-12 h was used and is therefore similar. The image MOI 20-12 h was chosen from a different experimental repeat. Due to the error while labelling the images, some pictures have been analysed twice for the quantification shown in Fig. 1d. While revisiting the original images the Authors noticed that the scale bars in Fig. 1 are incorrect, which also effected the units used in the graph in Fig. 1d.

**Fig. 1:**
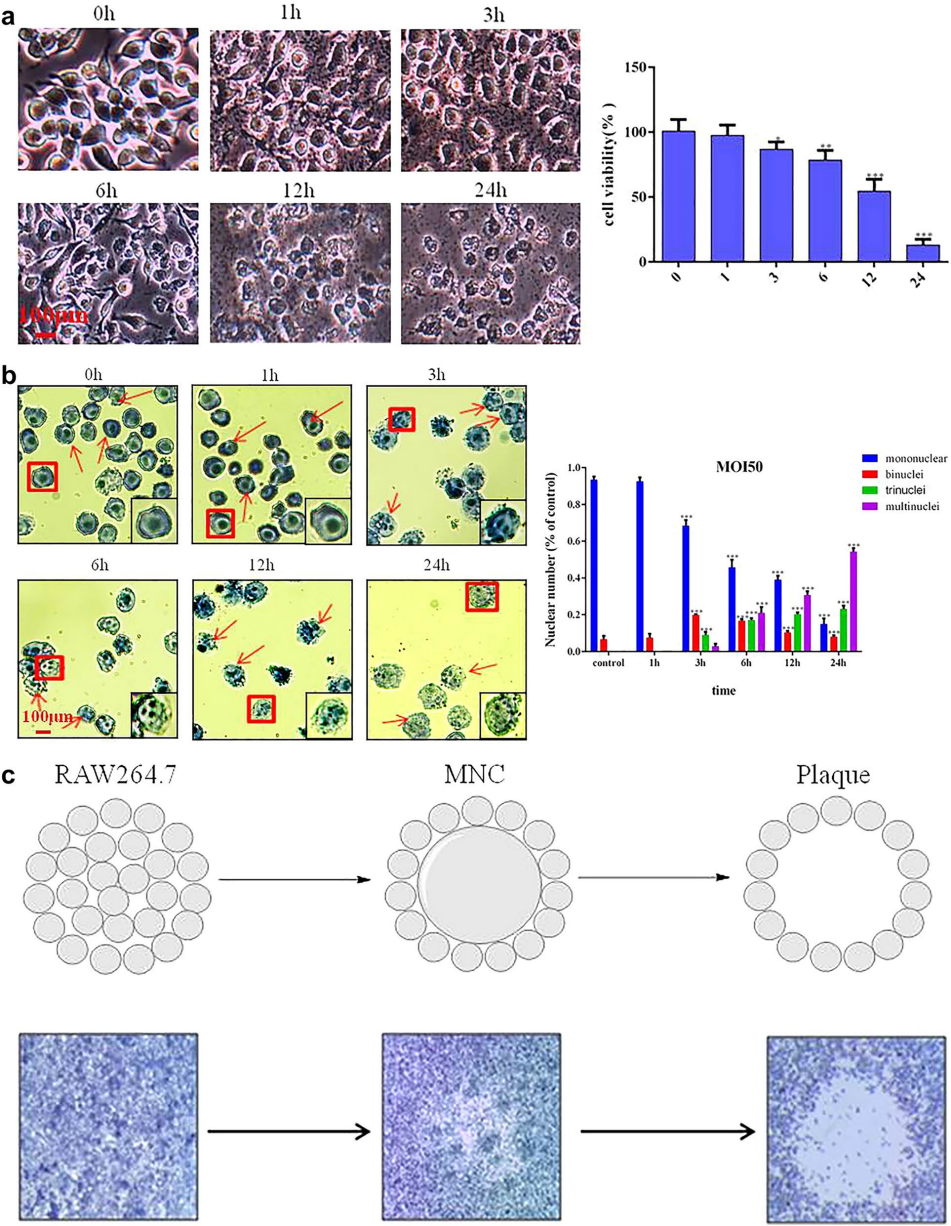

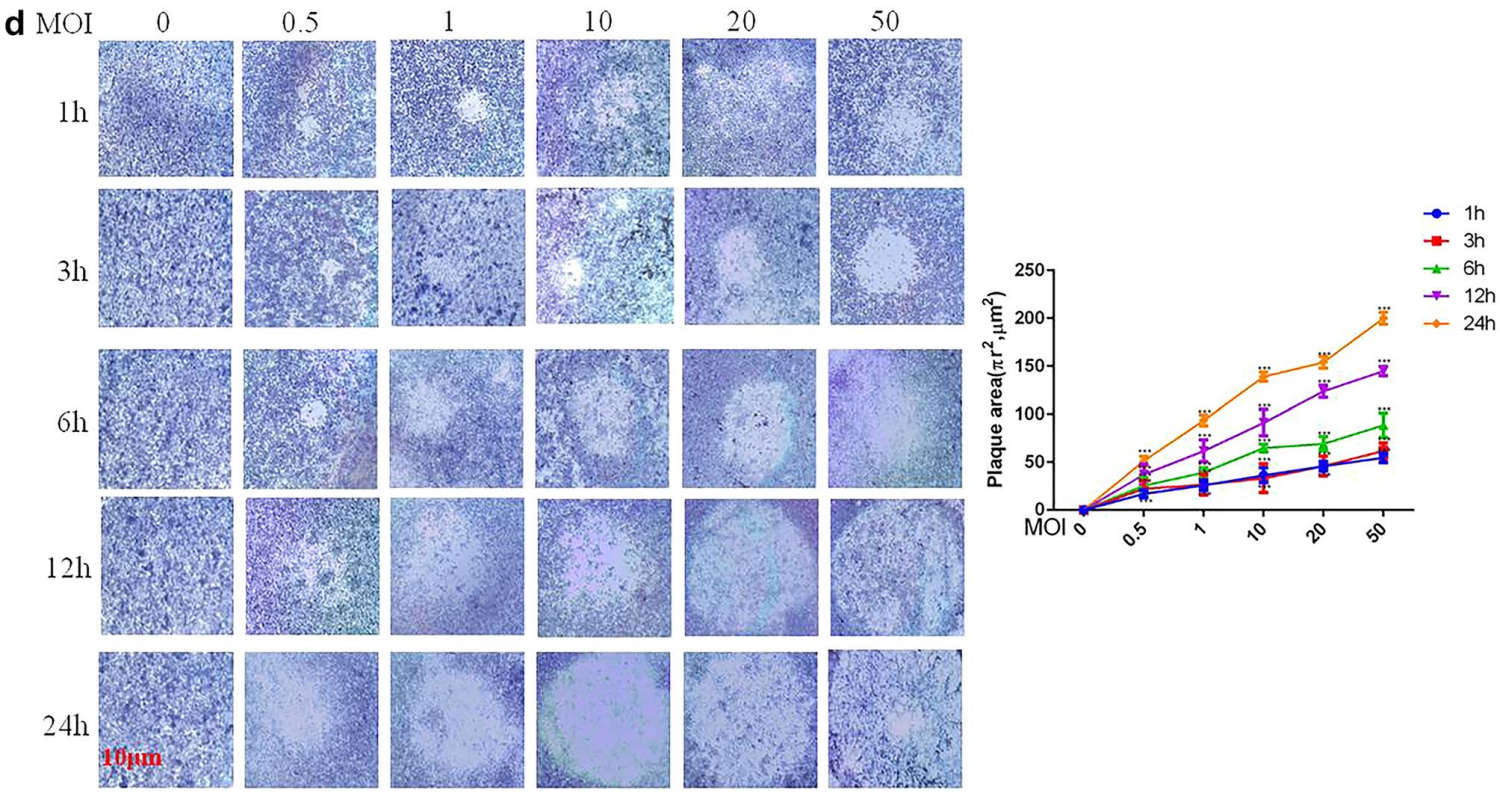
RAW264.7 cell *B. pseudomallei* HNBP001 infection model. (**A**) RAW264.7 cells were infected with *B. pseudomallei* HNBP001 MOI 50 at 0, 1, 3, 6, 12, or 24 h; cell morphology was observed under the microscope; and quantification of cell viability was indicated. (**B**) RAW264.7 cells were stained with Giemsa, and MNGCs were counted. Quantification of MNGCs was indicated. Mononuclear means the number of cells only with one nucleus, binucleus means the number of cells with two nuclei, trinucleus means the number of cells with three nucleus, and the multinucleus means the number of cells with more than three nucleus. (**C**) Cell fusion assay with example well images show RAW264.7 cell monolayers infected with B. pseudomallei HNBP001 and stained with Giemsa. (**D**) The relative abundance and size of plaques while infected with *B. pseudomallei* HNBP001 was assessed at different MOI or times. We conducted the same experiment three times independently.

The scale bars in Fig. 1a,b,d have been updated, the three images in Fig. 1d have been replaced, and the quantification in Fig. 1d was redone.

The original, incorrect, Fig. 1 and the accompanying legend appear below.

The original Article has been corrected.

