# Supplementary material for: Glycometabolism change during Burkholderia pseudomallei infection in RAW264.7 cells by proteomic analysis
Source: Sci Rep. 2022 Jul 22;12:12560. doi: 10.1038/s41598-022-16716-z (PMC9307605; doi:10.1038/s41598-022-16716-z)
Supplement: Supplementary file 1 — Supplementary Figure 1. [file 41598_2022_16716_MOESM1_ESM.doc]

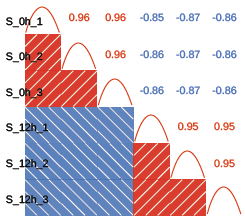


LC-MS/MS

Fig S1. The heat map of all the samples by calculating Pearson correlation coefficients in pairs. When the coefficient is closer to -1, the correlation is negative, and, when the coefficient is closer to 1, the correlation is positive.
