# Supplementary material for: Glycometabolism change during Burkholderia pseudomallei infection in RAW264.7 cells by proteomic analysis
Source: Sci Rep. 2022 Jul 22;12:12560. doi: 10.1038/s41598-022-16716-z (PMC9307605; doi:10.1038/s41598-022-16716-z)
Supplement: Supplementary file 2 — Supplementary Table 1. [file 41598_2022_16716_MOESM2_ESM.doc]

Supplementary table 1: Differentially expressed proteins in RAW264.7 cell with *B. pseudomallei* HNBP001 infection or uninfection

The protein accession column denotes the Uniport protein ID. S_0h represents RAW264.7 cells without *B. pseudomallei* HNBP001 infection and S_12h represents RAW164.7 cells infected with *B. pseudomallei* HNBP001 (MOI 50) for 12h, respectively. And the postfix 1,2 and 3 represent three biological repeats. Differentially expressed proteins were identified by the Student’s T test and *P* values were adjusted by the Holm method. Fold change (FC), S_12h/S_0h Ratio of protein, means the protein expression level altered in S_12h versus S _0h. We identified 811 differentially expressed proteins with FC > 2 or FC < 1/2 and FDR < 0.05.

| **Protein accession** | **Protein description** | **Gene name** | **S_0h_1** | **S_0h_2** | **S_0h_3** | **S_12h_1** | **S_12h_2** | **S_12h_3** | **S_12h/S_0h Ratio** | **S_12h/S_0h P value** | **FDR** | **Diff_Type** |
| --- | --- | --- | --- | --- | --- | --- | --- | --- | --- | --- | --- | --- |
| O35215 | D-dopachrome decarboxylase OS=Mus musculus OX=10090 GN=Ddt PE=1 SV=3 | Ddt | 0.165 | 0.182 | 0.177 | 1.844 | 1.758 | 1.76 | 10.233 | 2.47E-07 | 0.001368519 | Up |
| Q9EQU5 | Protein SET OS=Mus musculus OX=10090 GN=Set PE=1 SV=1 | Set | 0.189 | 0.2 | 0.193 | 1.908 | 1.64 | 1.664 | 8.955 | 1.75E-06 | 0.009238903 | Up |
| Q8CHP8 | Glycerol-3-phosphate phosphatase OS=Mus musculus OX=10090 GN=Pgp PE=1 SV=1 | Pgp | 0.192 | 0.235 | 0.209 | 1.856 | 1.72 | 1.767 | 8.401 | 4.46E-06 | 0.02241178 | Up |
| Q99PT1 | Rho GDP-dissociation inhibitor 1 OS=Mus musculus OX=10090 GN=Arhgdia PE=1 SV=3 | Arhgdia | 0.214 | 0.223 | 0.205 | 1.771 | 1.847 | 1.645 | 8.198 | 9.22E-07 | 0.004969418 | Up |
| Q6B966 | NACHT, LRR and PYD domains-containing protein 14 OS=Mus musculus OX=10090 GN=Nlrp14 PE=1 SV=2 | Nlrp14 | 0.24 | 0.212 | 0.215 | 1.888 | 1.744 | 1.753 | 8.073 | 1.51E-06 | 0.008014768 | Up |
| Q9JM14 | 5'(3')-deoxyribonucleotidase, cytosolic type OS=Mus musculus OX=10090 GN=Nt5c PE=1 SV=1 | Nt5c | 0.223 | 0.221 | 0.25 | 1.977 | 1.747 | 1.721 | 7.846 | 4.07E-06 | 0.020593837 | Up |
| P07901 | Heat shock protein HSP 90-alpha OS=Mus musculus OX=10090 GN=Hsp90aa1 PE=1 SV=4 | Hsp90aa1 | 0.231 | 0.238 | 0.254 | 1.74 | 1.728 | 1.738 | 7.201 | 2.46E-07 | 0.001361296 | Up |
| P08228 | Superoxide dismutase [Cu-Zn] OS=Mus musculus OX=10090 GN=Sod1 PE=1 SV=2 | Sod1 | 0.26 | 0.232 | 0.248 | 1.791 | 1.564 | 1.748 | 6.896 | 3.45E-06 | 0.017627146 | Up |
| Q01768 | Nucleoside diphosphate kinase B OS=Mus musculus OX=10090 GN=Nme2 PE=1 SV=1 | Nme2 | 0.255 | 0.274 | 0.222 | 1.83 | 1.643 | 1.686 | 6.87 | 1.02E-05 | 0.047738604 | Up |
| Q62433 | Protein NDRG1 OS=Mus musculus OX=10090 GN=Ndrg1 PE=1 SV=1 | Ndrg1 | 0.262 | 0.268 | 0.246 | 1.828 | 1.731 | 1.694 | 6.769 | 6.12E-07 | 0.003334039 | Up |
| P00493 | Hypoxanthine-guanine phosphoribosyltransferase OS=Mus musculus OX=10090 GN=Hprt1 PE=1 SV=3 | Hprt1 | 0.265 | 0.258 | 0.243 | 1.703 | 1.818 | 1.649 | 6.749 | 9.87E-07 | 0.005306037 | Up |
| Q9ER72 | Cysteine--tRNA ligase, cytoplasmic OS=Mus musculus OX=10090 GN=Cars1 PE=1 SV=2 | Cars1 | 0.251 | 0.298 | 0.242 | 1.722 | 1.733 | 1.678 | 6.489 | 8.57E-06 | 0.040925045 | Up |
| Q9D0F9 | Phosphoglucomutase-1 OS=Mus musculus OX=10090 GN=Pgm1 PE=1 SV=4 | Pgm1 | 0.255 | 0.277 | 0.277 | 1.791 | 1.764 | 1.692 | 6.486 | 5.40E-07 | 0.002948511 | Up |
| Q9D8L5 | Coiled-coil domain-containing protein 91 OS=Mus musculus OX=10090 GN=Ccdc91 PE=1 SV=2 | Ccdc91 | 0.27 | 0.299 | 0.241 | 1.841 | 1.685 | 1.716 | 6.472 | 1.03E-05 | 0.048331873 | Up |
| Q8BG73 | SH3 domain-binding glutamic acid-rich-like protein 2 OS=Mus musculus OX=10090 GN=Sh3bgrl2 PE=1 SV=1 | Sh3bgrl2 | 0.246 | 0.292 | 0.277 | 1.676 | 1.802 | 1.731 | 6.391 | 4.54E-06 | 0.022777528 | Up |
| Q5SUE8 | Ankyrin repeat domain-containing protein 40 OS=Mus musculus OX=10090 GN=Ankrd40 PE=1 SV=1 | Ankrd40 | 0.274 | 0.263 | 0.283 | 1.928 | 1.748 | 1.551 | 6.374 | 9.89E-06 | 0.046551524 | Up |
| P26638 | Serine--tRNA ligase, cytoplasmic OS=Mus musculus OX=10090 GN=Sars1 PE=1 SV=3 | Sars1 | 0.275 | 0.256 | 0.284 | 1.711 | 1.742 | 1.716 | 6.342 | 4.85E-07 | 0.002657348 | Up |
| P22907 | Porphobilinogen deaminase OS=Mus musculus OX=10090 GN=Hmbs PE=1 SV=2 | Hmbs | 0.289 | 0.26 | 0.279 | 1.799 | 1.696 | 1.673 | 6.242 | 1.14E-06 | 0.006087151 | Up |
| Q8R016 | Bleomycin hydrolase OS=Mus musculus OX=10090 GN=Blmh PE=1 SV=1 | Blmh | 0.273 | 0.298 | 0.268 | 1.792 | 1.682 | 1.708 | 6.176 | 1.12E-06 | 0.005994173 | Up |
| P63101 | 14-3-3 protein zeta/delta OS=Mus musculus OX=10090 GN=Ywhaz PE=1 SV=1 | Ywhaz | 0.272 | 0.295 | 0.275 | 1.741 | 1.645 | 1.812 | 6.173 | 1.12E-06 | 0.005997122 | Up |
| P50396 | Rab GDP dissociation inhibitor alpha OS=Mus musculus OX=10090 GN=Gdi1 PE=1 SV=3 | Gdi1 | 0.293 | 0.294 | 0.252 | 1.742 | 1.71 | 1.725 | 6.17 | 3.69E-06 | 0.018776515 | Up |
| P45376 | Aldo-keto reductase family 1 member B1 OS=Mus musculus OX=10090 GN=Akr1b1 PE=1 SV=3 | Akr1b1 | 0.297 | 0.263 | 0.268 | 1.659 | 1.741 | 1.659 | 6.11 | 1.58E-06 | 0.008361881 | Up |
| P34022 | Ran-specific GTPase-activating protein OS=Mus musculus OX=10090 GN=Ranbp1 PE=1 SV=2 | Ranbp1 | 0.308 | 0.275 | 0.262 | 1.806 | 1.657 | 1.688 | 6.096 | 4.95E-06 | 0.024693105 | Up |
| P70349 | Histidine triad nucleotide-binding protein 1 OS=Mus musculus OX=10090 GN=Hint1 PE=1 SV=3 | Hint1 | 0.29 | 0.286 | 0.284 | 1.85 | 1.714 | 1.674 | 6.091 | 5.06E-07 | 0.002769996 | Up |
| P55264 | Adenosine kinase OS=Mus musculus OX=10090 GN=Adk PE=1 SV=2 | Adk | 0.276 | 0.275 | 0.263 | 1.562 | 1.742 | 1.624 | 6.054 | 9.04E-07 | 0.004874849 | Up |
| P24527 | Leukotriene A-4 hydrolase OS=Mus musculus OX=10090 GN=Lta4h PE=1 SV=4 | Lta4h | 0.282 | 0.268 | 0.282 | 1.7 | 1.661 | 1.646 | 6.018 | 8.36E-08 | 0.000466296 | Up |
| P97371 | Proteasome activator complex subunit 1 OS=Mus musculus OX=10090 GN=Psme1 PE=1 SV=2 | Psme1 | 0.311 | 0.29 | 0.262 | 1.824 | 1.592 | 1.705 | 5.934 | 9.54E-06 | 0.045054396 | Up |
| Q9QYG0 | Protein NDRG2 OS=Mus musculus OX=10090 GN=Ndrg2 PE=1 SV=1 | Ndrg2 | 0.295 | 0.27 | 0.306 | 1.795 | 1.684 | 1.679 | 5.922 | 2.05E-06 | 0.010764908 | Up |
| Q99MD9 | Nuclear autoantigenic sperm protein OS=Mus musculus OX=10090 GN=Nasp PE=1 SV=2 | Nasp | 0.297 | 0.291 | 0.264 | 1.793 | 1.624 | 1.624 | 5.917 | 3.47E-06 | 0.017709293 | Up |
| Q99L47 | Hsc70-interacting protein OS=Mus musculus OX=10090 GN=St13 PE=1 SV=1 | St13 | 0.279 | 0.316 | 0.29 | 1.838 | 1.707 | 1.661 | 5.882 | 3.12E-06 | 0.016005024 | Up |
| P14152 | Malate dehydrogenase, cytoplasmic OS=Mus musculus OX=10090 GN=Mdh1 PE=1 SV=3 | Mdh1 | 0.29 | 0.283 | 0.278 | 1.683 | 1.703 | 1.61 | 5.871 | 1.19E-07 | 0.000665173 | Up |
| P11499 | Heat shock protein HSP 90-beta OS=Mus musculus OX=10090 GN=Hsp90ab1 PE=1 SV=3 | Hsp90ab1 | 0.294 | 0.282 | 0.289 | 1.668 | 1.7 | 1.707 | 5.867 | 2.37E-08 | 0.000132785 | Up |
| Q61035 | Histidine--tRNA ligase, cytoplasmic OS=Mus musculus OX=10090 GN=Hars1 PE=1 SV=2 | Hars1 | 0.316 | 0.31 | 0.272 | 1.734 | 1.696 | 1.739 | 5.756 | 3.30E-06 | 0.01688749 | Up |
| Q64514 | Tripeptidyl-peptidase 2 OS=Mus musculus OX=10090 GN=Tpp2 PE=1 SV=3 | Tpp2 | 0.312 | 0.304 | 0.274 | 1.699 | 1.754 | 1.669 | 5.755 | 2.02E-06 | 0.010636169 | Up |
| P17751 | Triosephosphate isomerase OS=Mus musculus OX=10090 GN=Tpi1 PE=1 SV=4 | Tpi1 | 0.295 | 0.287 | 0.306 | 1.801 | 1.661 | 1.628 | 5.732 | 1.09E-06 | 0.005821174 | Up |
| Q8BH58 | TIP41-like protein OS=Mus musculus OX=10090 GN=Tiprl PE=1 SV=1 | Tiprl | 0.298 | 0.304 | 0.291 | 1.798 | 1.74 | 1.558 | 5.707 | 2.71E-06 | 0.014022532 | Up |
| P17182 | Alpha-enolase OS=Mus musculus OX=10090 GN=Eno1 PE=1 SV=3 | Eno1 | 0.32 | 0.3 | 0.263 | 1.751 | 1.646 | 1.641 | 5.706 | 9.18E-06 | 0.043542472 | Up |
| Q60864 | Stress-induced-phosphoprotein 1 OS=Mus musculus OX=10090 GN=Stip1 PE=1 SV=1 | Stip1 | 0.328 | 0.293 | 0.276 | 1.818 | 1.653 | 1.636 | 5.693 | 8.82E-06 | 0.041985221 | Up |
| Q9CZ44 | NSFL1 cofactor p47 OS=Mus musculus OX=10090 GN=Nsfl1c PE=1 SV=1 | Nsfl1c | 0.3 | 0.31 | 0.294 | 1.906 | 1.557 | 1.65 | 5.656 | 9.88E-06 | 0.046505906 | Up |
| P24288 | Branched-chain-amino-acid aminotransferase, cytosolic OS=Mus musculus OX=10090 GN=Bcat1 PE=1 SV=2 | Bcat1 | 0.318 | 0.281 | 0.313 | 1.747 | 1.738 | 1.641 | 5.621 | 2.45E-06 | 0.012764251 | Up |
| Q7TPR4 | Alpha-actinin-1 OS=Mus musculus OX=10090 GN=Actn1 PE=1 SV=1 | Actn1 | 0.325 | 0.288 | 0.305 | 1.748 | 1.578 | 1.738 | 5.516 | 3.78E-06 | 0.019181775 | Up |
| Q9CWJ9 | Bifunctional purine biosynthesis protein PURH OS=Mus musculus OX=10090 GN=Atic PE=1 SV=2 | Atic | 0.315 | 0.304 | 0.307 | 1.608 | 1.8 | 1.647 | 5.459 | 1.20E-06 | 0.006423754 | Up |
| P31230 | Aminoacyl tRNA synthase complex-interacting multifunctional protein 1 OS=Mus musculus OX=10090 GN=Aimp1 PE=1 SV=2 | Aimp1 | 0.306 | 0.302 | 0.319 | 1.758 | 1.708 | 1.581 | 5.444 | 1.18E-06 | 0.006327005 | Up |
| Q60604 | Adseverin OS=Mus musculus OX=10090 GN=Scin PE=1 SV=3 | Scin | 0.332 | 0.308 | 0.29 | 1.681 | 1.748 | 1.629 | 5.439 | 2.74E-06 | 0.014199113 | Up |
| P15532 | Nucleoside diphosphate kinase A OS=Mus musculus OX=10090 GN=Nme1 PE=1 SV=1 | Nme1 | 0.339 | 0.282 | 0.313 | 1.717 | 1.65 | 1.707 | 5.433 | 6.47E-06 | 0.031627225 | Up |
| O08800 | Serpin B8 OS=Mus musculus OX=10090 GN=Serpinb8 PE=1 SV=2 | Serpinb8 | 0.328 | 0.294 | 0.324 | 1.696 | 1.786 | 1.637 | 5.411 | 2.48E-06 | 0.012890052 | Up |
| P06151 | L-lactate dehydrogenase A chain OS=Mus musculus OX=10090 GN=Ldha PE=1 SV=3 | Ldha | 0.313 | 0.309 | 0.308 | 1.619 | 1.749 | 1.64 | 5.385 | 2.64E-07 | 0.001462986 | Up |
| Q7TSV4 | Phosphoglucomutase-2 OS=Mus musculus OX=10090 GN=Pgm2 PE=1 SV=1 | Pgm2 | 0.312 | 0.312 | 0.316 | 1.698 | 1.688 | 1.668 | 5.377 | 1.55E-09 | 8.71E-06 | Up |
| Q6URW6 | Myosin-14 OS=Mus musculus OX=10090 GN=Myh14 PE=1 SV=1 | Myh14 | 0.327 | 0.33 | 0.298 | 1.745 | 1.678 | 1.664 | 5.327 | 1.24E-06 | 0.006631839 | Up |
| Q61316 | Heat shock 70 kDa protein 4 OS=Mus musculus OX=10090 GN=Hspa4 PE=1 SV=1 | Hspa4 | 0.316 | 0.32 | 0.314 | 1.733 | 1.676 | 1.645 | 5.32 | 5.33E-08 | 0.000297892 | Up |
| O88844 | Isocitrate dehydrogenase [NADP] cytoplasmic OS=Mus musculus OX=10090 GN=Idh1 PE=1 SV=2 | Idh1 | 0.327 | 0.314 | 0.296 | 1.531 | 1.791 | 1.622 | 5.276 | 6.72E-06 | 0.032776374 | Up |
| P57780 | Alpha-actinin-4 OS=Mus musculus OX=10090 GN=Actn4 PE=1 SV=1 | Actn4 | 0.318 | 0.33 | 0.304 | 1.697 | 1.649 | 1.659 | 5.257 | 3.22E-07 | 0.001777897 | Up |
| Q61233 | Plastin-2 OS=Mus musculus OX=10090 GN=Lcp1 PE=1 SV=4 | Lcp1 | 0.321 | 0.335 | 0.312 | 1.711 | 1.644 | 1.656 | 5.177 | 2.74E-07 | 0.001516028 | Up |
| P61971 | Nuclear transport factor 2 OS=Mus musculus OX=10090 GN=Nutf2 PE=1 SV=1 | Nutf2 | 0.325 | 0.336 | 0.306 | 1.711 | 1.674 | 1.587 | 5.142 | 1.30E-06 | 0.00690784 | Up |
| O35685 | Nuclear migration protein nudC OS=Mus musculus OX=10090 GN=Nudc PE=1 SV=1 | Nudc | 0.317 | 0.352 | 0.312 | 1.691 | 1.726 | 1.613 | 5.127 | 2.81E-06 | 0.014506296 | Up |
| Q93092 | Transaldolase OS=Mus musculus OX=10090 GN=Taldo1 PE=1 SV=2 | Taldo1 | 0.326 | 0.332 | 0.321 | 1.697 | 1.624 | 1.635 | 5.062 | 6.88E-08 | 0.000383949 | Up |
| Q9D2Z4 | Sentrin-specific protease 8 OS=Mus musculus OX=10090 GN=Senp8 PE=1 SV=2 | Senp8 | 0.308 | 0.341 | 0.346 | 1.672 | 1.723 | 1.64 | 5.06 | 2.06E-06 | 0.010804018 | Up |
| P56873 | Protein ZNRD2 OS=Mus musculus OX=10090 GN=Znrd2 PE=1 SV=1 | Znrd2 | 0.345 | 0.32 | 0.337 | 1.797 | 1.65 | 1.611 | 5.048 | 2.21E-06 | 0.011562124 | Up |
| Q60605 | Myosin light polypeptide 6 OS=Mus musculus OX=10090 GN=Myl6 PE=1 SV=3 | Myl6 | 0.346 | 0.328 | 0.329 | 1.828 | 1.533 | 1.686 | 5.032 | 7.32E-06 | 0.03541751 | Up |
| Q3TDX8 | Cytochrome b5 reductase 4 OS=Mus musculus OX=10090 GN=Cyb5r4 PE=2 SV=3 | Cyb5r4 | 0.324 | 0.359 | 0.316 | 1.645 | 1.732 | 1.649 | 5.031 | 2.85E-06 | 0.014706289 | Up |
| Q60854 | Serpin B6 OS=Mus musculus OX=10090 GN=Serpinb6 PE=1 SV=1 | Serpinb6 | 0.323 | 0.312 | 0.364 | 1.618 | 1.758 | 1.634 | 5.015 | 7.21E-06 | 0.034905971 | Up |
| P40124 | Adenylyl cyclase-associated protein 1 OS=Mus musculus OX=10090 GN=Cap1 PE=1 SV=4 | Cap1 | 0.314 | 0.335 | 0.345 | 1.557 | 1.775 | 1.6 | 4.962 | 5.09E-06 | 0.025321692 | Up |
| P18760 | Cofilin-1 OS=Mus musculus OX=10090 GN=Cfl1 PE=1 SV=3 | Cfl1 | 0.344 | 0.344 | 0.32 | 1.704 | 1.704 | 1.586 | 4.954 | 1.21E-06 | 0.006484303 | Up |
| Q8BHG1 | Nardilysin OS=Mus musculus OX=10090 GN=Nrdc PE=1 SV=1 | Nrdc | 0.328 | 0.351 | 0.302 | 1.644 | 1.68 | 1.53 | 4.948 | 6.57E-06 | 0.032074581 | Up |
| P09411 | Phosphoglycerate kinase 1 OS=Mus musculus OX=10090 GN=Pgk1 PE=1 SV=4 | Pgk1 | 0.347 | 0.34 | 0.326 | 1.704 | 1.677 | 1.618 | 4.935 | 3.02E-07 | 0.001669392 | Up |
| Q61074 | Protein phosphatase 1G OS=Mus musculus OX=10090 GN=Ppm1g PE=1 SV=3 | Ppm1g | 0.344 | 0.346 | 0.334 | 1.816 | 1.62 | 1.606 | 4.924 | 2.64E-06 | 0.01370577 | Up |
| Q8BGQ7 | Alanine--tRNA ligase, cytoplasmic OS=Mus musculus OX=10090 GN=Aars1 PE=1 SV=1 | Aars1 | 0.354 | 0.311 | 0.322 | 1.656 | 1.594 | 1.594 | 4.908 | 2.56E-06 | 0.013289443 | Up |
| P28656 | Nucleosome assembly protein 1-like 1 OS=Mus musculus OX=10090 GN=Nap1l1 PE=1 SV=2 | Nap1l1 | 0.35 | 0.346 | 0.346 | 1.84 | 1.608 | 1.641 | 4.884 | 2.99E-06 | 0.01538346 | Up |
| Q11136 | Xaa-Pro dipeptidase OS=Mus musculus OX=10090 GN=Pepd PE=1 SV=3 | Pepd | 0.348 | 0.331 | 0.33 | 1.643 | 1.727 | 1.541 | 4.867 | 1.83E-06 | 0.009641441 | Up |
| Q9JMH6 | Thioredoxin reductase 1, cytoplasmic OS=Mus musculus OX=10090 GN=Txnrd1 PE=1 SV=3 | Txnrd1 | 0.332 | 0.366 | 0.333 | 1.716 | 1.71 | 1.585 | 4.86 | 2.75E-06 | 0.014204437 | Up |
| Q00612 | Glucose-6-phosphate 1-dehydrogenase X OS=Mus musculus OX=10090 GN=G6pdx PE=1 SV=3 | G6pdx | 0.315 | 0.348 | 0.366 | 1.572 | 1.763 | 1.621 | 4.816 | 9.46E-06 | 0.044698985 | Up |
| O08759 | Ubiquitin-protein ligase E3A OS=Mus musculus OX=10090 GN=Ube3a PE=1 SV=2 | Ube3a | 0.35 | 0.323 | 0.351 | 1.645 | 1.654 | 1.616 | 4.8 | 6.19E-07 | 0.003367043 | Up |
| Q9D0R2 | Threonine--tRNA ligase 1, cytoplasmic OS=Mus musculus OX=10090 GN=Tars1 PE=1 SV=2 | Tars1 | 0.341 | 0.34 | 0.357 | 1.675 | 1.679 | 1.623 | 4.795 | 1.35E-07 | 0.000751608 | Up |
| Q9DBJ1 | Phosphoglycerate mutase 1 OS=Mus musculus OX=10090 GN=Pgam1 PE=1 SV=3 | Pgam1 | 0.335 | 0.356 | 0.343 | 1.596 | 1.628 | 1.712 | 4.774 | 5.63E-07 | 0.003073017 | Up |
| P03958 | Adenosine deaminase OS=Mus musculus OX=10090 GN=Ada PE=1 SV=3 | Ada | 0.355 | 0.369 | 0.318 | 1.65 | 1.717 | 1.596 | 4.763 | 5.91E-06 | 0.029108905 | Up |
| Q9D8N0 | Elongation factor 1-gamma OS=Mus musculus OX=10090 GN=Eef1g PE=1 SV=3 | Eef1g | 0.351 | 0.344 | 0.344 | 1.648 | 1.634 | 1.666 | 4.762 | 5.93E-09 | 3.33E-05 | Up |
| Q91UZ5 | Inositol monophosphatase 2 OS=Mus musculus OX=10090 GN=Impa2 PE=1 SV=1 | Impa2 | 0.342 | 0.355 | 0.355 | 1.738 | 1.615 | 1.641 | 4.747 | 4.32E-07 | 0.002372066 | Up |
| O88447 | Kinesin light chain 1 OS=Mus musculus OX=10090 GN=Klc1 PE=1 SV=3 | Klc1 | 0.35 | 0.367 | 0.342 | 1.697 | 1.737 | 1.593 | 4.747 | 1.23E-06 | 0.006569003 | Up |
| Q6P1B1 | Xaa-Pro aminopeptidase 1 OS=Mus musculus OX=10090 GN=Xpnpep1 PE=1 SV=1 | Xpnpep1 | 0.357 | 0.335 | 0.35 | 1.646 | 1.673 | 1.618 | 4.738 | 2.04E-07 | 0.00112995 | Up |
| Q9QUR6 | Prolyl endopeptidase OS=Mus musculus OX=10090 GN=Prep PE=1 SV=1 | Prep | 0.351 | 0.377 | 0.32 | 1.635 | 1.634 | 1.655 | 4.698 | 5.32E-06 | 0.026405039 | Up |
| Q9DBP5 | UMP-CMP kinase OS=Mus musculus OX=10090 GN=Cmpk1 PE=1 SV=1 | Cmpk1 | 0.359 | 0.344 | 0.344 | 1.722 | 1.586 | 1.6 | 4.688 | 8.20E-07 | 0.004430095 | Up |
| Q8C1A5 | Thimet oligopeptidase OS=Mus musculus OX=10090 GN=Thop1 PE=1 SV=1 | Thop1 | 0.385 | 0.325 | 0.339 | 1.587 | 1.696 | 1.635 | 4.688 | 9.11E-06 | 0.043251565 | Up |
| Q8BP47 | Asparagine--tRNA ligase, cytoplasmic OS=Mus musculus OX=10090 GN=NARS1 PE=1 SV=2 | NARS1 | 0.364 | 0.342 | 0.352 | 1.592 | 1.664 | 1.687 | 4.672 | 4.21E-07 | 0.002313758 | Up |
| Q99J36 | THUMP domain-containing protein 1 OS=Mus musculus OX=10090 GN=Thumpd1 PE=1 SV=1 | Thumpd1 | 0.35 | 0.38 | 0.328 | 1.657 | 1.679 | 1.585 | 4.651 | 4.77E-06 | 0.023834032 | Up |
| Q78ZA7 | Nucleosome assembly protein 1-like 4 OS=Mus musculus OX=10090 GN=Nap1l4 PE=1 SV=1 | Nap1l4 | 0.359 | 0.341 | 0.378 | 1.66 | 1.688 | 1.637 | 4.624 | 1.01E-06 | 0.005412933 | Up |
| Q8BU30 | Isoleucine--tRNA ligase, cytoplasmic OS=Mus musculus OX=10090 GN=Iars1 PE=1 SV=2 | Iars1 | 0.392 | 0.334 | 0.336 | 1.618 | 1.634 | 1.646 | 4.612 | 8.31E-06 | 0.039794744 | Up |
| Q9CPV4 | Glyoxalase domain-containing protein 4 OS=Mus musculus OX=10090 GN=Glod4 PE=1 SV=1 | Glod4 | 0.36 | 0.358 | 0.357 | 1.704 | 1.672 | 1.556 | 4.588 | 6.64E-07 | 0.0036056 | Up |
| Q9DCJ9 | N-acetylneuraminate lyase OS=Mus musculus OX=10090 GN=Npl PE=1 SV=1 | Npl | 0.352 | 0.351 | 0.368 | 1.651 | 1.578 | 1.681 | 4.585 | 3.86E-07 | 0.002121692 | Up |
| Q8VI36 | Paxillin OS=Mus musculus OX=10090 GN=Pxn PE=1 SV=1 | Pxn | 0.348 | 0.361 | 0.336 | 1.73 | 1.576 | 1.479 | 4.579 | 6.99E-06 | 0.033946577 | Up |
| Q8CGC7 | Bifunctional glutamate/proline--tRNA ligase OS=Mus musculus OX=10090 GN=Eprs1 PE=1 SV=4 | Eprs1 | 0.369 | 0.368 | 0.354 | 1.662 | 1.64 | 1.658 | 4.546 | 4.40E-08 | 0.000246049 | Up |
| P68372 | Tubulin beta-4B chain OS=Mus musculus OX=10090 GN=Tubb4b PE=1 SV=1 | Tubb4b | 0.386 | 0.351 | 0.348 | 1.67 | 1.634 | 1.617 | 4.535 | 1.62E-06 | 0.008587606 | Up |
| Q9CPU0 | Lactoylglutathione lyase OS=Mus musculus OX=10090 GN=Glo1 PE=1 SV=3 | Glo1 | 0.362 | 0.342 | 0.393 | 1.684 | 1.657 | 1.598 | 4.502 | 4.05E-06 | 0.020464713 | Up |
| Q61081 | Hsp90 co-chaperone Cdc37 OS=Mus musculus OX=10090 GN=Cdc37 PE=1 SV=1 | Cdc37 | 0.385 | 0.366 | 0.375 | 1.684 | 1.74 | 1.638 | 4.496 | 3.15E-07 | 0.001741583 | Up |
| Q8VBT9 | Tether containing UBX domain for GLUT4 OS=Mus musculus OX=10090 GN=Aspscr1 PE=1 SV=1 | Aspscr1 | 0.379 | 0.387 | 0.332 | 1.654 | 1.686 | 1.594 | 4.494 | 7.70E-06 | 0.037106261 | Up |
| Q80X90 | Filamin-B OS=Mus musculus OX=10090 GN=Flnb PE=1 SV=3 | Flnb | 0.374 | 0.374 | 0.355 | 1.662 | 1.682 | 1.594 | 4.477 | 3.82E-07 | 0.002101406 | Up |
| Q9CR16 | Peptidyl-prolyl cis-trans isomerase D OS=Mus musculus OX=10090 GN=Ppid PE=1 SV=3 | Ppid | 0.383 | 0.37 | 0.346 | 1.595 | 1.733 | 1.582 | 4.468 | 3.59E-06 | 0.018286974 | Up |
| P47753 | F-actin-capping protein subunit alpha-1 OS=Mus musculus OX=10090 GN=Capza1 PE=1 SV=4 | Capza1 | 0.401 | 0.37 | 0.345 | 1.728 | 1.623 | 1.628 | 4.461 | 6.30E-06 | 0.030876355 | Up |
| Q9R1T2 | SUMO-activating enzyme subunit 1 OS=Mus musculus OX=10090 GN=Sae1 PE=1 SV=1 | Sae1 | 0.373 | 0.372 | 0.358 | 1.611 | 1.645 | 1.622 | 4.422 | 5.60E-08 | 0.000312661 | Up |
| Q9D0I9 | Arginine--tRNA ligase, cytoplasmic OS=Mus musculus OX=10090 GN=Rars1 PE=1 SV=2 | Rars1 | 0.389 | 0.365 | 0.354 | 1.622 | 1.66 | 1.612 | 4.417 | 8.88E-07 | 0.004793251 | Up |
| P61087 | Ubiquitin-conjugating enzyme E2 K OS=Mus musculus OX=10090 GN=Ube2k PE=1 SV=3 | Ube2k | 0.364 | 0.372 | 0.388 | 1.576 | 1.778 | 1.587 | 4.396 | 4.40E-06 | 0.022115658 | Up |
| P47757 | F-actin-capping protein subunit beta OS=Mus musculus OX=10090 GN=Capzb PE=1 SV=3 | Capzb | 0.394 | 0.383 | 0.338 | 1.699 | 1.606 | 1.588 | 4.388 | 8.73E-06 | 0.041610717 | Up |
| Q64331 | Unconventional myosin-VI OS=Mus musculus OX=10090 GN=Myo6 PE=1 SV=1 | Myo6 | 0.385 | 0.364 | 0.358 | 1.61 | 1.726 | 1.506 | 4.374 | 5.21E-06 | 0.025905534 | Up |
| O88398 | Advillin OS=Mus musculus OX=10090 GN=Avil PE=1 SV=2 | Avil | 0.363 | 0.379 | 0.383 | 1.704 | 1.635 | 1.576 | 4.369 | 7.67E-07 | 0.004151717 | Up |
| Q91WF7 | Polyphosphoinositide phosphatase OS=Mus musculus OX=10090 GN=Fig4 PE=1 SV=1 | Fig4 | 0.397 | 0.356 | 0.372 | 1.653 | 1.66 | 1.599 | 4.366 | 1.65E-06 | 0.00871571 | Up |
| Q8JZV7 | N-acetylglucosamine-6-phosphate deacetylase OS=Mus musculus OX=10090 GN=Amdhd2 PE=1 SV=1 | Amdhd2 | 0.4 | 0.391 | 0.348 | 1.668 | 1.641 | 1.629 | 4.335 | 4.67E-06 | 0.023406641 | Up |
| Q9Z2M7 | Phosphomannomutase 2 OS=Mus musculus OX=10090 GN=Pmm2 PE=1 SV=1 | Pmm2 | 0.362 | 0.407 | 0.387 | 1.64 | 1.709 | 1.647 | 4.322 | 2.27E-06 | 0.011861433 | Up |
| Q8CIG8 | Protein arginine N-methyltransferase 5 OS=Mus musculus OX=10090 GN=Prmt5 PE=1 SV=3 | Prmt5 | 0.372 | 0.403 | 0.353 | 1.595 | 1.625 | 1.654 | 4.321 | 3.29E-06 | 0.016867529 | Up |
| Q8R050 | Eukaryotic peptide chain release factor GTP-binding subunit ERF3A OS=Mus musculus OX=10090 GN=Gspt1 PE=1 SV=2 | Gspt1 | 0.366 | 0.408 | 0.349 | 1.68 | 1.566 | 1.604 | 4.319 | 8.49E-06 | 0.040594781 | Up |
| Q8VDD5 | Myosin-9 OS=Mus musculus OX=10090 GN=Myh9 PE=1 SV=4 | Myh9 | 0.388 | 0.377 | 0.36 | 1.62 | 1.629 | 1.608 | 4.317 | 3.14E-07 | 0.00173361 | Up |
| O08788 | Dynactin subunit 1 OS=Mus musculus OX=10090 GN=Dctn1 PE=1 SV=3 | Dctn1 | 0.385 | 0.381 | 0.37 | 1.636 | 1.608 | 1.637 | 4.297 | 4.10E-08 | 0.000229437 | Up |
| Q62393 | Tumor protein D52 OS=Mus musculus OX=10090 GN=Tpd52 PE=1 SV=2 | Tpd52 | 0.379 | 0.35 | 0.397 | 1.669 | 1.537 | 1.629 | 4.294 | 5.03E-06 | 0.025045397 | Up |
| P97372 | Proteasome activator complex subunit 2 OS=Mus musculus OX=10090 GN=Psme2 PE=1 SV=4 | Psme2 | 0.379 | 0.382 | 0.368 | 1.66 | 1.522 | 1.652 | 4.282 | 1.14E-06 | 0.006095271 | Up |
| Q60710 | Deoxynucleoside triphosphate triphosphohydrolase SAMHD1 OS=Mus musculus OX=10090 GN=Samhd1 PE=1 SV=3 | Samhd1 | 0.374 | 0.365 | 0.383 | 1.608 | 1.589 | 1.59 | 4.266 | 5.85E-08 | 0.000326949 | Up |
| Q02053 | Ubiquitin-like modifier-activating enzyme 1 OS=Mus musculus OX=10090 GN=Uba1 PE=1 SV=1 | Uba1 | 0.39 | 0.368 | 0.385 | 1.604 | 1.642 | 1.623 | 4.26 | 1.72E-07 | 0.000953189 | Up |
| P97494 | Glutamate--cysteine ligase catalytic subunit OS=Mus musculus OX=10090 GN=Gclc PE=1 SV=4 | Gclc | 0.357 | 0.382 | 0.41 | 1.684 | 1.606 | 1.59 | 4.247 | 4.96E-06 | 0.024736789 | Up |
| P23492 | Purine nucleoside phosphorylase OS=Mus musculus OX=10090 GN=Pnp PE=1 SV=2 | Pnp | 0.354 | 0.398 | 0.392 | 1.668 | 1.642 | 1.542 | 4.241 | 5.08E-06 | 0.025289984 | Up |
| P29391 | Ferritin light chain 1 OS=Mus musculus OX=10090 GN=Ftl1 PE=1 SV=2 | Ftl1 | 0.362 | 0.402 | 0.398 | 1.676 | 1.526 | 1.726 | 4.241 | 8.50E-06 | 0.040652801 | Up |
| P46664 | Adenylosuccinate synthetase isozyme 2 OS=Mus musculus OX=10090 GN=Adss2 PE=1 SV=2 | Adss2 | 0.393 | 0.36 | 0.396 | 1.602 | 1.684 | 1.576 | 4.232 | 2.45E-06 | 0.012729087 | Up |
| P05201 | Aspartate aminotransferase, cytoplasmic OS=Mus musculus OX=10090 GN=Got1 PE=1 SV=3 | Got1 | 0.372 | 0.38 | 0.366 | 1.512 | 1.65 | 1.562 | 4.225 | 8.19E-07 | 0.004426946 | Up |
| Q8CGA0 | Protein phosphatase 1F OS=Mus musculus OX=10090 GN=Ppm1f PE=1 SV=1 | Ppm1f | 0.358 | 0.422 | 0.378 | 1.677 | 1.599 | 1.605 | 4.215 | 9.15E-06 | 0.043454503 | Up |
| Q9ESY9 | Gamma-interferon-inducible lysosomal thiol reductase OS=Mus musculus OX=10090 GN=Ifi30 PE=1 SV=3 | Ifi30 | 0.388 | 0.396 | 0.378 | 1.756 | 1.574 | 1.546 | 4.196 | 4.41E-06 | 0.022178984 | Up |
| Q9WV80 | Sorting nexin-1 OS=Mus musculus OX=10090 GN=Snx1 PE=1 SV=1 | Snx1 | 0.392 | 0.381 | 0.378 | 1.707 | 1.549 | 1.566 | 4.189 | 1.62E-06 | 0.008547741 | Up |
| Q9D358 | Low molecular weight phosphotyrosine protein phosphatase OS=Mus musculus OX=10090 GN=Acp1 PE=1 SV=3 | Acp1 | 0.402 | 0.389 | 0.37 | 1.622 | 1.616 | 1.618 | 4.183 | 4.84E-07 | 0.002655746 | Up |
| Q61598 | Rab GDP dissociation inhibitor beta OS=Mus musculus OX=10090 GN=Gdi2 PE=1 SV=1 | Gdi2 | 0.378 | 0.408 | 0.354 | 1.53 | 1.643 | 1.576 | 4.166 | 6.38E-06 | 0.031195697 | Up |
| Q920A5 | Retinoid-inducible serine carboxypeptidase OS=Mus musculus OX=10090 GN=Scpep1 PE=1 SV=2 | Scpep1 | 0.418 | 0.385 | 0.376 | 1.662 | 1.601 | 1.644 | 4.162 | 1.92E-06 | 0.010091246 | Up |
| Q60676 | Serine/threonine-protein phosphatase 5 OS=Mus musculus OX=10090 GN=Ppp5c PE=1 SV=3 | Ppp5c | 0.408 | 0.392 | 0.382 | 1.668 | 1.658 | 1.586 | 4.156 | 5.58E-07 | 0.003046028 | Up |
| P05064 | Fructose-bisphosphate aldolase A OS=Mus musculus OX=10090 GN=Aldoa PE=1 SV=2 | Aldoa | 0.385 | 0.4 | 0.365 | 1.56 | 1.606 | 1.608 | 4.151 | 9.39E-07 | 0.005056988 | Up |
| P80315 | T-complex protein 1 subunit delta OS=Mus musculus OX=10090 GN=Cct4 PE=1 SV=3 | Cct4 | 0.398 | 0.376 | 0.389 | 1.619 | 1.629 | 1.571 | 4.144 | 2.33E-07 | 0.001289928 | Up |
| P06745 | Glucose-6-phosphate isomerase OS=Mus musculus OX=10090 GN=Gpi PE=1 SV=4 | Gpi | 0.388 | 0.395 | 0.375 | 1.554 | 1.603 | 1.638 | 4.141 | 3.19E-07 | 0.001758929 | Up |
| Q9QUI0 | Transforming protein RhoA OS=Mus musculus OX=10090 GN=Rhoa PE=1 SV=1 | Rhoa | 0.38 | 0.38 | 0.401 | 1.584 | 1.639 | 1.534 | 4.097 | 7.14E-07 | 0.003872956 | Up |
| P68134 | Actin, alpha skeletal muscle OS=Mus musculus OX=10090 GN=Acta1 PE=1 SV=1 | Acta1 | 0.406 | 0.388 | 0.398 | 1.627 | 1.598 | 1.632 | 4.075 | 7.14E-08 | 0.000398216 | Up |
| P24547 | Inosine-5'-monophosphate dehydrogenase 2 OS=Mus musculus OX=10090 GN=Impdh2 PE=1 SV=2 | Impdh2 | 0.396 | 0.417 | 0.371 | 1.616 | 1.608 | 1.588 | 4.064 | 2.11E-06 | 0.01106536 | Up |
| Q9DBT5 | AMP deaminase 2 OS=Mus musculus OX=10090 GN=Ampd2 PE=1 SV=1 | Ampd2 | 0.422 | 0.38 | 0.401 | 1.671 | 1.582 | 1.618 | 4.049 | 2.13E-06 | 0.011147688 | Up |
| P21300 | Aldo-keto reductase family 1 member B7 OS=Mus musculus OX=10090 GN=Akr1b7 PE=1 SV=4 | Akr1b7 | 0.384 | 0.414 | 0.4 | 1.608 | 1.64 | 1.586 | 4.035 | 5.08E-07 | 0.002780961 | Up |
| P48722 | Heat shock 70 kDa protein 4L OS=Mus musculus OX=10090 GN=Hspa4l PE=1 SV=2 | Hspa4l | 0.427 | 0.391 | 0.382 | 1.59 | 1.656 | 1.579 | 4.021 | 2.98E-06 | 0.015378046 | Up |
| Q9EST4 | Proteasome assembly chaperone 2 OS=Mus musculus OX=10090 GN=Psmg2 PE=1 SV=1 | Psmg2 | 0.419 | 0.381 | 0.395 | 1.666 | 1.542 | 1.591 | 4.016 | 2.59E-06 | 0.01342544 | Up |
| O88545 | COP9 signalosome complex subunit 6 OS=Mus musculus OX=10090 GN=Cops6 PE=1 SV=1 | Cops6 | 0.386 | 0.425 | 0.383 | 1.546 | 1.645 | 1.579 | 3.995 | 3.41E-06 | 0.01744007 | Up |
| Q8BL66 | Early endosome antigen 1 OS=Mus musculus OX=10090 GN=Eea1 PE=1 SV=2 | Eea1 | 0.37 | 0.42 | 0.411 | 1.671 | 1.53 | 1.579 | 3.98 | 7.88E-06 | 0.037860563 | Up |
| P80317 | T-complex protein 1 subunit zeta OS=Mus musculus OX=10090 GN=Cct6a PE=1 SV=3 | Cct6a | 0.421 | 0.419 | 0.39 | 1.616 | 1.692 | 1.588 | 3.98 | 1.54E-06 | 0.008181822 | Up |
| Q64337 | Sequestosome-1 OS=Mus musculus OX=10090 GN=Sqstm1 PE=1 SV=1 | Sqstm1 | 0.372 | 0.421 | 0.41 | 1.681 | 1.592 | 1.503 | 3.97 | 9.95E-06 | 0.04678335 | Up |
| P42932 | T-complex protein 1 subunit theta OS=Mus musculus OX=10090 GN=Cct8 PE=1 SV=3 | Cct8 | 0.388 | 0.4 | 0.409 | 1.574 | 1.6 | 1.569 | 3.962 | 1.22E-07 | 0.000676993 | Up |
| Q9JHU9 | Inositol-3-phosphate synthase 1 OS=Mus musculus OX=10090 GN=Isyna1 PE=1 SV=1 | Isyna1 | 0.412 | 0.391 | 0.394 | 1.552 | 1.662 | 1.523 | 3.957 | 1.57E-06 | 0.008333127 | Up |
| P80316 | T-complex protein 1 subunit epsilon OS=Mus musculus OX=10090 GN=Cct5 PE=1 SV=1 | Cct5 | 0.397 | 0.414 | 0.396 | 1.528 | 1.607 | 1.62 | 3.94 | 4.99E-07 | 0.002733062 | Up |
| P45377 | Aldose reductase-related protein 2 OS=Mus musculus OX=10090 GN=Akr1b8 PE=1 SV=2 | Akr1b8 | 0.396 | 0.428 | 0.396 | 1.602 | 1.679 | 1.523 | 3.938 | 3.63E-06 | 0.018453337 | Up |
| P32921 | Tryptophan--tRNA ligase, cytoplasmic OS=Mus musculus OX=10090 GN=Wars1 PE=1 SV=2 | Wars1 | 0.42 | 0.394 | 0.415 | 1.596 | 1.635 | 1.597 | 3.928 | 3.42E-07 | 0.001886922 | Up |
| P52480 | Pyruvate kinase PKM OS=Mus musculus OX=10090 GN=Pkm PE=1 SV=4 | Pkm | 0.416 | 0.406 | 0.377 | 1.522 | 1.651 | 1.531 | 3.923 | 4.17E-06 | 0.021022669 | Up |
| Q9D2V7 | Coronin-7 OS=Mus musculus OX=10090 GN=Coro7 PE=1 SV=2 | Coro7 | 0.416 | 0.41 | 0.401 | 1.592 | 1.603 | 1.605 | 3.912 | 2.51E-08 | 0.000140524 | Up |
| P21550 | Beta-enolase OS=Mus musculus OX=10090 GN=Eno3 PE=1 SV=3 | Eno3 | 0.408 | 0.406 | 0.408 | 1.586 | 1.6 | 1.57 | 3.892 | 1.87E-09 | 1.05E-05 | Up |
| Q9R1T4 | Septin-6 OS=Mus musculus OX=10090 GN=Septin6 PE=1 SV=4 | Septin6 | 0.418 | 0.414 | 0.401 | 1.534 | 1.642 | 1.618 | 3.888 | 6.02E-07 | 0.003280878 | Up |
| Q8VD04 | GRIP1-associated protein 1 OS=Mus musculus OX=10090 GN=Gripap1 PE=1 SV=1 | Gripap1 | 0.401 | 0.42 | 0.412 | 1.706 | 1.585 | 1.486 | 3.874 | 5.58E-06 | 0.027601845 | Up |
| Q9D2C2 | Protein SAAL1 OS=Mus musculus OX=10090 GN=Saal1 PE=1 SV=1 | Saal1 | 0.429 | 0.415 | 0.377 | 1.549 | 1.586 | 1.593 | 3.872 | 4.40E-06 | 0.022108691 | Up |
| Q99MN1 | Lysine--tRNA ligase OS=Mus musculus OX=10090 GN=Kars1 PE=1 SV=1 | Kars1 | 0.422 | 0.424 | 0.386 | 1.648 | 1.55 | 1.572 | 3.872 | 2.89E-06 | 0.014923594 | Up |
| Q9R0P3 | S-formylglutathione hydrolase OS=Mus musculus OX=10090 GN=Esd PE=1 SV=1 | Esd | 0.393 | 0.423 | 0.388 | 1.489 | 1.629 | 1.522 | 3.854 | 3.82E-06 | 0.019375157 | Up |
| Q61699 | Heat shock protein 105 kDa OS=Mus musculus OX=10090 GN=Hsph1 PE=1 SV=2 | Hsph1 | 0.434 | 0.403 | 0.404 | 1.569 | 1.661 | 1.542 | 3.845 | 2.18E-06 | 0.011382216 | Up |
| P16045 | Galectin-1 OS=Mus musculus OX=10090 GN=Lgals1 PE=1 SV=3 | Lgals1 | 0.401 | 0.424 | 0.429 | 1.699 | 1.588 | 1.53 | 3.841 | 3.44E-06 | 0.017583118 | Up |
| Q68FD5 | Clathrin heavy chain 1 OS=Mus musculus OX=10090 GN=Cltc PE=1 SV=3 | Cltc | 0.402 | 0.434 | 0.405 | 1.61 | 1.612 | 1.538 | 3.836 | 1.27E-06 | 0.006787276 | Up |
| Q9CWF2 | Tubulin beta-2B chain OS=Mus musculus OX=10090 GN=Tubb2b PE=1 SV=1 | Tubb2b | 0.414 | 0.449 | 0.387 | 1.634 | 1.525 | 1.636 | 3.836 | 1.03E-05 | 0.048324845 | Up |
| Q7TNG5 | Echinoderm microtubule-associated protein-like 2 OS=Mus musculus OX=10090 GN=Eml2 PE=1 SV=1 | Eml2 | 0.427 | 0.405 | 0.417 | 1.639 | 1.598 | 1.516 | 3.805 | 1.09E-06 | 0.005841167 | Up |
| O88487 | Cytoplasmic dynein 1 intermediate chain 2 OS=Mus musculus OX=10090 GN=Dync1i2 PE=1 SV=1 | Dync1i2 | 0.4 | 0.436 | 0.411 | 1.646 | 1.578 | 1.503 | 3.791 | 3.38E-06 | 0.01730973 | Up |
| P99024 | Tubulin beta-5 chain OS=Mus musculus OX=10090 GN=Tubb5 PE=1 SV=1 | Tubb5 | 0.43 | 0.402 | 0.429 | 1.611 | 1.613 | 1.556 | 3.791 | 7.43E-07 | 0.00402777 | Up |
| Q9ERL7 | Glia maturation factor gamma OS=Mus musculus OX=10090 GN=Gmfg PE=1 SV=1 | Gmfg | 0.422 | 0.403 | 0.437 | 1.643 | 1.615 | 1.516 | 3.783 | 2.51E-06 | 0.013067109 | Up |
| Q571I9 | Aldehyde dehydrogenase family 16 member A1 OS=Mus musculus OX=10090 GN=Aldh16a1 PE=1 SV=2 | Aldh16a1 | 0.379 | 0.406 | 0.436 | 1.507 | 1.573 | 1.538 | 3.782 | 6.07E-06 | 0.029773576 | Up |
| Q91ZJ5 | UTP--glucose-1-phosphate uridylyltransferase OS=Mus musculus OX=10090 GN=Ugp2 PE=1 SV=3 | Ugp2 | 0.404 | 0.418 | 0.43 | 1.548 | 1.632 | 1.555 | 3.782 | 7.14E-07 | 0.003872956 | Up |
| Q64737 | Trifunctional purine biosynthetic protein adenosine-3 OS=Mus musculus OX=10090 GN=Gart PE=1 SV=3 | Gart | 0.446 | 0.381 | 0.425 | 1.576 | 1.588 | 1.544 | 3.76 | 9.65E-06 | 0.045533909 | Up |
| Q9Z0S1 | 3'(2'),5'-bisphosphate nucleotidase 1 OS=Mus musculus OX=10090 GN=Bpnt1 PE=1 SV=2 | Bpnt1 | 0.406 | 0.434 | 0.428 | 1.579 | 1.598 | 1.59 | 3.759 | 3.51E-07 | 0.00193176 | Up |
| Q8BJY1 | 26S proteasome non-ATPase regulatory subunit 5 OS=Mus musculus OX=10090 GN=Psmd5 PE=1 SV=4 | Psmd5 | 0.412 | 0.418 | 0.435 | 1.578 | 1.599 | 1.575 | 3.757 | 1.60E-07 | 0.000890264 | Up |
| P13020 | Gelsolin OS=Mus musculus OX=10090 GN=Gsn PE=1 SV=3 | Gsn | 0.4 | 0.427 | 0.425 | 1.54 | 1.613 | 1.55 | 3.756 | 8.29E-07 | 0.004480246 | Up |
| Q11011 | Puromycin-sensitive aminopeptidase OS=Mus musculus OX=10090 GN=Npepps PE=1 SV=2 | Npepps | 0.426 | 0.45 | 0.4 | 1.618 | 1.6 | 1.558 | 3.743 | 3.23E-06 | 0.016565542 | Up |
| Q9Z1Q9 | Valine--tRNA ligase OS=Mus musculus OX=10090 GN=Vars1 PE=1 SV=1 | Vars1 | 0.417 | 0.456 | 0.41 | 1.623 | 1.607 | 1.557 | 3.731 | 3.07E-06 | 0.015792543 | Up |
| Q61768 | Kinesin-1 heavy chain OS=Mus musculus OX=10090 GN=Kif5b PE=1 SV=3 | Kif5b | 0.428 | 0.435 | 0.412 | 1.528 | 1.644 | 1.56 | 3.711 | 1.09E-06 | 0.00583718 | Up |
| Q6ZQ38 | Cullin-associated NEDD8-dissociated protein 1 OS=Mus musculus OX=10090 GN=Cand1 PE=1 SV=2 | Cand1 | 0.428 | 0.418 | 0.425 | 1.56 | 1.595 | 1.561 | 3.71 | 2.12E-08 | 0.00011891 | Up |
| Q9JMA1 | Ubiquitin carboxyl-terminal hydrolase 14 OS=Mus musculus OX=10090 GN=Usp14 PE=1 SV=3 | Usp14 | 0.437 | 0.418 | 0.427 | 1.568 | 1.632 | 1.556 | 3.71 | 2.98E-07 | 0.001648034 | Up |
| Q8BKC5 | Importin-5 OS=Mus musculus OX=10090 GN=Ipo5 PE=1 SV=3 | Ipo5 | 0.41 | 0.45 | 0.426 | 1.556 | 1.594 | 1.619 | 3.708 | 1.51E-06 | 0.007992916 | Up |
| Q9CZD3 | Glycine--tRNA ligase OS=Mus musculus OX=10090 GN=Gars1 PE=1 SV=1 | Gars1 | 0.452 | 0.405 | 0.416 | 1.574 | 1.635 | 1.501 | 3.7 | 5.90E-06 | 0.029041909 | Up |
| Q9JII6 | Aldo-keto reductase family 1 member A1 OS=Mus musculus OX=10090 GN=Akr1a1 PE=1 SV=3 | Akr1a1 | 0.444 | 0.392 | 0.434 | 1.531 | 1.613 | 1.553 | 3.698 | 5.93E-06 | 0.029158401 | Up |
| Q9CQH7 | Transcription factor BTF3 homolog 4 OS=Mus musculus OX=10090 GN=Btf3l4 PE=1 SV=1 | Btf3l4 | 0.446 | 0.423 | 0.415 | 1.615 | 1.496 | 1.63 | 3.692 | 2.97E-06 | 0.015323701 | Up |
| Q9JKF1 | Ras GTPase-activating-like protein IQGAP1 OS=Mus musculus OX=10090 GN=Iqgap1 PE=1 SV=2 | Iqgap1 | 0.438 | 0.434 | 0.416 | 1.618 | 1.565 | 1.544 | 3.67 | 4.16E-07 | 0.002283993 | Up |
| P80318 | T-complex protein 1 subunit gamma OS=Mus musculus OX=10090 GN=Cct3 PE=1 SV=1 | Cct3 | 0.447 | 0.427 | 0.419 | 1.56 | 1.531 | 1.624 | 3.647 | 9.62E-07 | 0.005180115 | Up |
| Q9JHR7 | Insulin-degrading enzyme OS=Mus musculus OX=10090 GN=Ide PE=1 SV=1 | Ide | 0.422 | 0.449 | 0.449 | 1.593 | 1.66 | 1.552 | 3.64 | 1.41E-06 | 0.007504418 | Up |
| P54728 | UV excision repair protein RAD23 homolog B OS=Mus musculus OX=10090 GN=Rad23b PE=1 SV=2 | Rad23b | 0.433 | 0.41 | 0.414 | 1.538 | 1.514 | 1.519 | 3.636 | 2.01E-07 | 0.001116553 | Up |
| P63260 | Actin, cytoplasmic 2 OS=Mus musculus OX=10090 GN=Actg1 PE=1 SV=1 | Actg1 | 0.416 | 0.454 | 0.396 | 1.492 | 1.53 | 1.55 | 3.611 | 6.47E-06 | 0.031627225 | Up |
| Q5ND34 | WD repeat-containing protein 81 OS=Mus musculus OX=10090 GN=Wdr81 PE=1 SV=2 | Wdr81 | 0.427 | 0.412 | 0.471 | 1.558 | 1.53 | 1.642 | 3.611 | 9.16E-06 | 0.043499087 | Up |
| P68254 | 14-3-3 protein theta OS=Mus musculus OX=10090 GN=Ywhaq PE=1 SV=1 | Ywhaq | 0.433 | 0.437 | 0.442 | 1.478 | 1.531 | 1.705 | 3.593 | 7.93E-06 | 0.038093896 | Up |
| Q8VCT3 | Aminopeptidase B OS=Mus musculus OX=10090 GN=Rnpep PE=1 SV=2 | Rnpep | 0.422 | 0.465 | 0.417 | 1.584 | 1.484 | 1.603 | 3.582 | 6.99E-06 | 0.03395125 | Up |
| Q0VGB7 | Serine/threonine-protein phosphatase 4 regulatory subunit 2 OS=Mus musculus OX=10090 GN=Ppp4r2 PE=1 SV=1 | Ppp4r2 | 0.436 | 0.416 | 0.466 | 1.534 | 1.58 | 1.57 | 3.554 | 3.12E-06 | 0.016043272 | Up |
| P29477 | Nitric oxide synthase, inducible OS=Mus musculus OX=10090 GN=Nos2 PE=1 SV=1 | Nos2 | 0.429 | 0.449 | 0.421 | 1.553 | 1.552 | 1.509 | 3.552 | 4.80E-07 | 0.002633647 | Up |
| P68037 | Ubiquitin-conjugating enzyme E2 L3 OS=Mus musculus OX=10090 GN=Ube2l3 PE=1 SV=1 | Ube2l3 | 0.446 | 0.422 | 0.447 | 1.628 | 1.549 | 1.494 | 3.552 | 2.20E-06 | 0.011501555 | Up |
| Q99PL6 | UBX domain-containing protein 6 OS=Mus musculus OX=10090 GN=Ubxn6 PE=1 SV=1 | Ubxn6 | 0.458 | 0.432 | 0.434 | 1.445 | 1.67 | 1.58 | 3.546 | 1.05E-05 | 0.049271379 | Up |
| Q9JLQ0 | CD2-associated protein OS=Mus musculus OX=10090 GN=Cd2ap PE=1 SV=3 | Cd2ap | 0.462 | 0.421 | 0.436 | 1.515 | 1.604 | 1.546 | 3.537 | 2.41E-06 | 0.012531114 | Up |
| Q61151 | Serine/threonine-protein phosphatase 2A 56 kDa regulatory subunit epsilon isoform OS=Mus musculus OX=10090 GN=Ppp2r5e PE=1 SV=3 | Ppp2r5e | 0.417 | 0.48 | 0.439 | 1.618 | 1.526 | 1.56 | 3.521 | 9.26E-06 | 0.043893301 | Up |
| Q8R5H1 | Ubiquitin carboxyl-terminal hydrolase 15 OS=Mus musculus OX=10090 GN=Usp15 PE=1 SV=1 | Usp15 | 0.466 | 0.42 | 0.414 | 1.538 | 1.566 | 1.466 | 3.515 | 7.50E-06 | 0.036203927 | Up |
| Q91YE6 | Importin-9 OS=Mus musculus OX=10090 GN=Ipo9 PE=1 SV=3 | Ipo9 | 0.439 | 0.435 | 0.444 | 1.527 | 1.53 | 1.567 | 3.508 | 2.62E-08 | 0.000146701 | Up |
| P53810 | Phosphatidylinositol transfer protein alpha isoform OS=Mus musculus OX=10090 GN=Pitpna PE=1 SV=2 | Pitpna | 0.472 | 0.427 | 0.422 | 1.499 | 1.598 | 1.529 | 3.502 | 6.31E-06 | 0.030920931 | Up |
| P61161 | Actin-related protein 2 OS=Mus musculus OX=10090 GN=Actr2 PE=1 SV=1 | Actr2 | 0.448 | 0.45 | 0.456 | 1.61 | 1.542 | 1.547 | 3.47 | 1.22E-07 | 0.000678769 | Up |
| O08529 | Calpain-2 catalytic subunit OS=Mus musculus OX=10090 GN=Capn2 PE=1 SV=4 | Capn2 | 0.465 | 0.46 | 0.42 | 1.544 | 1.507 | 1.615 | 3.469 | 5.25E-06 | 0.026051256 | Up |
| Q91WQ3 | Tyrosine--tRNA ligase, cytoplasmic OS=Mus musculus OX=10090 GN=Yars1 PE=1 SV=3 | Yars1 | 0.458 | 0.465 | 0.42 | 1.512 | 1.646 | 1.497 | 3.466 | 9.10E-06 | 0.04324362 | Up |
| Q68FL6 | Methionine--tRNA ligase, cytoplasmic OS=Mus musculus OX=10090 GN=Mars1 PE=1 SV=1 | Mars1 | 0.463 | 0.45 | 0.442 | 1.58 | 1.586 | 1.518 | 3.457 | 3.64E-07 | 0.002002454 | Up |
| P80313 | T-complex protein 1 subunit eta OS=Mus musculus OX=10090 GN=Cct7 PE=1 SV=1 | Cct7 | 0.417 | 0.469 | 0.446 | 1.523 | 1.6 | 1.476 | 3.453 | 7.37E-06 | 0.035638858 | Up |
| P19096 | Fatty acid synthase OS=Mus musculus OX=10090 GN=Fasn PE=1 SV=2 | Fasn | 0.448 | 0.446 | 0.444 | 1.573 | 1.536 | 1.503 | 3.447 | 8.24E-08 | 0.000459758 | Up |
| Q9Z1K5 | E3 ubiquitin-protein ligase ARIH1 OS=Mus musculus OX=10090 GN=Arih1 PE=1 SV=3 | Arih1 | 0.453 | 0.458 | 0.446 | 1.564 | 1.508 | 1.602 | 3.444 | 3.48E-07 | 0.001915799 | Up |
| P24452 | Macrophage-capping protein OS=Mus musculus OX=10090 GN=Capg PE=1 SV=2 | Capg | 0.43 | 0.436 | 0.448 | 1.545 | 1.457 | 1.521 | 3.442 | 5.25E-07 | 0.002871925 | Up |
| Q99LI8 | Hepatocyte growth factor-regulated tyrosine kinase substrate OS=Mus musculus OX=10090 GN=Hgs PE=1 SV=2 | Hgs | 0.451 | 0.468 | 0.439 | 1.566 | 1.577 | 1.528 | 3.44 | 4.87E-07 | 0.002668481 | Up |
| Q9CR00 | 26S proteasome non-ATPase regulatory subunit 9 OS=Mus musculus OX=10090 GN=Psmd9 PE=1 SV=1 | Psmd9 | 0.466 | 0.424 | 0.472 | 1.638 | 1.531 | 1.514 | 3.438 | 7.81E-06 | 0.03758334 | Up |
| Q61024 | Asparagine synthetase [glutamine-hydrolyzing] OS=Mus musculus OX=10090 GN=Asns PE=1 SV=3 | Asns | 0.47 | 0.436 | 0.469 | 1.566 | 1.577 | 1.566 | 3.425 | 9.83E-07 | 0.005285416 | Up |
| Q8BMJ2 | Leucine--tRNA ligase, cytoplasmic OS=Mus musculus OX=10090 GN=Lars1 PE=1 SV=2 | Lars1 | 0.434 | 0.457 | 0.466 | 1.56 | 1.509 | 1.578 | 3.424 | 1.03E-06 | 0.005554097 | Up |
| Q9D0B6 | Protein PBDC1 OS=Mus musculus OX=10090 GN=Pbdc1 PE=1 SV=1 | Pbdc1 | 0.429 | 0.486 | 0.438 | 1.59 | 1.532 | 1.509 | 3.423 | 7.75E-06 | 0.03731798 | Up |
| Q62048 | Astrocytic phosphoprotein PEA-15 OS=Mus musculus OX=10090 GN=Pea15 PE=1 SV=1 | Pea15 | 0.43 | 0.463 | 0.476 | 1.585 | 1.494 | 1.591 | 3.411 | 4.68E-06 | 0.02344017 | Up |
| Q505F5 | Leucine-rich repeat-containing protein 47 OS=Mus musculus OX=10090 GN=Lrrc47 PE=1 SV=1 | Lrrc47 | 0.433 | 0.47 | 0.462 | 1.615 | 1.588 | 1.442 | 3.403 | 9.26E-06 | 0.043893301 | Up |
| Q9R1P4 | Proteasome subunit alpha type-1 OS=Mus musculus OX=10090 GN=Psma1 PE=1 SV=1 | Psma1 | 0.455 | 0.44 | 0.457 | 1.529 | 1.6 | 1.468 | 3.4 | 1.55E-06 | 0.008209317 | Up |
| O88456 | Calpain small subunit 1 OS=Mus musculus OX=10090 GN=Capns1 PE=1 SV=1 | Capns1 | 0.483 | 0.434 | 0.456 | 1.521 | 1.645 | 1.494 | 3.394 | 8.93E-06 | 0.042483183 | Up |
| Q80TY0 | Formin-binding protein 1 OS=Mus musculus OX=10090 GN=Fnbp1 PE=1 SV=2 | Fnbp1 | 0.419 | 0.485 | 0.454 | 1.538 | 1.516 | 1.523 | 3.37 | 8.86E-06 | 0.042193029 | Up |
| P68368 | Tubulin alpha-4A chain OS=Mus musculus OX=10090 GN=Tuba4a PE=1 SV=1 | Tuba4a | 0.446 | 0.464 | 0.448 | 1.516 | 1.587 | 1.438 | 3.344 | 2.64E-06 | 0.013699023 | Up |
| O09131 | Glutathione S-transferase omega-1 OS=Mus musculus OX=10090 GN=Gsto1 PE=1 SV=2 | Gsto1 | 0.462 | 0.467 | 0.454 | 1.586 | 1.501 | 1.529 | 3.338 | 3.09E-07 | 0.00170484 | Up |
| Q3UW53 | Protein Niban 1 OS=Mus musculus OX=10090 GN=Niban1 PE=1 SV=2 | Niban1 | 0.442 | 0.47 | 0.483 | 1.588 | 1.547 | 1.521 | 3.338 | 2.02E-06 | 0.010615024 | Up |
| Q8C1Z8 | tRNA methyltransferase 10 homolog A OS=Mus musculus OX=10090 GN=Trmt10a PE=2 SV=2 | Trmt10a | 0.456 | 0.477 | 0.472 | 1.58 | 1.568 | 1.538 | 3.335 | 1.77E-07 | 0.000980921 | Up |
| O55091 | Protein IMPACT OS=Mus musculus OX=10090 GN=Impact PE=1 SV=2 | Impact | 0.458 | 0.488 | 0.45 | 1.605 | 1.544 | 1.493 | 3.325 | 3.11E-06 | 0.016000482 | Up |
| Q9JKV1 | Proteasomal ubiquitin receptor ADRM1 OS=Mus musculus OX=10090 GN=Adrm1 PE=1 SV=2 | Adrm1 | 0.436 | 0.453 | 0.454 | 1.483 | 1.501 | 1.476 | 3.321 | 1.13E-07 | 0.00062734 | Up |
| Q04447 | Creatine kinase B-type OS=Mus musculus OX=10090 GN=Ckb PE=1 SV=1 | Ckb | 0.498 | 0.456 | 0.458 | 1.58 | 1.6 | 1.496 | 3.312 | 4.51E-06 | 0.022618894 | Up |
| Q922B2 | Aspartate--tRNA ligase, cytoplasmic OS=Mus musculus OX=10090 GN=Dars1 PE=1 SV=2 | Dars1 | 0.467 | 0.46 | 0.466 | 1.54 | 1.536 | 1.528 | 3.305 | 2.23E-09 | 1.25E-05 | Up |
| O54988 | STE20-like serine/threonine-protein kinase OS=Mus musculus OX=10090 GN=Slk PE=1 SV=2 | Slk | 0.47 | 0.485 | 0.448 | 1.614 | 1.541 | 1.473 | 3.299 | 4.44E-06 | 0.022310157 | Up |
| Q99KQ4 | Nicotinamide phosphoribosyltransferase OS=Mus musculus OX=10090 GN=Nampt PE=1 SV=1 | Nampt | 0.467 | 0.443 | 0.48 | 1.508 | 1.546 | 1.523 | 3.293 | 1.09E-06 | 0.00583718 | Up |
| Q3UGC7 | Eukaryotic translation initiation factor 3 subunit J-A OS=Mus musculus OX=10090 GN=Eif3j1 PE=2 SV=1 | Eif3j1 | 0.478 | 0.448 | 0.467 | 1.581 | 1.536 | 1.46 | 3.286 | 2.42E-06 | 0.012610333 | Up |
| P48453 | Serine/threonine-protein phosphatase 2B catalytic subunit beta isoform OS=Mus musculus OX=10090 GN=Ppp3cb PE=1 SV=2 | Ppp3cb | 0.476 | 0.491 | 0.446 | 1.58 | 1.52 | 1.527 | 3.275 | 2.72E-06 | 0.014103254 | Up |
| Q3UQ44 | Ras GTPase-activating-like protein IQGAP2 OS=Mus musculus OX=10090 GN=Iqgap2 PE=1 SV=2 | Iqgap2 | 0.49 | 0.486 | 0.436 | 1.532 | 1.546 | 1.545 | 3.274 | 6.08E-06 | 0.029858652 | Up |
| Q8CE96 | tRNA (adenine(58)-N(1))-methyltransferase non-catalytic subunit TRM6 OS=Mus musculus OX=10090 GN=Trmt6 PE=1 SV=1 | Trmt6 | 0.464 | 0.486 | 0.445 | 1.564 | 1.581 | 1.416 | 3.27 | 1.07E-05 | 0.049807624 | Up |
| Q99KB8 | Hydroxyacylglutathione hydrolase, mitochondrial OS=Mus musculus OX=10090 GN=Hagh PE=1 SV=2 | Hagh | 0.449 | 0.487 | 0.462 | 1.495 | 1.55 | 1.521 | 3.266 | 1.39E-06 | 0.007382578 | Up |
| P54822 | Adenylosuccinate lyase OS=Mus musculus OX=10090 GN=Adsl PE=1 SV=2 | Adsl | 0.472 | 0.456 | 0.46 | 1.519 | 1.429 | 1.58 | 3.262 | 2.83E-06 | 0.014625324 | Up |
| Q9CWK8 | Sorting nexin-2 OS=Mus musculus OX=10090 GN=Snx2 PE=1 SV=2 | Snx2 | 0.472 | 0.464 | 0.447 | 1.543 | 1.485 | 1.484 | 3.262 | 5.51E-07 | 0.003007974 | Up |
| P13597 | Intercellular adhesion molecule 1 OS=Mus musculus OX=10090 GN=Icam1 PE=1 SV=1 | Icam1 | 0.475 | 0.481 | 0.465 | 1.665 | 1.476 | 1.487 | 3.257 | 8.05E-06 | 0.038619352 | Up |
| P40336 | Vacuolar protein sorting-associated protein 26A OS=Mus musculus OX=10090 GN=Vps26a PE=1 SV=1 | Vps26a | 0.5 | 0.468 | 0.471 | 1.602 | 1.55 | 1.515 | 3.243 | 1.56E-06 | 0.008242258 | Up |
| O35643 | AP-1 complex subunit beta-1 OS=Mus musculus OX=10090 GN=Ap1b1 PE=1 SV=2 | Ap1b1 | 0.514 | 0.451 | 0.466 | 1.581 | 1.562 | 1.494 | 3.24 | 1.05E-05 | 0.04905725 | Up |
| Q3THS6 | S-adenosylmethionine synthase isoform type-2 OS=Mus musculus OX=10090 GN=Mat2a PE=1 SV=2 | Mat2a | 0.479 | 0.474 | 0.466 | 1.531 | 1.528 | 1.538 | 3.24 | 1.45E-08 | 8.14E-05 | Up |
| Q148V7 | RAB11-binding protein RELCH OS=Mus musculus OX=10090 GN=Relch PE=1 SV=1 | Relch | 0.441 | 0.495 | 0.469 | 1.57 | 1.502 | 1.479 | 3.239 | 6.40E-06 | 0.031306856 | Up |
| Q8R3C0 | Mini-chromosome maintenance complex-binding protein OS=Mus musculus OX=10090 GN=Mcmbp PE=1 SV=1 | Mcmbp | 0.454 | 0.498 | 0.472 | 1.541 | 1.603 | 1.457 | 3.231 | 6.96E-06 | 0.033857574 | Up |
| Q8BYA0 | Tubulin-specific chaperone D OS=Mus musculus OX=10090 GN=Tbcd PE=1 SV=1 | Tbcd | 0.472 | 0.468 | 0.471 | 1.527 | 1.48 | 1.547 | 3.227 | 1.02E-07 | 0.000569226 | Up |
| Q9QY36 | N-alpha-acetyltransferase 10 OS=Mus musculus OX=10090 GN=Naa10 PE=1 SV=1 | Naa10 | 0.492 | 0.462 | 0.476 | 1.62 | 1.516 | 1.464 | 3.217 | 4.71E-06 | 0.023575052 | Up |
| O88879 | Apoptotic protease-activating factor 1 OS=Mus musculus OX=10090 GN=Apaf1 PE=1 SV=3 | Apaf1 | 0.484 | 0.478 | 0.465 | 1.467 | 1.593 | 1.52 | 3.21 | 1.63E-06 | 0.008614436 | Up |
| Q9WVA4 | Transgelin-2 OS=Mus musculus OX=10090 GN=Tagln2 PE=1 SV=4 | Tagln2 | 0.488 | 0.473 | 0.498 | 1.659 | 1.476 | 1.492 | 3.171 | 8.79E-06 | 0.041855932 | Up |
| O88643 | Serine/threonine-protein kinase PAK 1 OS=Mus musculus OX=10090 GN=Pak1 PE=1 SV=1 | Pak1 | 0.475 | 0.445 | 0.509 | 1.538 | 1.522 | 1.464 | 3.166 | 1.01E-05 | 0.047196678 | Up |
| P22892 | AP-1 complex subunit gamma-1 OS=Mus musculus OX=10090 GN=Ap1g1 PE=1 SV=3 | Ap1g1 | 0.5 | 0.468 | 0.472 | 1.501 | 1.534 | 1.523 | 3.165 | 7.57E-07 | 0.004099247 | Up |
| Q9DBB8 | Trans-1,2-dihydrobenzene-1,2-diol dehydrogenase OS=Mus musculus OX=10090 GN=Dhdh PE=1 SV=1 | Dhdh | 0.491 | 0.472 | 0.462 | 1.501 | 1.465 | 1.54 | 3.162 | 9.41E-07 | 0.005069194 | Up |
| Q9CQ60 | 6-phosphogluconolactonase OS=Mus musculus OX=10090 GN=Pgls PE=1 SV=1 | Pgls | 0.502 | 0.46 | 0.468 | 1.542 | 1.518 | 1.449 | 3.153 | 3.87E-06 | 0.019611519 | Up |
| P25119 | Tumor necrosis factor receptor superfamily member 1B OS=Mus musculus OX=10090 GN=Tnfrsf1b PE=2 SV=1 | Tnfrsf1b | 0.488 | 0.451 | 0.515 | 1.532 | 1.512 | 1.54 | 3.153 | 7.82E-06 | 0.037601598 | Up |
| O88685 | 26S proteasome regulatory subunit 6A OS=Mus musculus OX=10090 GN=Psmc3 PE=1 SV=2 | Psmc3 | 0.505 | 0.486 | 0.46 | 1.6 | 1.481 | 1.491 | 3.151 | 6.21E-06 | 0.030424312 | Up |
| Q8BZW8 | NHL repeat-containing protein 2 OS=Mus musculus OX=10090 GN=Nhlrc2 PE=1 SV=1 | Nhlrc2 | 0.475 | 0.525 | 0.492 | 1.536 | 1.554 | 1.603 | 3.145 | 3.60E-06 | 0.018355783 | Up |
| Q91V92 | ATP-citrate synthase OS=Mus musculus OX=10090 GN=Acly PE=1 SV=1 | Acly | 0.483 | 0.497 | 0.476 | 1.567 | 1.505 | 1.494 | 3.136 | 5.09E-07 | 0.002784804 | Up |
| Q9D6N1 | Carbonic anhydrase 13 OS=Mus musculus OX=10090 GN=Ca13 PE=1 SV=1 | Ca13 | 0.507 | 0.487 | 0.463 | 1.466 | 1.613 | 1.487 | 3.134 | 8.69E-06 | 0.041450973 | Up |
| Q8VDM4 | 26S proteasome non-ATPase regulatory subunit 2 OS=Mus musculus OX=10090 GN=Psmd2 PE=1 SV=1 | Psmd2 | 0.498 | 0.474 | 0.481 | 1.58 | 1.494 | 1.478 | 3.133 | 1.45E-06 | 0.007717735 | Up |
| Q8CDN6 | Thioredoxin-like protein 1 OS=Mus musculus OX=10090 GN=Txnl1 PE=1 SV=3 | Txnl1 | 0.503 | 0.48 | 0.483 | 1.574 | 1.513 | 1.496 | 3.126 | 7.27E-07 | 0.003941317 | Up |
| A2AQ19 | RNA polymerase-associated protein RTF1 homolog OS=Mus musculus OX=10090 GN=Rtf1 PE=1 SV=1 | Rtf1 | 0.465 | 0.49 | 0.501 | 1.517 | 1.59 | 1.425 | 3.113 | 8.03E-06 | 0.038515753 | Up |
| Q9Z2Y8 | Pyridoxal phosphate homeostasis protein OS=Mus musculus OX=10090 GN=Plpbp PE=1 SV=1 | Plpbp | 0.509 | 0.47 | 0.487 | 1.558 | 1.45 | 1.556 | 3.113 | 4.30E-06 | 0.021659332 | Up |
| Q8C7R4 | Ubiquitin-like modifier-activating enzyme 6 OS=Mus musculus OX=10090 GN=Uba6 PE=1 SV=1 | Uba6 | 0.502 | 0.48 | 0.47 | 1.553 | 1.519 | 1.448 | 3.113 | 2.32E-06 | 0.012117916 | Up |
| Q9CZX0 | Elongator complex protein 3 OS=Mus musculus OX=10090 GN=Elp3 PE=1 SV=1 | Elp3 | 0.479 | 0.498 | 0.478 | 1.478 | 1.482 | 1.568 | 3.112 | 1.09E-06 | 0.005824754 | Up |
| Q6PAR5 | GTPase-activating protein and VPS9 domain-containing protein 1 OS=Mus musculus OX=10090 GN=Gapvd1 PE=1 SV=2 | Gapvd1 | 0.497 | 0.481 | 0.494 | 1.454 | 1.602 | 1.52 | 3.109 | 2.84E-06 | 0.01467087 | Up |
| Q922H9 | Zinc finger protein 330 OS=Mus musculus OX=10090 GN=Znf330 PE=1 SV=1 | Znf330 | 0.495 | 0.453 | 0.495 | 1.536 | 1.427 | 1.523 | 3.109 | 7.19E-06 | 0.034823195 | Up |
| P54775 | 26S proteasome regulatory subunit 6B OS=Mus musculus OX=10090 GN=Psmc4 PE=1 SV=2 | Psmc4 | 0.48 | 0.508 | 0.462 | 1.53 | 1.527 | 1.429 | 3.094 | 5.85E-06 | 0.028811618 | Up |
| Q6NZC7 | SEC23-interacting protein OS=Mus musculus OX=10090 GN=Sec23ip PE=1 SV=2 | Sec23ip | 0.489 | 0.493 | 0.478 | 1.559 | 1.492 | 1.466 | 3.094 | 6.50E-07 | 0.003531889 | Up |
| Q9WU28 | Prefoldin subunit 5 OS=Mus musculus OX=10090 GN=Pfdn5 PE=1 SV=1 | Pfdn5 | 0.492 | 0.485 | 0.5 | 1.507 | 1.456 | 1.601 | 3.09 | 2.65E-06 | 0.013755837 | Up |
| P56376 | Acylphosphatase-1 OS=Mus musculus OX=10090 GN=Acyp1 PE=1 SV=2 | Acyp1 | 0.496 | 0.493 | 0.475 | 1.612 | 1.458 | 1.452 | 3.089 | 6.75E-06 | 0.032885824 | Up |
| Q9R1P0 | Proteasome subunit alpha type-4 OS=Mus musculus OX=10090 GN=Psma4 PE=1 SV=1 | Psma4 | 0.528 | 0.469 | 0.472 | 1.502 | 1.543 | 1.49 | 3.087 | 9.30E-06 | 0.044048842 | Up |
| P54923 | [Protein ADP-ribosylarginine] hydrolase OS=Mus musculus OX=10090 GN=Adprh PE=1 SV=1 | Adprh | 0.461 | 0.49 | 0.495 | 1.49 | 1.519 | 1.45 | 3.084 | 1.70E-06 | 0.008946256 | Up |
| P28474 | Alcohol dehydrogenase class-3 OS=Mus musculus OX=10090 GN=Adh5 PE=1 SV=3 | Adh5 | 0.5 | 0.507 | 0.462 | 1.508 | 1.536 | 1.487 | 3.084 | 3.17E-06 | 0.016275224 | Up |
| P70460 | Vasodilator-stimulated phosphoprotein OS=Mus musculus OX=10090 GN=Vasp PE=1 SV=4 | Vasp | 0.504 | 0.506 | 0.469 | 1.517 | 1.49 | 1.55 | 3.081 | 2.03E-06 | 0.010677173 | Up |
| Q7TQK5 | Coiled-coil domain-containing protein 93 OS=Mus musculus OX=10090 GN=Ccdc93 PE=1 SV=1 | Ccdc93 | 0.47 | 0.512 | 0.485 | 1.558 | 1.475 | 1.485 | 3.08 | 3.17E-06 | 0.016247194 | Up |
| O55029 | Coatomer subunit beta' OS=Mus musculus OX=10090 GN=Copb2 PE=1 SV=2 | Copb2 | 0.495 | 0.493 | 0.487 | 1.501 | 1.511 | 1.51 | 3.066 | 3.08E-09 | 1.73E-05 | Up |
| Q9R0N0 | Galactokinase OS=Mus musculus OX=10090 GN=Galk1 PE=1 SV=2 | Galk1 | 0.509 | 0.483 | 0.496 | 1.537 | 1.501 | 1.516 | 3.06 | 2.92E-07 | 0.001615636 | Up |
| Q9D706 | RNA polymerase II-associated protein 3 OS=Mus musculus OX=10090 GN=Rpap3 PE=1 SV=1 | Rpap3 | 0.472 | 0.507 | 0.473 | 1.502 | 1.518 | 1.422 | 3.059 | 3.53E-06 | 0.018009362 | Up |
| Q3UJB9 | Enhancer of mRNA-decapping protein 4 OS=Mus musculus OX=10090 GN=Edc4 PE=1 SV=2 | Edc4 | 0.512 | 0.482 | 0.466 | 1.502 | 1.481 | 1.477 | 3.055 | 2.36E-06 | 0.012313593 | Up |
| Q64727 | Vinculin OS=Mus musculus OX=10090 GN=Vcl PE=1 SV=4 | Vcl | 0.499 | 0.486 | 0.496 | 1.485 | 1.523 | 1.494 | 3.04 | 5.82E-08 | 0.00032524 | Up |
| Q6PAV2 | Probable E3 ubiquitin-protein ligase HERC4 OS=Mus musculus OX=10090 GN=Herc4 PE=1 SV=2 | Herc4 | 0.525 | 0.488 | 0.485 | 1.485 | 1.574 | 1.489 | 3.036 | 3.98E-06 | 0.020124315 | Up |
| O54774 | AP-3 complex subunit delta-1 OS=Mus musculus OX=10090 GN=Ap3d1 PE=1 SV=1 | Ap3d1 | 0.491 | 0.46 | 0.519 | 1.468 | 1.51 | 1.481 | 3.033 | 6.47E-06 | 0.031613612 | Up |
| B2RQC6 | CAD protein OS=Mus musculus OX=10090 GN=Cad PE=1 SV=1 | Cad | 0.519 | 0.484 | 0.5 | 1.476 | 1.568 | 1.502 | 3.025 | 2.12E-06 | 0.011102936 | Up |
| Q9CVB6 | Actin-related protein 2/3 complex subunit 2 OS=Mus musculus OX=10090 GN=Arpc2 PE=1 SV=3 | Arpc2 | 0.519 | 0.484 | 0.478 | 1.462 | 1.534 | 1.468 | 3.014 | 3.20E-06 | 0.016434701 | Up |
| P49722 | Proteasome subunit alpha type-2 OS=Mus musculus OX=10090 GN=Psma2 PE=1 SV=3 | Psma2 | 0.512 | 0.511 | 0.487 | 1.487 | 1.55 | 1.507 | 3.009 | 7.08E-07 | 0.003842444 | Up |
| P27612 | Phospholipase A-2-activating protein OS=Mus musculus OX=10090 GN=Plaa PE=1 SV=4 | Plaa | 0.511 | 0.498 | 0.481 | 1.492 | 1.507 | 1.485 | 3.009 | 4.32E-07 | 0.002370406 | Up |
| Q8VCE2 | GPN-loop GTPase 1 OS=Mus musculus OX=10090 GN=Gpn1 PE=1 SV=1 | Gpn1 | 0.497 | 0.522 | 0.49 | 1.507 | 1.532 | 1.484 | 2.997 | 8.41E-07 | 0.004540403 | Up |
| Q80UM3 | N-alpha-acetyltransferase 15, NatA auxiliary subunit OS=Mus musculus OX=10090 GN=Naa15 PE=1 SV=1 | Naa15 | 0.512 | 0.498 | 0.482 | 1.499 | 1.444 | 1.524 | 2.994 | 1.29E-06 | 0.006888103 | Up |
| Q5XJY5 | Coatomer subunit delta OS=Mus musculus OX=10090 GN=Arcn1 PE=1 SV=2 | Arcn1 | 0.536 | 0.5 | 0.47 | 1.479 | 1.5 | 1.514 | 2.983 | 9.17E-06 | 0.043506345 | Up |
| O55234 | Proteasome subunit beta type-5 OS=Mus musculus OX=10090 GN=Psmb5 PE=1 SV=3 | Psmb5 | 0.506 | 0.499 | 0.482 | 1.393 | 1.552 | 1.472 | 2.97 | 5.95E-06 | 0.029257622 | Up |
| Q9JJ28 | Protein flightless-1 homolog OS=Mus musculus OX=10090 GN=Flii PE=1 SV=1 | Flii | 0.504 | 0.462 | 0.512 | 1.452 | 1.457 | 1.475 | 2.966 | 4.63E-06 | 0.023217717 | Up |
| Q9JHU4 | Cytoplasmic dynein 1 heavy chain 1 OS=Mus musculus OX=10090 GN=Dync1h1 PE=1 SV=2 | Dync1h1 | 0.522 | 0.498 | 0.499 | 1.48 | 1.506 | 1.503 | 2.955 | 3.08E-07 | 0.001702176 | Up |
| Q9Z1Q5 | Chloride intracellular channel protein 1 OS=Mus musculus OX=10090 GN=Clic1 PE=1 SV=3 | Clic1 | 0.539 | 0.503 | 0.488 | 1.57 | 1.469 | 1.47 | 2.947 | 7.96E-06 | 0.038222143 | Up |
| Q8BK64 | Activator of 90 kDa heat shock protein ATPase homolog 1 OS=Mus musculus OX=10090 GN=Ahsa1 PE=1 SV=2 | Ahsa1 | 0.526 | 0.5 | 0.498 | 1.454 | 1.533 | 1.497 | 2.942 | 1.30E-06 | 0.006950536 | Up |
| Q9DBC7 | cAMP-dependent protein kinase type I-alpha regulatory subunit OS=Mus musculus OX=10090 GN=Prkar1a PE=1 SV=3 | Prkar1a | 0.532 | 0.492 | 0.496 | 1.492 | 1.52 | 1.457 | 2.94 | 2.59E-06 | 0.013423199 | Up |
| O08539 | Myc box-dependent-interacting protein 1 OS=Mus musculus OX=10090 GN=Bin1 PE=1 SV=1 | Bin1 | 0.483 | 0.511 | 0.534 | 1.526 | 1.456 | 1.489 | 2.926 | 4.73E-06 | 0.023663089 | Up |
| Q9ES52 | Phosphatidylinositol 3,4,5-trisphosphate 5-phosphatase 1 OS=Mus musculus OX=10090 GN=Inpp5d PE=1 SV=2 | Inpp5d | 0.514 | 0.502 | 0.51 | 1.511 | 1.464 | 1.474 | 2.915 | 9.03E-08 | 0.000503278 | Up |
| Q6P1F6 | Serine/threonine-protein phosphatase 2A 55 kDa regulatory subunit B alpha isoform OS=Mus musculus OX=10090 GN=Ppp2r2a PE=1 SV=1 | Ppp2r2a | 0.494 | 0.537 | 0.518 | 1.465 | 1.571 | 1.468 | 2.908 | 5.67E-06 | 0.027991387 | Up |
| P61082 | NEDD8-conjugating enzyme Ubc12 OS=Mus musculus OX=10090 GN=Ube2m PE=1 SV=1 | Ube2m | 0.517 | 0.533 | 0.486 | 1.501 | 1.493 | 1.457 | 2.898 | 3.14E-06 | 0.016101563 | Up |
| Q9DCL9 | Multifunctional protein ADE2 OS=Mus musculus OX=10090 GN=Paics PE=1 SV=4 | Paics | 0.505 | 0.518 | 0.516 | 1.396 | 1.554 | 1.496 | 2.889 | 5.18E-06 | 0.025776998 | Up |
| Q8K2T1 | NmrA-like family domain-containing protein 1 OS=Mus musculus OX=10090 GN=Nmral1 PE=1 SV=1 | Nmral1 | 0.511 | 0.526 | 0.503 | 1.462 | 1.552 | 1.432 | 2.887 | 2.69E-06 | 0.01392336 | Up |
| Q8R1F1 | Protein Niban 2 OS=Mus musculus OX=10090 GN=Niban2 PE=1 SV=2 | Niban2 | 0.54 | 0.491 | 0.502 | 1.545 | 1.435 | 1.438 | 2.882 | 9.49E-06 | 0.044812384 | Up |
| P61222 | ATP-binding cassette sub-family E member 1 OS=Mus musculus OX=10090 GN=Abce1 PE=1 SV=1 | Abce1 | 0.532 | 0.53 | 0.49 | 1.507 | 1.501 | 1.452 | 2.874 | 3.53E-06 | 0.017989077 | Up |
| Q6NSR8 | Probable aminopeptidase NPEPL1 OS=Mus musculus OX=10090 GN=Npepl1 PE=1 SV=1 | Npepl1 | 0.504 | 0.518 | 0.538 | 1.537 | 1.476 | 1.466 | 2.871 | 1.60E-06 | 0.008483394 | Up |
| P63073 | Eukaryotic translation initiation factor 4E OS=Mus musculus OX=10090 GN=Eif4e PE=1 SV=1 | Eif4e | 0.542 | 0.522 | 0.496 | 1.414 | 1.545 | 1.518 | 2.87 | 9.36E-06 | 0.044304607 | Up |
| Q8CIE6 | Coatomer subunit alpha OS=Mus musculus OX=10090 GN=Copa PE=1 SV=2 | Copa | 0.507 | 0.526 | 0.53 | 1.522 | 1.5 | 1.462 | 2.869 | 5.16E-07 | 0.002820529 | Up |
| Q99KN9 | Clathrin interactor 1 OS=Mus musculus OX=10090 GN=Clint1 PE=1 SV=2 | Clint1 | 0.529 | 0.544 | 0.509 | 1.528 | 1.525 | 1.461 | 2.853 | 1.70E-06 | 0.00894741 | Up |
| Q3TJD7 | PDZ and LIM domain protein 7 OS=Mus musculus OX=10090 GN=Pdlim7 PE=1 SV=1 | Pdlim7 | 0.526 | 0.499 | 0.532 | 1.527 | 1.43 | 1.483 | 2.852 | 2.78E-06 | 0.014394141 | Up |
| P59016 | Vacuolar protein sorting-associated protein 33B OS=Mus musculus OX=10090 GN=Vps33b PE=1 SV=1 | Vps33b | 0.522 | 0.53 | 0.516 | 1.467 | 1.446 | 1.557 | 2.851 | 1.63E-06 | 0.008614436 | Up |
| Q6P9R2 | Serine/threonine-protein kinase OSR1 OS=Mus musculus OX=10090 GN=Oxsr1 PE=1 SV=1 | Oxsr1 | 0.554 | 0.544 | 0.524 | 1.644 | 1.469 | 1.502 | 2.845 | 1.05E-05 | 0.049270036 | Up |
| Q3UPL0 | Protein transport protein Sec31A OS=Mus musculus OX=10090 GN=Sec31a PE=1 SV=2 | Sec31a | 0.517 | 0.522 | 0.524 | 1.458 | 1.506 | 1.482 | 2.845 | 5.37E-08 | 0.000300336 | Up |
| P58058 | NAD kinase OS=Mus musculus OX=10090 GN=Nadk PE=1 SV=2 | Nadk | 0.53 | 0.528 | 0.498 | 1.48 | 1.506 | 1.44 | 2.844 | 1.66E-06 | 0.008777314 | Up |
| Q91YI0 | Argininosuccinate lyase OS=Mus musculus OX=10090 GN=Asl PE=1 SV=1 | Asl | 0.506 | 0.522 | 0.526 | 1.512 | 1.47 | 1.434 | 2.842 | 7.06E-07 | 0.003834154 | Up |
| P31938 | Dual specificity mitogen-activated protein kinase kinase 1 OS=Mus musculus OX=10090 GN=Map2k1 PE=1 SV=2 | Map2k1 | 0.518 | 0.55 | 0.535 | 1.534 | 1.571 | 1.449 | 2.841 | 3.85E-06 | 0.019522792 | Up |
| Q8R1F5 | Putative hydroxypyruvate isomerase OS=Mus musculus OX=10090 GN=Hyi PE=1 SV=2 | Hyi | 0.517 | 0.546 | 0.527 | 1.546 | 1.482 | 1.479 | 2.835 | 1.09E-06 | 0.005860281 | Up |
| Q8R1X6 | Spartin OS=Mus musculus OX=10090 GN=Spart PE=1 SV=1 | Spart | 0.535 | 0.536 | 0.514 | 1.571 | 1.479 | 1.442 | 2.834 | 3.52E-06 | 0.017964346 | Up |
| Q8BUK6 | Protein Hook homolog 3 OS=Mus musculus OX=10090 GN=Hook3 PE=1 SV=2 | Hook3 | 0.51 | 0.538 | 0.497 | 1.526 | 1.383 | 1.464 | 2.83 | 9.38E-06 | 0.044382808 | Up |
| Q9Z1E4 | Glycogen [starch] synthase, muscle OS=Mus musculus OX=10090 GN=Gys1 PE=1 SV=2 | Gys1 | 0.531 | 0.5 | 0.539 | 1.488 | 1.474 | 1.474 | 2.825 | 1.48E-06 | 0.007849798 | Up |
| Q9D5V5 | Cullin-5 OS=Mus musculus OX=10090 GN=Cul5 PE=1 SV=3 | Cul5 | 0.527 | 0.51 | 0.539 | 1.493 | 1.435 | 1.522 | 2.824 | 1.60E-06 | 0.00848851 | Up |
| P97770 | THUMP domain-containing protein 3 OS=Mus musculus OX=10090 GN=Thumpd3 PE=1 SV=1 | Thumpd3 | 0.523 | 0.484 | 0.543 | 1.448 | 1.49 | 1.433 | 2.82 | 8.45E-06 | 0.04041746 | Up |
| P28063 | Proteasome subunit beta type-8 OS=Mus musculus OX=10090 GN=Psmb8 PE=1 SV=2 | Psmb8 | 0.512 | 0.517 | 0.537 | 1.377 | 1.506 | 1.5 | 2.799 | 6.04E-06 | 0.029650299 | Up |
| Q80UU1 | Ankyrin repeat and zinc finger domain-containing protein 1 OS=Mus musculus OX=10090 GN=Ankzf1 PE=1 SV=2 | Ankzf1 | 0.563 | 0.512 | 0.508 | 1.499 | 1.47 | 1.456 | 2.795 | 7.21E-06 | 0.034905971 | Up |
| Q8VI75 | Importin-4 OS=Mus musculus OX=10090 GN=Ipo4 PE=1 SV=1 | Ipo4 | 0.552 | 0.52 | 0.498 | 1.498 | 1.484 | 1.404 | 2.794 | 9.01E-06 | 0.042803712 | Up |
| Q9WUA3 | ATP-dependent 6-phosphofructokinase, platelet type OS=Mus musculus OX=10090 GN=Pfkp PE=1 SV=1 | Pfkp | 0.532 | 0.516 | 0.541 | 1.484 | 1.435 | 1.518 | 2.792 | 1.13E-06 | 0.0060589 | Up |
| P14685 | 26S proteasome non-ATPase regulatory subunit 3 OS=Mus musculus OX=10090 GN=Psmd3 PE=1 SV=3 | Psmd3 | 0.562 | 0.51 | 0.514 | 1.494 | 1.458 | 1.47 | 2.788 | 5.60E-06 | 0.027707659 | Up |
| Q8R1N4 | NudC domain-containing protein 3 OS=Mus musculus OX=10090 GN=Nudcd3 PE=1 SV=3 | Nudcd3 | 0.526 | 0.506 | 0.529 | 1.535 | 1.439 | 1.37 | 2.783 | 8.91E-06 | 0.042397074 | Up |
| Q9JIF7 | Coatomer subunit beta OS=Mus musculus OX=10090 GN=Copb1 PE=1 SV=1 | Copb1 | 0.533 | 0.538 | 0.512 | 1.455 | 1.464 | 1.464 | 2.769 | 3.08E-07 | 0.001699904 | Up |
| Q5SUR0 | Phosphoribosylformylglycinamidine synthase OS=Mus musculus OX=10090 GN=Pfas PE=1 SV=1 | Pfas | 0.535 | 0.518 | 0.548 | 1.506 | 1.506 | 1.42 | 2.768 | 2.35E-06 | 0.012258688 | Up |
| A2AN08 | E3 ubiquitin-protein ligase UBR4 OS=Mus musculus OX=10090 GN=Ubr4 PE=1 SV=1 | Ubr4 | 0.554 | 0.519 | 0.515 | 1.426 | 1.454 | 1.516 | 2.768 | 4.12E-06 | 0.020801496 | Up |
| P97765 | WW domain-binding protein 2 OS=Mus musculus OX=10090 GN=Wbp2 PE=1 SV=1 | Wbp2 | 0.538 | 0.528 | 0.538 | 1.534 | 1.463 | 1.439 | 2.766 | 9.23E-07 | 0.004974155 | Up |
| Q9WU81 | Glucose-6-phosphate exchanger SLC37A2 OS=Mus musculus OX=10090 GN=Slc37a2 PE=1 SV=1 | Slc37a2 | 0.507 | 0.528 | 0.538 | 1.39 | 1.532 | 1.422 | 2.762 | 7.67E-06 | 0.036949442 | Up |
| Q8BTM8 | Filamin-A OS=Mus musculus OX=10090 GN=Flna PE=1 SV=5 | Flna | 0.523 | 0.563 | 0.512 | 1.495 | 1.434 | 1.474 | 2.755 | 5.38E-06 | 0.026669742 | Up |
| Q9QXB9 | Developmentally-regulated GTP-binding protein 2 OS=Mus musculus OX=10090 GN=Drg2 PE=1 SV=1 | Drg2 | 0.521 | 0.556 | 0.506 | 1.438 | 1.435 | 1.482 | 2.751 | 4.43E-06 | 0.022270086 | Up |
| P26043 | Radixin OS=Mus musculus OX=10090 GN=Rdx PE=1 SV=3 | Rdx | 0.56 | 0.517 | 0.515 | 1.401 | 1.517 | 1.46 | 2.75 | 9.20E-06 | 0.043627632 | Up |
| P97814 | Proline-serine-threonine phosphatase-interacting protein 1 OS=Mus musculus OX=10090 GN=Pstpip1 PE=1 SV=1 | Pstpip1 | 0.535 | 0.531 | 0.548 | 1.453 | 1.504 | 1.478 | 2.748 | 2.06E-07 | 0.001143497 | Up |
| P47713 | Cytosolic phospholipase A2 OS=Mus musculus OX=10090 GN=Pla2g4a PE=1 SV=1 | Pla2g4a | 0.517 | 0.54 | 0.55 | 1.465 | 1.511 | 1.433 | 2.744 | 1.89E-06 | 0.00995164 | Up |
| Q3TXS7 | 26S proteasome non-ATPase regulatory subunit 1 OS=Mus musculus OX=10090 GN=Psmd1 PE=1 SV=1 | Psmd1 | 0.558 | 0.524 | 0.523 | 1.515 | 1.498 | 1.388 | 2.742 | 8.41E-06 | 0.040276382 | Up |
| Q9CY64 | Biliverdin reductase A OS=Mus musculus OX=10090 GN=Blvra PE=1 SV=1 | Blvra | 0.549 | 0.537 | 0.522 | 1.484 | 1.48 | 1.437 | 2.737 | 5.94E-07 | 0.003234624 | Up |
| Q3TRM8 | Hexokinase-3 OS=Mus musculus OX=10090 GN=Hk3 PE=1 SV=2 | Hk3 | 0.552 | 0.558 | 0.505 | 1.49 | 1.485 | 1.442 | 2.735 | 7.11E-06 | 0.034472092 | Up |
| Q9JHK4 | Geranylgeranyl transferase type-2 subunit alpha OS=Mus musculus OX=10090 GN=Rabggta PE=1 SV=1 | Rabggta | 0.55 | 0.543 | 0.529 | 1.467 | 1.481 | 1.476 | 2.727 | 1.14E-07 | 0.000635615 | Up |
| P47941 | Crk-like protein OS=Mus musculus OX=10090 GN=Crkl PE=1 SV=2 | Crkl | 0.554 | 0.534 | 0.534 | 1.44 | 1.452 | 1.53 | 2.726 | 1.54E-06 | 0.008166562 | Up |
| P58252 | Elongation factor 2 OS=Mus musculus OX=10090 GN=Eef2 PE=1 SV=2 | Eef2 | 0.55 | 0.53 | 0.518 | 1.425 | 1.496 | 1.432 | 2.724 | 1.75E-06 | 0.009238903 | Up |
| Q9D8T2 | Gasdermin-D OS=Mus musculus OX=10090 GN=Gsdmdc1 PE=1 SV=1 | Gsdmdc1 | 0.563 | 0.531 | 0.534 | 1.472 | 1.485 | 1.475 | 2.722 | 7.46E-07 | 0.00404511 | Up |
| Q8K2Q9 | Shootin-1 OS=Mus musculus OX=10090 GN=Shtn1 PE=1 SV=1 | Shtn1 | 0.534 | 0.57 | 0.516 | 1.515 | 1.45 | 1.445 | 2.722 | 6.94E-06 | 0.033770203 | Up |
| A2A5R2 | Brefeldin A-inhibited guanine nucleotide-exchange protein 2 OS=Mus musculus OX=10090 GN=Arfgef2 PE=1 SV=1 | Arfgef2 | 0.543 | 0.527 | 0.504 | 1.419 | 1.46 | 1.398 | 2.717 | 2.38E-06 | 0.012394399 | Up |
| Q61792 | LIM and SH3 domain protein 1 OS=Mus musculus OX=10090 GN=Lasp1 PE=1 SV=1 | Lasp1 | 0.551 | 0.535 | 0.524 | 1.524 | 1.426 | 1.423 | 2.716 | 3.10E-06 | 0.015954477 | Up |
| P49138 | MAP kinase-activated protein kinase 2 OS=Mus musculus OX=10090 GN=Mapkapk2 PE=1 SV=2 | Mapkapk2 | 0.557 | 0.511 | 0.545 | 1.495 | 1.445 | 1.435 | 2.712 | 4.14E-06 | 0.020877117 | Up |
| P23780 | Beta-galactosidase OS=Mus musculus OX=10090 GN=Glb1 PE=1 SV=1 | Glb1 | 0.547 | 0.54 | 0.547 | 1.444 | 1.494 | 1.482 | 2.705 | 9.39E-08 | 0.000523442 | Up |
| Q9CQI6 | Coactosin-like protein OS=Mus musculus OX=10090 GN=Cotl1 PE=1 SV=3 | Cotl1 | 0.545 | 0.52 | 0.559 | 1.54 | 1.435 | 1.414 | 2.703 | 7.97E-06 | 0.038243134 | Up |
| E9PYK3 | Protein mono-ADP-ribosyltransferase PARP4 OS=Mus musculus OX=10090 GN=Parp4 PE=1 SV=1 | Parp4 | 0.534 | 0.544 | 0.559 | 1.534 | 1.458 | 1.41 | 2.689 | 3.77E-06 | 0.019150797 | Up |
| O35601 | FYN-binding protein 1 OS=Mus musculus OX=10090 GN=Fyb1 PE=1 SV=2 | Fyb1 | 0.541 | 0.529 | 0.582 | 1.497 | 1.495 | 1.446 | 2.686 | 5.74E-06 | 0.028290922 | Up |
| Q6A065 | Centrosomal protein of 170 kDa OS=Mus musculus OX=10090 GN=Cep170 PE=1 SV=2 | Cep170 | 0.555 | 0.528 | 0.543 | 1.537 | 1.407 | 1.416 | 2.681 | 6.54E-06 | 0.031919592 | Up |
| Q5SWU9 | Acetyl-CoA carboxylase 1 OS=Mus musculus OX=10090 GN=Acaca PE=1 SV=1 | Acaca | 0.551 | 0.555 | 0.528 | 1.483 | 1.452 | 1.428 | 2.67 | 8.41E-07 | 0.004542063 | Up |
| P63242 | Eukaryotic translation initiation factor 5A-1 OS=Mus musculus OX=10090 GN=Eif5a PE=1 SV=2 | Eif5a | 0.58 | 0.528 | 0.551 | 1.494 | 1.472 | 1.462 | 2.669 | 3.88E-06 | 0.019654408 | Up |
| Q922D8 | C-1-tetrahydrofolate synthase, cytoplasmic OS=Mus musculus OX=10090 GN=Mthfd1 PE=1 SV=4 | Mthfd1 | 0.555 | 0.531 | 0.548 | 1.409 | 1.532 | 1.417 | 2.667 | 5.25E-06 | 0.026051256 | Up |
| Q3UPH1 | Protein PRRC1 OS=Mus musculus OX=10090 GN=Prrc1 PE=1 SV=1 | Prrc1 | 0.548 | 0.563 | 0.544 | 1.498 | 1.468 | 1.446 | 2.666 | 2.96E-07 | 0.001637671 | Up |
| P42225 | Signal transducer and activator of transcription 1 OS=Mus musculus OX=10090 GN=Stat1 PE=1 SV=1 | Stat1 | 0.574 | 0.523 | 0.538 | 1.447 | 1.454 | 1.454 | 2.664 | 3.74E-06 | 0.019013592 | Up |
| Q8BWR4 | Ubiquitin carboxyl-terminal hydrolase 40 OS=Mus musculus OX=10090 GN=Usp40 PE=1 SV=2 | Usp40 | 0.544 | 0.565 | 0.54 | 1.509 | 1.424 | 1.457 | 2.662 | 1.50E-06 | 0.007978773 | Up |
| P40142 | Transketolase OS=Mus musculus OX=10090 GN=Tkt PE=1 SV=1 | Tkt | 0.548 | 0.569 | 0.526 | 1.482 | 1.476 | 1.402 | 2.654 | 4.57E-06 | 0.022899986 | Up |
| P17742 | Peptidyl-prolyl cis-trans isomerase A OS=Mus musculus OX=10090 GN=Ppia PE=1 SV=2 | Ppia | 0.56 | 0.554 | 0.54 | 1.507 | 1.432 | 1.443 | 2.649 | 9.05E-07 | 0.004878821 | Up |
| Q3UVL4 | Vacuolar protein sorting-associated protein 51 homolog OS=Mus musculus OX=10090 GN=Vps51 PE=1 SV=2 | Vps51 | 0.553 | 0.584 | 0.525 | 1.469 | 1.493 | 1.438 | 2.647 | 7.47E-06 | 0.036069661 | Up |
| Q61823 | Programmed cell death protein 4 OS=Mus musculus OX=10090 GN=Pdcd4 PE=1 SV=1 | Pdcd4 | 0.556 | 0.58 | 0.52 | 1.436 | 1.478 | 1.459 | 2.641 | 7.78E-06 | 0.037461071 | Up |
| Q80UJ7 | Rab3 GTPase-activating protein catalytic subunit OS=Mus musculus OX=10090 GN=Rab3gap1 PE=1 SV=4 | Rab3gap1 | 0.565 | 0.535 | 0.566 | 1.486 | 1.377 | 1.521 | 2.631 | 1.05E-05 | 0.0492149 | Up |
| P57080 | Ubiquitin carboxyl-terminal hydrolase 25 OS=Mus musculus OX=10090 GN=Usp25 PE=1 SV=2 | Usp25 | 0.564 | 0.525 | 0.572 | 1.487 | 1.403 | 1.465 | 2.622 | 7.01E-06 | 0.034042454 | Up |
| Q9R0P5 | Destrin OS=Mus musculus OX=10090 GN=Dstn PE=1 SV=3 | Dstn | 0.534 | 0.546 | 0.583 | 1.467 | 1.486 | 1.402 | 2.619 | 7.01E-06 | 0.034025471 | Up |
| Q5FWK3 | Rho GTPase-activating protein 1 OS=Mus musculus OX=10090 GN=Arhgap1 PE=1 SV=1 | Arhgap1 | 0.547 | 0.582 | 0.537 | 1.498 | 1.431 | 1.431 | 2.617 | 4.73E-06 | 0.023670765 | Up |
| Q91VU7 | Pseudouridylate synthase 7 homolog OS=Mus musculus OX=10090 GN=Pus7 PE=2 SV=2 | Pus7 | 0.545 | 0.589 | 0.539 | 1.489 | 1.444 | 1.446 | 2.617 | 5.36E-06 | 0.026566496 | Up |
| Q9CR27 | WASH complex subunit 3 OS=Mus musculus OX=10090 GN=Washc3 PE=1 SV=1 | Washc3 | 0.562 | 0.551 | 0.564 | 1.509 | 1.493 | 1.374 | 2.609 | 6.11E-06 | 0.029996903 | Up |
| P47199 | Quinone oxidoreductase OS=Mus musculus OX=10090 GN=Cryz PE=1 SV=1 | Cryz | 0.56 | 0.566 | 0.54 | 1.527 | 1.37 | 1.439 | 2.603 | 1.00E-05 | 0.047146779 | Up |
| Q80UG5 | Septin-9 OS=Mus musculus OX=10090 GN=Septin9 PE=1 SV=1 | Septin9 | 0.55 | 0.55 | 0.57 | 1.422 | 1.522 | 1.39 | 2.595 | 5.65E-06 | 0.027921393 | Up |
| Q9DCR2 | AP-3 complex subunit sigma-1 OS=Mus musculus OX=10090 GN=Ap3s1 PE=1 SV=2 | Ap3s1 | 0.528 | 0.588 | 0.563 | 1.448 | 1.463 | 1.439 | 2.591 | 7.23E-06 | 0.034992551 | Up |
| P26516 | 26S proteasome non-ATPase regulatory subunit 7 OS=Mus musculus OX=10090 GN=Psmd7 PE=1 SV=2 | Psmd7 | 0.56 | 0.555 | 0.554 | 1.355 | 1.505 | 1.457 | 2.587 | 6.96E-06 | 0.033857574 | Up |
| O88522 | NF-kappa-B essential modulator OS=Mus musculus OX=10090 GN=Ikbkg PE=1 SV=2 | Ikbkg | 0.562 | 0.558 | 0.55 | 1.503 | 1.408 | 1.41 | 2.587 | 1.86E-06 | 0.00980387 | Up |
| Q9WTP6 | Adenylate kinase 2, mitochondrial OS=Mus musculus OX=10090 GN=Ak2 PE=1 SV=5 | Ak2 | 0.559 | 0.563 | 0.547 | 1.406 | 1.511 | 1.395 | 2.584 | 3.83E-06 | 0.019446454 | Up |
| Q91WU5 | Arsenite methyltransferase OS=Mus musculus OX=10090 GN=As3mt PE=1 SV=2 | As3mt | 0.568 | 0.558 | 0.564 | 1.472 | 1.407 | 1.47 | 2.573 | 4.57E-07 | 0.002507325 | Up |
| P62192 | 26S proteasome regulatory subunit 4 OS=Mus musculus OX=10090 GN=Psmc1 PE=1 SV=1 | Psmc1 | 0.555 | 0.552 | 0.57 | 1.445 | 1.459 | 1.398 | 2.565 | 5.36E-07 | 0.002931481 | Up |
| Q9D8S4 | Oligoribonuclease, mitochondrial OS=Mus musculus OX=10090 GN=Rexo2 PE=1 SV=2 | Rexo2 | 0.547 | 0.581 | 0.55 | 1.398 | 1.388 | 1.516 | 2.564 | 1.04E-05 | 0.048661899 | Up |
| Q6PGG2 | GEM-interacting protein OS=Mus musculus OX=10090 GN=Gmip PE=1 SV=1 | Gmip | 0.574 | 0.569 | 0.568 | 1.436 | 1.474 | 1.473 | 2.562 | 5.46E-08 | 0.000305203 | Up |
| Q9WTZ1 | RING-box protein 2 OS=Mus musculus OX=10090 GN=Rnf7 PE=3 SV=1 | Rnf7 | 0.542 | 0.565 | 0.589 | 1.466 | 1.39 | 1.485 | 2.56 | 7.41E-06 | 0.035812223 | Up |
| Q99JY9 | Actin-related protein 3 OS=Mus musculus OX=10090 GN=Actr3 PE=1 SV=3 | Actr3 | 0.581 | 0.528 | 0.583 | 1.466 | 1.436 | 1.42 | 2.554 | 9.98E-06 | 0.046920664 | Up |
| Q61210 | Rho guanine nucleotide exchange factor 1 OS=Mus musculus OX=10090 GN=Arhgef1 PE=1 SV=2 | Arhgef1 | 0.581 | 0.561 | 0.529 | 1.395 | 1.41 | 1.456 | 2.55 | 6.46E-06 | 0.031582786 | Up |
| Q80W47 | WD repeat domain phosphoinositide-interacting protein 2 OS=Mus musculus OX=10090 GN=Wipi2 PE=1 SV=1 | Wipi2 | 0.555 | 0.57 | 0.594 | 1.419 | 1.515 | 1.427 | 2.537 | 5.46E-06 | 0.027012606 | Up |
| P70398 | Probable ubiquitin carboxyl-terminal hydrolase FAF-X OS=Mus musculus OX=10090 GN=Usp9x PE=1 SV=2 | Usp9x | 0.579 | 0.555 | 0.537 | 1.372 | 1.432 | 1.422 | 2.529 | 3.41E-06 | 0.017422764 | Up |
| P46471 | 26S proteasome regulatory subunit 7 OS=Mus musculus OX=10090 GN=Psmc2 PE=1 SV=5 | Psmc2 | 0.538 | 0.574 | 0.584 | 1.398 | 1.447 | 1.44 | 2.527 | 4.41E-06 | 0.022175555 | Up |
| Q62245 | Son of sevenless homolog 1 OS=Mus musculus OX=10090 GN=Sos1 PE=1 SV=2 | Sos1 | 0.587 | 0.566 | 0.559 | 1.454 | 1.422 | 1.45 | 2.527 | 5.65E-07 | 0.003083324 | Up |
| P27546 | Microtubule-associated protein 4 OS=Mus musculus OX=10090 GN=Map4 PE=1 SV=3 | Map4 | 0.571 | 0.546 | 0.587 | 1.464 | 1.46 | 1.381 | 2.526 | 5.26E-06 | 0.026076207 | Up |
| Q6NSQ7 | Protein LTV1 homolog OS=Mus musculus OX=10090 GN=Ltv1 PE=1 SV=2 | Ltv1 | 0.571 | 0.588 | 0.545 | 1.47 | 1.418 | 1.406 | 2.52 | 3.72E-06 | 0.018923716 | Up |
| E9QAT4 | Protein transport protein Sec16A OS=Mus musculus OX=10090 GN=Sec16a PE=1 SV=1 | Sec16a | 0.592 | 0.568 | 0.554 | 1.481 | 1.397 | 1.435 | 2.516 | 3.57E-06 | 0.018210199 | Up |
| Q6PNC0 | DmX-like protein 1 OS=Mus musculus OX=10090 GN=Dmxl1 PE=1 SV=1 | Dmxl1 | 0.548 | 0.587 | 0.586 | 1.465 | 1.394 | 1.458 | 2.508 | 4.85E-06 | 0.024204621 | Up |
| P16546 | Spectrin alpha chain, non-erythrocytic 1 OS=Mus musculus OX=10090 GN=Sptan1 PE=1 SV=4 | Sptan1 | 0.602 | 0.544 | 0.578 | 1.439 | 1.4 | 1.476 | 2.503 | 1.01E-05 | 0.047505888 | Up |
| Q80XI3 | Eukaryotic translation initiation factor 4 gamma 3 OS=Mus musculus OX=10090 GN=Eif4g3 PE=1 SV=2 | Eif4g3 | 0.59 | 0.547 | 0.586 | 1.448 | 1.444 | 1.417 | 2.501 | 3.35E-06 | 0.017163111 | Up |
| Q6P2K6 | Serine/threonine-protein phosphatase 4 regulatory subunit 3A OS=Mus musculus OX=10090 GN=Ppp4r3a PE=1 SV=1 | Ppp4r3a | 0.592 | 0.575 | 0.564 | 1.462 | 1.428 | 1.434 | 2.498 | 5.36E-07 | 0.002929727 | Up |
| P61290 | Proteasome activator complex subunit 3 OS=Mus musculus OX=10090 GN=Psme3 PE=1 SV=1 | Psme3 | 0.593 | 0.581 | 0.573 | 1.417 | 1.464 | 1.473 | 2.492 | 5.13E-07 | 0.002805637 | Up |
| Q9D4H8 | Cullin-2 OS=Mus musculus OX=10090 GN=Cul2 PE=1 SV=2 | Cul2 | 0.602 | 0.562 | 0.568 | 1.429 | 1.418 | 1.454 | 2.483 | 2.28E-06 | 0.011904487 | Up |
| P26039 | Talin-1 OS=Mus musculus OX=10090 GN=Tln1 PE=1 SV=2 | Tln1 | 0.584 | 0.578 | 0.568 | 1.426 | 1.47 | 1.4 | 2.483 | 6.29E-07 | 0.003421419 | Up |
| Q8JZQ9 | Eukaryotic translation initiation factor 3 subunit B OS=Mus musculus OX=10090 GN=Eif3b PE=1 SV=1 | Eif3b | 0.577 | 0.602 | 0.552 | 1.473 | 1.432 | 1.39 | 2.481 | 7.17E-06 | 0.034765592 | Up |
| Q69ZR2 | E3 ubiquitin-protein ligase HECTD1 OS=Mus musculus OX=10090 GN=Hectd1 PE=1 SV=2 | Hectd1 | 0.587 | 0.564 | 0.568 | 1.449 | 1.41 | 1.404 | 2.48 | 5.47E-07 | 0.002987069 | Up |
| Q9WU78 | Programmed cell death 6-interacting protein OS=Mus musculus OX=10090 GN=Pdcd6ip PE=1 SV=3 | Pdcd6ip | 0.6 | 0.581 | 0.555 | 1.447 | 1.428 | 1.411 | 2.469 | 2.85E-06 | 0.014705368 | Up |
| Q8CI71 | Syndetin OS=Mus musculus OX=10090 GN=Vps50 PE=1 SV=2 | Vps50 | 0.578 | 0.569 | 0.575 | 1.396 | 1.469 | 1.386 | 2.469 | 1.14E-06 | 0.006104867 | Up |
| Q61183 | Poly(A) polymerase alpha OS=Mus musculus OX=10090 GN=Papola PE=1 SV=4 | Papola | 0.582 | 0.583 | 0.557 | 1.48 | 1.396 | 1.363 | 2.462 | 6.11E-06 | 0.029993154 | Up |
| Q8BFR4 | N-acetylglucosamine-6-sulfatase OS=Mus musculus OX=10090 GN=Gns PE=1 SV=1 | Gns | 0.593 | 0.591 | 0.563 | 1.453 | 1.434 | 1.404 | 2.456 | 1.33E-06 | 0.007090024 | Up |
| P23116 | Eukaryotic translation initiation factor 3 subunit A OS=Mus musculus OX=10090 GN=Eif3a PE=1 SV=5 | Eif3a | 0.586 | 0.595 | 0.564 | 1.401 | 1.433 | 1.44 | 2.449 | 9.80E-07 | 0.005271464 | Up |
| Q9Z1Z0 | General vesicular transport factor p115 OS=Mus musculus OX=10090 GN=Uso1 PE=1 SV=2 | Uso1 | 0.6 | 0.563 | 0.583 | 1.498 | 1.369 | 1.403 | 2.446 | 1.05E-05 | 0.04905725 | Up |
| Q6P8M1 | Putative deoxyribonuclease TATDN1 OS=Mus musculus OX=10090 GN=Tatdn1 PE=1 SV=1 | Tatdn1 | 0.61 | 0.586 | 0.572 | 1.393 | 1.465 | 1.402 | 2.41 | 3.62E-06 | 0.018438584 | Up |
| Q6ZQK5 | Arf-GAP with coiled-coil, ANK repeat and PH domain-containing protein 2 OS=Mus musculus OX=10090 GN=Acap2 PE=1 SV=2 | Acap2 | 0.556 | 0.616 | 0.599 | 1.42 | 1.398 | 1.432 | 2.4 | 9.79E-06 | 0.046113328 | Up |
| B2RX14 | Terminal uridylyltransferase 4 OS=Mus musculus OX=10090 GN=Tut4 PE=1 SV=2 | Tut4 | 0.583 | 0.598 | 0.592 | 1.447 | 1.474 | 1.335 | 2.4 | 9.74E-06 | 0.045898917 | Up |
| Q8N7N5 | DDB1- and CUL4-associated factor 8 OS=Mus musculus OX=10090 GN=Dcaf8 PE=1 SV=1 | Dcaf8 | 0.608 | 0.59 | 0.576 | 1.415 | 1.457 | 1.38 | 2.397 | 2.46E-06 | 0.012815192 | Up |
| Q8R1Q8 | Cytoplasmic dynein 1 light intermediate chain 1 OS=Mus musculus OX=10090 GN=Dync1li1 PE=1 SV=1 | Dync1li1 | 0.602 | 0.562 | 0.566 | 1.368 | 1.41 | 1.368 | 2.397 | 3.41E-06 | 0.017460793 | Up |
| Q6PIP5 | NudC domain-containing protein 1 OS=Mus musculus OX=10090 GN=Nudcd1 PE=1 SV=2 | Nudcd1 | 0.617 | 0.588 | 0.589 | 1.461 | 1.365 | 1.471 | 2.395 | 6.87E-06 | 0.033435219 | Up |
| Q9QZQ1 | Afadin OS=Mus musculus OX=10090 GN=Afdn PE=1 SV=3 | Afdn | 0.597 | 0.565 | 0.594 | 1.347 | 1.433 | 1.423 | 2.394 | 4.93E-06 | 0.024582222 | Up |
| P23475 | X-ray repair cross-complementing protein 6 OS=Mus musculus OX=10090 GN=Xrcc6 PE=1 SV=5 | Xrcc6 | 0.595 | 0.587 | 0.61 | 1.415 | 1.404 | 1.459 | 2.387 | 7.29E-07 | 0.003950939 | Up |
| P50516 | V-type proton ATPase catalytic subunit A OS=Mus musculus OX=10090 GN=Atp6v1a PE=1 SV=2 | Atp6v1a | 0.609 | 0.571 | 0.588 | 1.44 | 1.394 | 1.37 | 2.378 | 3.33E-06 | 0.017029015 | Up |
| Q9JLJ2 | 4-trimethylaminobutyraldehyde dehydrogenase OS=Mus musculus OX=10090 GN=Aldh9a1 PE=1 SV=1 | Aldh9a1 | 0.579 | 0.607 | 0.594 | 1.438 | 1.445 | 1.346 | 2.376 | 5.37E-06 | 0.026625912 | Up |
| Q05769 | Prostaglandin G/H synthase 2 OS=Mus musculus OX=10090 GN=Ptgs2 PE=1 SV=1 | Ptgs2 | 0.586 | 0.616 | 0.58 | 1.404 | 1.44 | 1.368 | 2.364 | 3.48E-06 | 0.017763154 | Up |
| Q80WQ2 | Protein VAC14 homolog OS=Mus musculus OX=10090 GN=Vac14 PE=1 SV=1 | Vac14 | 0.61 | 0.606 | 0.565 | 1.41 | 1.394 | 1.406 | 2.364 | 4.08E-06 | 0.020633774 | Up |
| Q9D662 | Protein transport protein Sec23B OS=Mus musculus OX=10090 GN=Sec23b PE=1 SV=1 | Sec23b | 0.618 | 0.602 | 0.589 | 1.469 | 1.431 | 1.372 | 2.362 | 3.80E-06 | 0.019293057 | Up |
| Q8CIH5 | 1-phosphatidylinositol 4,5-bisphosphate phosphodiesterase gamma-2 OS=Mus musculus OX=10090 GN=Plcg2 PE=1 SV=1 | Plcg2 | 0.592 | 0.614 | 0.597 | 1.398 | 1.464 | 1.393 | 2.36 | 1.57E-06 | 0.008324693 | Up |
| Q8C0D5 | Elongation factor-like GTPase 1 OS=Mus musculus OX=10090 GN=Efl1 PE=1 SV=1 | Efl1 | 0.593 | 0.578 | 0.596 | 1.382 | 1.414 | 1.375 | 2.36 | 2.97E-07 | 0.00164039 | Up |
| P22682 | E3 ubiquitin-protein ligase CBL OS=Mus musculus OX=10090 GN=Cbl PE=1 SV=3 | Cbl | 0.598 | 0.579 | 0.589 | 1.366 | 1.394 | 1.406 | 2.359 | 2.83E-07 | 0.001568623 | Up |
| Q7TMY8 | E3 ubiquitin-protein ligase HUWE1 OS=Mus musculus OX=10090 GN=Huwe1 PE=1 SV=5 | Huwe1 | 0.615 | 0.569 | 0.591 | 1.394 | 1.357 | 1.424 | 2.352 | 5.42E-06 | 0.026862322 | Up |
| P23506 | Protein-L-isoaspartate(D-aspartate) O-methyltransferase OS=Mus musculus OX=10090 GN=Pcmt1 PE=1 SV=3 | Pcmt1 | 0.609 | 0.594 | 0.582 | 1.436 | 1.388 | 1.369 | 2.349 | 1.57E-06 | 0.008308577 | Up |
| P46460 | Vesicle-fusing ATPase OS=Mus musculus OX=10090 GN=Nsf PE=1 SV=2 | Nsf | 0.606 | 0.592 | 0.602 | 1.43 | 1.4 | 1.396 | 2.348 | 1.27E-07 | 0.000705839 | Up |
| B2RY04 | Dedicator of cytokinesis protein 5 OS=Mus musculus OX=10090 GN=Dock5 PE=1 SV=2 | Dock5 | 0.577 | 0.603 | 0.608 | 1.416 | 1.407 | 1.372 | 2.346 | 1.44E-06 | 0.007641694 | Up |
| B1AY13 | Ubiquitin carboxyl-terminal hydrolase 24 OS=Mus musculus OX=10090 GN=Usp24 PE=1 SV=1 | Usp24 | 0.596 | 0.597 | 0.622 | 1.469 | 1.408 | 1.373 | 2.342 | 3.89E-06 | 0.019677829 | Up |
| Q6PB44 | Tyrosine-protein phosphatase non-receptor type 23 OS=Mus musculus OX=10090 GN=Ptpn23 PE=1 SV=2 | Ptpn23 | 0.616 | 0.594 | 0.596 | 1.386 | 1.423 | 1.42 | 2.342 | 4.85E-07 | 0.002658129 | Up |
| A8C756 | Thyroid adenoma-associated protein homolog OS=Mus musculus OX=10090 GN=Thada PE=1 SV=1 | Thada | 0.625 | 0.6 | 0.594 | 1.479 | 1.368 | 1.404 | 2.337 | 6.78E-06 | 0.033027342 | Up |
| Q9QYI3 | DnaJ homolog subfamily C member 7 OS=Mus musculus OX=10090 GN=Dnajc7 PE=1 SV=2 | Dnajc7 | 0.602 | 0.606 | 0.59 | 1.416 | 1.384 | 1.396 | 2.334 | 1.38E-07 | 0.000769632 | Up |
| Q99K70 | Ras-related GTP-binding protein C OS=Mus musculus OX=10090 GN=Rragc PE=1 SV=1 | Rragc | 0.615 | 0.611 | 0.596 | 1.434 | 1.34 | 1.478 | 2.334 | 1.00E-05 | 0.047146779 | Up |
| Q8R0A0 | General transcription factor IIF subunit 2 OS=Mus musculus OX=10090 GN=Gtf2f2 PE=1 SV=1 | Gtf2f2 | 0.611 | 0.614 | 0.587 | 1.408 | 1.464 | 1.353 | 2.332 | 6.04E-06 | 0.02967074 | Up |
| Q8CCP0 | Nuclear export mediator factor Nemf OS=Mus musculus OX=10090 GN=Nemf PE=1 SV=2 | Nemf | 0.575 | 0.613 | 0.619 | 1.376 | 1.425 | 1.404 | 2.327 | 4.76E-06 | 0.02377975 | Up |
| P62814 | V-type proton ATPase subunit B, brain isoform OS=Mus musculus OX=10090 GN=Atp6v1b2 PE=1 SV=1 | Atp6v1b2 | 0.616 | 0.606 | 0.595 | 1.385 | 1.4 | 1.434 | 2.322 | 5.04E-07 | 0.002762506 | Up |
| Q8BIJ7 | RUN and FYVE domain-containing protein 1 OS=Mus musculus OX=10090 GN=Rufy1 PE=1 SV=1 | Rufy1 | 0.553 | 0.594 | 0.599 | 1.343 | 1.347 | 1.362 | 2.321 | 5.17E-06 | 0.025725031 | Up |
| Q9D8U8 | Sorting nexin-5 OS=Mus musculus OX=10090 GN=Snx5 PE=1 SV=1 | Snx5 | 0.568 | 0.604 | 0.626 | 1.404 | 1.374 | 1.386 | 2.316 | 8.52E-06 | 0.040732898 | Up |
| Q812A2 | SLIT-ROBO Rho GTPase-activating protein 3 OS=Mus musculus OX=10090 GN=Srgap3 PE=1 SV=1 | Srgap3 | 0.616 | 0.6 | 0.607 | 1.379 | 1.432 | 1.395 | 2.307 | 4.08E-07 | 0.002244231 | Up |
| P26041 | Moesin OS=Mus musculus OX=10090 GN=Msn PE=1 SV=3 | Msn | 0.623 | 0.597 | 0.59 | 1.362 | 1.41 | 1.397 | 2.303 | 1.78E-06 | 0.009399053 | Up |
| Q05D44 | Eukaryotic translation initiation factor 5B OS=Mus musculus OX=10090 GN=Eif5b PE=1 SV=2 | Eif5b | 0.621 | 0.601 | 0.585 | 1.402 | 1.348 | 1.397 | 2.295 | 2.60E-06 | 0.013487404 | Up |
| Q80TP3 | E3 ubiquitin-protein ligase UBR5 OS=Mus musculus OX=10090 GN=Ubr5 PE=1 SV=2 | Ubr5 | 0.615 | 0.628 | 0.583 | 1.4 | 1.38 | 1.406 | 2.292 | 3.43E-06 | 0.017511769 | Up |
| Q9WVK4 | EH domain-containing protein 1 OS=Mus musculus OX=10090 GN=Ehd1 PE=1 SV=1 | Ehd1 | 0.63 | 0.613 | 0.589 | 1.427 | 1.417 | 1.346 | 2.287 | 6.61E-06 | 0.032256874 | Up |
| Q6NZJ6 | Eukaryotic translation initiation factor 4 gamma 1 OS=Mus musculus OX=10090 GN=Eif4g1 PE=1 SV=1 | Eif4g1 | 0.611 | 0.628 | 0.611 | 1.42 | 1.373 | 1.417 | 2.276 | 5.35E-07 | 0.002926655 | Up |
| Q3THK7 | GMP synthase [glutamine-hydrolyzing] OS=Mus musculus OX=10090 GN=Gmps PE=1 SV=2 | Gmps | 0.611 | 0.612 | 0.608 | 1.359 | 1.416 | 1.384 | 2.271 | 2.79E-07 | 0.001543396 | Up |
| Q8BMG7 | Rab3 GTPase-activating protein non-catalytic subunit OS=Mus musculus OX=10090 GN=Rab3gap2 PE=1 SV=2 | Rab3gap2 | 0.608 | 0.634 | 0.616 | 1.412 | 1.402 | 1.398 | 2.267 | 3.49E-07 | 0.00192339 | Up |
| Q921J2 | GTP-binding protein Rheb OS=Mus musculus OX=10090 GN=Rheb PE=1 SV=1 | Rheb | 0.597 | 0.615 | 0.623 | 1.319 | 1.384 | 1.441 | 2.258 | 8.96E-06 | 0.042585305 | Up |
| P27641 | X-ray repair cross-complementing protein 5 OS=Mus musculus OX=10090 GN=Xrcc5 PE=1 SV=4 | Xrcc5 | 0.587 | 0.622 | 0.627 | 1.358 | 1.411 | 1.374 | 2.257 | 4.25E-06 | 0.021435876 | Up |
| Q9QXK3 | Coatomer subunit gamma-2 OS=Mus musculus OX=10090 GN=Copg2 PE=1 SV=1 | Copg2 | 0.626 | 0.632 | 0.607 | 1.41 | 1.39 | 1.392 | 2.248 | 3.97E-07 | 0.002183697 | Up |
| Q80Y81 | Zinc phosphodiesterase ELAC protein 2 OS=Mus musculus OX=10090 GN=Elac2 PE=1 SV=1 | Elac2 | 0.603 | 0.632 | 0.634 | 1.447 | 1.377 | 1.348 | 2.232 | 7.10E-06 | 0.034418299 | Up |
| Q9CYG7 | Mitochondrial import receptor subunit TOM34 OS=Mus musculus OX=10090 GN=Tomm34 PE=1 SV=1 | Tomm34 | 0.636 | 0.623 | 0.617 | 1.371 | 1.458 | 1.352 | 2.229 | 5.53E-06 | 0.027343242 | Up |
| P16858 | Glyceraldehyde-3-phosphate dehydrogenase OS=Mus musculus OX=10090 GN=Gapdh PE=1 SV=2 | Gapdh | 0.624 | 0.598 | 0.628 | 1.33 | 1.386 | 1.396 | 2.223 | 3.15E-06 | 0.016194484 | Up |
| Q8BWW9 | Serine/threonine-protein kinase N2 OS=Mus musculus OX=10090 GN=Pkn2 PE=1 SV=3 | Pkn2 | 0.632 | 0.599 | 0.642 | 1.356 | 1.37 | 1.434 | 2.221 | 7.95E-06 | 0.038178877 | Up |
| P10711 | Transcription elongation factor A protein 1 OS=Mus musculus OX=10090 GN=Tcea1 PE=1 SV=2 | Tcea1 | 0.595 | 0.616 | 0.64 | 1.393 | 1.36 | 1.351 | 2.217 | 4.16E-06 | 0.020975323 | Up |
| P62196 | 26S proteasome regulatory subunit 8 OS=Mus musculus OX=10090 GN=Psmc5 PE=1 SV=1 | Psmc5 | 0.643 | 0.622 | 0.62 | 1.332 | 1.438 | 1.381 | 2.202 | 6.00E-06 | 0.029491281 | Up |
| O35344 | Importin subunit alpha-4 OS=Mus musculus OX=10090 GN=Kpna3 PE=1 SV=1 | Kpna3 | 0.615 | 0.636 | 0.637 | 1.4 | 1.382 | 1.361 | 2.194 | 6.16E-07 | 0.003350362 | Up |
| P70671 | Interferon regulatory factor 3 OS=Mus musculus OX=10090 GN=Irf3 PE=1 SV=1 | Irf3 | 0.631 | 0.646 | 0.601 | 1.356 | 1.396 | 1.348 | 2.183 | 5.19E-06 | 0.02578234 | Up |
| Q3TIU4 | 2',5'-phosphodiesterase 12 OS=Mus musculus OX=10090 GN=Pde12 PE=1 SV=2 | Pde12 | 0.656 | 0.637 | 0.614 | 1.357 | 1.412 | 1.369 | 2.17 | 4.33E-06 | 0.021786498 | Up |
| Q99KY4 | Cyclin-G-associated kinase OS=Mus musculus OX=10090 GN=Gak PE=1 SV=2 | Gak | 0.617 | 0.637 | 0.637 | 1.396 | 1.35 | 1.344 | 2.163 | 1.11E-06 | 0.005958389 | Up |
| P63017 | Heat shock cognate 71 kDa protein OS=Mus musculus OX=10090 GN=Hspa8 PE=1 SV=1 | Hspa8 | 0.634 | 0.631 | 0.607 | 1.398 | 1.36 | 1.292 | 2.163 | 8.81E-06 | 0.041946217 | Up |
| Q9R0T8 | Inhibitor of nuclear factor kappa-B kinase subunit epsilon OS=Mus musculus OX=10090 GN=Ikbke PE=1 SV=2 | Ikbke | 0.667 | 0.625 | 0.649 | 1.378 | 1.386 | 1.376 | 2.133 | 2.35E-06 | 0.012262019 | Up |
| Q8K2F8 | Protein LSM14 homolog A OS=Mus musculus OX=10090 GN=Lsm14a PE=1 SV=1 | Lsm14a | 0.635 | 0.639 | 0.621 | 1.376 | 1.344 | 1.32 | 2.132 | 8.81E-07 | 0.004755766 | Up |
| Q9R060 | Cytosolic Fe-S cluster assembly factor NUBP1 OS=Mus musculus OX=10090 GN=Nubp1 PE=1 SV=1 | Nubp1 | 0.667 | 0.642 | 0.609 | 1.373 | 1.372 | 1.342 | 2.131 | 1.02E-05 | 0.047979923 | Up |
| Q3TBD2 | Rho GTPase-activating protein 45 OS=Mus musculus OX=10090 GN=Arhgap45 PE=1 SV=2 | Arhgap45 | 0.632 | 0.663 | 0.637 | 1.382 | 1.362 | 1.373 | 2.131 | 1.03E-06 | 0.005530053 | Up |
| Q8VHX6 | Filamin-C OS=Mus musculus OX=10090 GN=Flnc PE=1 SV=3 | Flnc | 0.654 | 0.686 | 0.639 | 1.436 | 1.392 | 1.351 | 2.112 | 1.06E-05 | 0.049563424 | Up |
| Q64378 | Peptidyl-prolyl cis-trans isomerase FKBP5 OS=Mus musculus OX=10090 GN=Fkbp5 PE=1 SV=1 | Fkbp5 | 0.673 | 0.63 | 0.63 | 1.35 | 1.375 | 1.356 | 2.111 | 5.06E-06 | 0.025195841 | Up |
| Q9JI10 | Serine/threonine-protein kinase 3 OS=Mus musculus OX=10090 GN=Stk3 PE=1 SV=1 | Stk3 | 0.632 | 0.666 | 0.615 | 1.332 | 1.36 | 1.346 | 2.111 | 6.47E-06 | 0.031627225 | Up |
| Q64282 | Interferon-induced protein with tetratricopeptide repeats 1 OS=Mus musculus OX=10090 GN=Ifit1 PE=1 SV=2 | Ifit1 | 0.652 | 0.649 | 0.646 | 1.389 | 1.304 | 1.4 | 2.102 | 5.15E-06 | 0.025637696 | Up |
| E9Q2M9 | WD repeat- and FYVE domain-containing protein 4 OS=Mus musculus OX=10090 GN=Wdfy4 PE=1 SV=2 | Wdfy4 | 0.656 | 0.637 | 0.631 | 1.322 | 1.316 | 1.398 | 2.098 | 5.25E-06 | 0.026051256 | Up |
| P46467 | Vacuolar protein sorting-associated protein 4B OS=Mus musculus OX=10090 GN=Vps4b PE=1 SV=2 | Vps4b | 0.646 | 0.647 | 0.628 | 1.359 | 1.367 | 1.296 | 2.094 | 2.88E-06 | 0.01486111 | Up |
| Q924C1 | Exportin-5 OS=Mus musculus OX=10090 GN=Xpo5 PE=1 SV=1 | Xpo5 | 0.655 | 0.679 | 0.652 | 1.328 | 1.402 | 1.406 | 2.083 | 5.35E-06 | 0.026506261 | Up |
| Q8R1B4 | Eukaryotic translation initiation factor 3 subunit C OS=Mus musculus OX=10090 GN=Eif3c PE=1 SV=1 | Eif3c | 0.686 | 0.639 | 0.671 | 1.393 | 1.39 | 1.355 | 2.073 | 5.66E-06 | 0.027970527 | Up |
| Q9R061 | Cytosolic Fe-S cluster assembly factor NUBP2 OS=Mus musculus OX=10090 GN=Nubp2 PE=1 SV=1 | Nubp2 | 0.671 | 0.642 | 0.659 | 1.41 | 1.341 | 1.32 | 2.064 | 6.77E-06 | 0.033015647 | Up |
| Q8BX70 | Vacuolar protein sorting-associated protein 13C OS=Mus musculus OX=10090 GN=Vps13c PE=1 SV=2 | Vps13c | 0.652 | 0.669 | 0.633 | 1.332 | 1.372 | 1.327 | 2.063 | 2.92E-06 | 0.015042843 | Up |
| Q6P8X1 | Sorting nexin-6 OS=Mus musculus OX=10090 GN=Snx6 PE=1 SV=2 | Snx6 | 0.666 | 0.644 | 0.657 | 1.36 | 1.298 | 1.392 | 2.059 | 5.88E-06 | 0.028970551 | Up |
| Q9QZL0 | Receptor-interacting serine/threonine-protein kinase 3 OS=Mus musculus OX=10090 GN=Ripk3 PE=1 SV=2 | Ripk3 | 0.661 | 0.634 | 0.656 | 1.32 | 1.319 | 1.354 | 2.047 | 1.29E-06 | 0.006894063 | Up |
| A3KGF7 | 1-phosphatidylinositol 4,5-bisphosphate phosphodiesterase beta-2 OS=Mus musculus OX=10090 GN=Plcb2 PE=1 SV=1 | Plcb2 | 0.654 | 0.672 | 0.663 | 1.366 | 1.351 | 1.327 | 2.033 | 4.16E-07 | 0.002283993 | Up |
| Q61239 | Protein farnesyltransferase/geranylgeranyltransferase type-1 subunit alpha OS=Mus musculus OX=10090 GN=Fnta PE=1 SV=1 | Fnta | 0.677 | 0.651 | 0.665 | 1.356 | 1.384 | 1.298 | 2.026 | 5.67E-06 | 0.028015401 | Up |
| Q8K0Q5 | Rho GTPase-activating protein 18 OS=Mus musculus OX=10090 GN=Arhgap18 PE=1 SV=1 | Arhgap18 | 0.654 | 0.679 | 0.672 | 1.406 | 1.308 | 1.344 | 2.024 | 7.82E-06 | 0.037601598 | Up |
| Q8BKC8 | Phosphatidylinositol 4-kinase beta OS=Mus musculus OX=10090 GN=Pi4kb PE=1 SV=2 | Pi4kb | 0.658 | 0.66 | 0.681 | 1.355 | 1.352 | 1.326 | 2.018 | 6.94E-07 | 0.003770348 | Up |
| Q9CWQ0 | Diphthine methyl ester synthase OS=Mus musculus OX=10090 GN=Dph5 PE=1 SV=2 | Dph5 | 0.682 | 0.65 | 0.667 | 1.34 | 1.378 | 1.315 | 2.018 | 3.51E-06 | 0.017903301 | Up |
| P15307 | Proto-oncogene c-Rel OS=Mus musculus OX=10090 GN=Rel PE=1 SV=2 | Rel | 0.638 | 0.651 | 0.685 | 1.329 | 1.352 | 1.3 | 2.017 | 8.15E-06 | 0.039031894 | Up |
| P84091 | AP-2 complex subunit mu OS=Mus musculus OX=10090 GN=Ap2m1 PE=1 SV=1 | Ap2m1 | 0.668 | 0.676 | 0.657 | 1.372 | 1.284 | 1.377 | 2.015 | 8.46E-06 | 0.040465041 | Up |
| P49443 | Protein phosphatase 1A OS=Mus musculus OX=10090 GN=Ppm1a PE=1 SV=1 | Ppm1a | 0.689 | 0.674 | 0.64 | 1.34 | 1.372 | 1.316 | 2.011 | 9.70E-06 | 0.045718432 | Up |
| P84078 | ADP-ribosylation factor 1 OS=Mus musculus OX=10090 GN=Arf1 PE=1 SV=2 | Arf1 | 0.693 | 0.656 | 0.677 | 1.364 | 1.339 | 1.369 | 2.01 | 2.26E-06 | 0.011793783 | Up |
| Q9Z0R0 | Serine/threonine-protein kinase haspin OS=Mus musculus OX=10090 GN=Haspin PE=1 SV=3 | Haspin | 1.364 | 1.344 | 1.314 | 0.645 | 0.69 | 0.665 | 0.497 | 6.20E-06 | 0.030380802 | Down |
| Q62376 | U1 small nuclear ribonucleoprotein 70 kDa OS=Mus musculus OX=10090 GN=Snrnp70 PE=1 SV=2 | Snrnp70 | 1.358 | 1.374 | 1.298 | 0.652 | 0.674 | 0.678 | 0.497 | 5.05E-06 | 0.02516988 | Down |
| Q9R0X0 | Mediator of RNA polymerase II transcription subunit 20 OS=Mus musculus OX=10090 GN=Med20 PE=1 SV=1 | Med20 | 1.37 | 1.361 | 1.297 | 0.686 | 0.651 | 0.664 | 0.497 | 7.01E-06 | 0.034044814 | Down |
| Q80WJ7 | Protein LYRIC OS=Mus musculus OX=10090 GN=Mtdh PE=1 SV=1 | Mtdh | 1.341 | 1.33 | 1.336 | 0.669 | 0.66 | 0.658 | 0.496 | 2.48E-08 | 0.000138845 | Down |
| Q61216 | Double-strand break repair protein MRE11 OS=Mus musculus OX=10090 GN=Mre11 PE=1 SV=1 | Mre11 | 1.318 | 1.372 | 1.314 | 0.672 | 0.663 | 0.649 | 0.496 | 2.16E-06 | 0.011318682 | Down |
| Q8CIC2 | Nucleoporin NUP42 OS=Mus musculus OX=10090 GN=Nup42 PE=2 SV=1 | Nup42 | 1.311 | 1.36 | 1.354 | 0.665 | 0.659 | 0.666 | 0.494 | 5.09E-07 | 0.002787366 | Down |
| Q8K327 | Chromosome alignment-maintaining phosphoprotein 1 OS=Mus musculus OX=10090 GN=Champ1 PE=1 SV=1 | Champ1 | 1.308 | 1.363 | 1.339 | 0.654 | 0.661 | 0.662 | 0.493 | 5.90E-07 | 0.003213811 | Down |
| Q9CS00 | Cactin OS=Mus musculus OX=10090 GN=Cactin PE=1 SV=2 | Cactin | 1.289 | 1.368 | 1.296 | 0.666 | 0.647 | 0.63 | 0.492 | 8.94E-06 | 0.042518925 | Down |
| P08775 | DNA-directed RNA polymerase II subunit RPB1 OS=Mus musculus OX=10090 GN=Polr2a PE=1 SV=3 | Polr2a | 1.356 | 1.339 | 1.368 | 0.644 | 0.692 | 0.657 | 0.491 | 5.72E-06 | 0.028214936 | Down |
| Q62311 | Transcription initiation factor TFIID subunit 6 OS=Mus musculus OX=10090 GN=Taf6 PE=1 SV=1 | Taf6 | 1.327 | 1.397 | 1.31 | 0.655 | 0.655 | 0.664 | 0.489 | 3.79E-06 | 0.019227695 | Down |
| Q9BCZ4 | Selenoprotein S OS=Mus musculus OX=10090 GN=Selenos PE=1 SV=3 | Selenos | 1.296 | 1.376 | 1.351 | 0.655 | 0.636 | 0.672 | 0.488 | 7.23E-06 | 0.034971723 | Down |
| Q923D5 | WW domain-binding protein 11 OS=Mus musculus OX=10090 GN=Wbp11 PE=1 SV=2 | Wbp11 | 1.346 | 1.374 | 1.317 | 0.641 | 0.673 | 0.656 | 0.488 | 2.72E-06 | 0.014094486 | Down |
| Q6PDQ2 | Chromodomain-helicase-DNA-binding protein 4 OS=Mus musculus OX=10090 GN=Chd4 PE=1 SV=1 | Chd4 | 1.32 | 1.382 | 1.362 | 0.638 | 0.657 | 0.679 | 0.486 | 5.63E-06 | 0.027827858 | Down |
| Q8K2A7 | Integrator complex subunit 10 OS=Mus musculus OX=10090 GN=Ints10 PE=1 SV=3 | Ints10 | 1.357 | 1.362 | 1.343 | 0.671 | 0.648 | 0.657 | 0.486 | 3.23E-07 | 0.001779602 | Down |
| Q8BN21 | Serine/threonine-protein kinase VRK2 OS=Mus musculus OX=10090 GN=Vrk2 PE=1 SV=2 | Vrk2 | 1.38 | 1.348 | 1.303 | 0.648 | 0.669 | 0.641 | 0.486 | 4.30E-06 | 0.021644694 | Down |
| Q9CZU3 | Exosome RNA helicase MTR4 OS=Mus musculus OX=10090 GN=Mtrex PE=1 SV=1 | Mtrex | 1.33 | 1.357 | 1.396 | 0.641 | 0.656 | 0.676 | 0.483 | 4.02E-06 | 0.020331921 | Down |
| A2A8U2 | Transmembrane protein 201 OS=Mus musculus OX=10090 GN=Tmem201 PE=1 SV=1 | Tmem201 | 1.336 | 1.356 | 1.369 | 0.672 | 0.652 | 0.638 | 0.483 | 1.64E-06 | 0.008649116 | Down |
| Q03347 | Runt-related transcription factor 1 OS=Mus musculus OX=10090 GN=Runx1 PE=1 SV=1 | Runx1 | 1.371 | 1.273 | 1.325 | 0.65 | 0.637 | 0.627 | 0.482 | 6.81E-06 | 0.03315641 | Down |
| Q99PL5 | Ribosome-binding protein 1 OS=Mus musculus OX=10090 GN=Rrbp1 PE=1 SV=2 | Rrbp1 | 1.382 | 1.344 | 1.322 | 0.669 | 0.647 | 0.633 | 0.481 | 3.80E-06 | 0.01929484 | Down |
| E9PYH6 | Histone-lysine N-methyltransferase SETD1A OS=Mus musculus OX=10090 GN=Setd1a PE=1 SV=1 | Setd1a | 1.318 | 1.35 | 1.392 | 0.628 | 0.649 | 0.674 | 0.481 | 9.17E-06 | 0.043511128 | Down |
| Q99LM9 | Transcriptional adapter 1 OS=Mus musculus OX=10090 GN=Tada1 PE=2 SV=1 | Tada1 | 1.343 | 1.338 | 1.387 | 0.641 | 0.656 | 0.657 | 0.48 | 7.79E-07 | 0.004218611 | Down |
| Q07797 | Galectin-3-binding protein OS=Mus musculus OX=10090 GN=Lgals3bp PE=1 SV=1 | Lgals3bp | 1.362 | 1.384 | 1.318 | 0.65 | 0.63 | 0.664 | 0.478 | 3.90E-06 | 0.019748876 | Down |
| Q9QYI4 | DnaJ homolog subfamily B member 12 OS=Mus musculus OX=10090 GN=Dnajb12 PE=1 SV=2 | Dnajb12 | 1.408 | 1.37 | 1.346 | 0.669 | 0.632 | 0.667 | 0.477 | 5.24E-06 | 0.026025167 | Down |
| Q5SFM8 | RNA-binding protein 27 OS=Mus musculus OX=10090 GN=Rbm27 PE=1 SV=3 | Rbm27 | 1.33 | 1.36 | 1.352 | 0.634 | 0.649 | 0.643 | 0.476 | 1.63E-07 | 0.000908064 | Down |
| Q8VBT0 | Thioredoxin-related transmembrane protein 1 OS=Mus musculus OX=10090 GN=Tmx1 PE=1 SV=1 | Tmx1 | 1.308 | 1.343 | 1.353 | 0.64 | 0.646 | 0.612 | 0.474 | 2.83E-06 | 0.01462699 | Down |
| Q8CHY6 | Transcriptional repressor p66 alpha OS=Mus musculus OX=10090 GN=Gatad2a PE=1 SV=2 | Gatad2a | 1.337 | 1.369 | 1.362 | 0.627 | 0.652 | 0.647 | 0.473 | 7.25E-07 | 0.003931241 | Down |
| Q9D787 | RING-type E3 ubiquitin-protein ligase PPIL2 OS=Mus musculus OX=10090 GN=Ppil2 PE=1 SV=2 | Ppil2 | 1.327 | 1.309 | 1.384 | 0.628 | 0.659 | 0.616 | 0.473 | 8.89E-06 | 0.042305833 | Down |
| Q61550 | Double-strand-break repair protein rad21 homolog OS=Mus musculus OX=10090 GN=Rad21 PE=1 SV=3 | Rad21 | 1.346 | 1.358 | 1.295 | 0.612 | 0.644 | 0.635 | 0.473 | 3.73E-06 | 0.018969349 | Down |
| Q8BMQ2 | General transcription factor 3C polypeptide 4 OS=Mus musculus OX=10090 GN=Gtf3c4 PE=1 SV=2 | Gtf3c4 | 1.354 | 1.372 | 1.356 | 0.631 | 0.649 | 0.648 | 0.472 | 1.92E-07 | 0.001067346 | Down |
| Q9QXK2 | E3 ubiquitin-protein ligase RAD18 OS=Mus musculus OX=10090 GN=Rad18 PE=1 SV=2 | Rad18 | 1.338 | 1.349 | 1.367 | 0.611 | 0.669 | 0.635 | 0.472 | 9.97E-06 | 0.04687809 | Down |
| Q3UWW6 | GAS2-like protein 3 OS=Mus musculus OX=10090 GN=Gas2l3 PE=1 SV=1 | Gas2l3 | 1.38 | 1.328 | 1.331 | 0.64 | 0.636 | 0.625 | 0.471 | 7.82E-07 | 0.004230341 | Down |
| Q3UH06 | Ras-responsive element-binding protein 1 OS=Mus musculus OX=10090 GN=Rreb1 PE=1 SV=2 | Rreb1 | 1.322 | 1.413 | 1.373 | 0.649 | 0.625 | 0.658 | 0.47 | 6.81E-06 | 0.03315641 | Down |
| Q921M3 | Splicing factor 3B subunit 3 OS=Mus musculus OX=10090 GN=Sf3b3 PE=1 SV=1 | Sf3b3 | 1.346 | 1.34 | 1.383 | 0.622 | 0.66 | 0.625 | 0.469 | 3.81E-06 | 0.019310426 | Down |
| Q922S8 | Kinesin-like protein KIF2C OS=Mus musculus OX=10090 GN=Kif2c PE=1 SV=1 | Kif2c | 1.386 | 1.322 | 1.337 | 0.646 | 0.605 | 0.645 | 0.469 | 8.09E-06 | 0.038811305 | Down |
| Q9EPU4 | Cleavage and polyadenylation specificity factor subunit 1 OS=Mus musculus OX=10090 GN=Cpsf1 PE=1 SV=1 | Cpsf1 | 1.373 | 1.357 | 1.372 | 0.652 | 0.635 | 0.633 | 0.468 | 1.89E-07 | 0.001047946 | Down |
| Q9R0U0 | Serine/arginine-rich splicing factor 10 OS=Mus musculus OX=10090 GN=Srsf10 PE=1 SV=2 | Srsf10 | 1.387 | 1.379 | 1.397 | 0.662 | 0.634 | 0.652 | 0.468 | 5.46E-07 | 0.00298294 | Down |
| Q9DBA9 | General transcription factor IIH subunit 1 OS=Mus musculus OX=10090 GN=Gtf2h1 PE=1 SV=2 | Gtf2h1 | 1.357 | 1.332 | 1.387 | 0.627 | 0.662 | 0.612 | 0.466 | 8.02E-06 | 0.038480446 | Down |
| Q8BZH4 | Pogo transposable element with ZNF domain OS=Mus musculus OX=10090 GN=Pogz PE=1 SV=2 | Pogz | 1.344 | 1.408 | 1.352 | 0.633 | 0.629 | 0.647 | 0.465 | 1.43E-06 | 0.007622612 | Down |
| Q99KS2 | Neugrin OS=Mus musculus OX=10090 GN=Ngrn PE=2 SV=3 | Ngrn | 1.416 | 1.34 | 1.36 | 0.614 | 0.659 | 0.636 | 0.464 | 8.10E-06 | 0.038812128 | Down |
| E9QAG8 | Zinc finger protein 431 OS=Mus musculus OX=10090 GN=Znf431 PE=1 SV=1 | Znf431 | 1.291 | 1.413 | 1.377 | 0.629 | 0.626 | 0.64 | 0.464 | 1.01E-05 | 0.047505888 | Down |
| Q8CGF7 | Transcription elongation regulator 1 OS=Mus musculus OX=10090 GN=Tcerg1 PE=1 SV=2 | Tcerg1 | 1.341 | 1.409 | 1.338 | 0.623 | 0.64 | 0.633 | 0.464 | 2.05E-06 | 0.010774085 | Down |
| Q91YR7 | Pre-mRNA-processing factor 6 OS=Mus musculus OX=10090 GN=Prpf6 PE=1 SV=1 | Prpf6 | 1.382 | 1.327 | 1.346 | 0.649 | 0.619 | 0.611 | 0.463 | 3.89E-06 | 0.019677829 | Down |
| A2AIV2 | Protein virilizer homolog OS=Mus musculus OX=10090 GN=Virma PE=1 SV=1 | Virma | 1.357 | 1.387 | 1.304 | 0.632 | 0.604 | 0.636 | 0.462 | 5.88E-06 | 0.02895767 | Down |
| P70388 | DNA repair protein RAD50 OS=Mus musculus OX=10090 GN=Rad50 PE=1 SV=1 | Rad50 | 1.335 | 1.385 | 1.352 | 0.606 | 0.637 | 0.633 | 0.461 | 2.17E-06 | 0.01137492 | Down |
| P97360 | Transcription factor ETV6 OS=Mus musculus OX=10090 GN=Etv6 PE=1 SV=1 | Etv6 | 1.42 | 1.314 | 1.37 | 0.632 | 0.627 | 0.632 | 0.461 | 4.31E-06 | 0.02169083 | Down |
| Q9D379 | Epoxide hydrolase 1 OS=Mus musculus OX=10090 GN=Ephx1 PE=1 SV=2 | Ephx1 | 1.366 | 1.334 | 1.402 | 0.607 | 0.628 | 0.649 | 0.459 | 5.45E-06 | 0.026998254 | Down |
| Q8VH51 | RNA-binding protein 39 OS=Mus musculus OX=10090 GN=Rbm39 PE=1 SV=2 | Rbm39 | 1.359 | 1.357 | 1.402 | 0.64 | 0.634 | 0.618 | 0.459 | 8.09E-07 | 0.004374428 | Down |
| P56959 | RNA-binding protein FUS OS=Mus musculus OX=10090 GN=Fus PE=1 SV=1 | Fus | 1.389 | 1.405 | 1.338 | 0.65 | 0.634 | 0.605 | 0.457 | 6.90E-06 | 0.033595545 | Down |
| Q6PFD9 | Nuclear pore complex protein Nup98-Nup96 OS=Mus musculus OX=10090 GN=Nup98 PE=1 SV=2 | Nup98 | 1.32 | 1.397 | 1.398 | 0.639 | 0.609 | 0.632 | 0.457 | 5.21E-06 | 0.025881275 | Down |
| P56960 | Exosome component 10 OS=Mus musculus OX=10090 GN=Exosc10 PE=1 SV=2 | Exosc10 | 1.346 | 1.395 | 1.364 | 0.64 | 0.637 | 0.596 | 0.456 | 6.38E-06 | 0.031215047 | Down |
| P97452 | Ribosome biogenesis protein BOP1 OS=Mus musculus OX=10090 GN=Bop1 PE=1 SV=1 | Bop1 | 1.374 | 1.341 | 1.382 | 0.624 | 0.605 | 0.637 | 0.455 | 1.50E-06 | 0.00793507 | Down |
| Q80UK8 | Integrator complex subunit 2 OS=Mus musculus OX=10090 GN=Ints2 PE=1 SV=2 | Ints2 | 1.398 | 1.343 | 1.32 | 0.594 | 0.622 | 0.629 | 0.454 | 5.47E-06 | 0.027065165 | Down |
| Q9Z2N8 | Actin-like protein 6A OS=Mus musculus OX=10090 GN=Actl6a PE=1 SV=2 | Actl6a | 1.314 | 1.402 | 1.413 | 0.637 | 0.622 | 0.607 | 0.452 | 7.85E-06 | 0.037758504 | Down |
| Q9CZ92 | Centromere protein P OS=Mus musculus OX=10090 GN=Cenpp PE=2 SV=1 | Cenpp | 1.366 | 1.378 | 1.408 | 0.649 | 0.612 | 0.611 | 0.451 | 3.34E-06 | 0.017086566 | Down |
| B9EJ86 | Oxysterol-binding protein-related protein 8 OS=Mus musculus OX=10090 GN=Osbpl8 PE=1 SV=1 | Osbpl8 | 1.343 | 1.379 | 1.384 | 0.607 | 0.633 | 0.61 | 0.451 | 1.04E-06 | 0.005583296 | Down |
| E9Q6J5 | Biorientation of chromosomes in cell division protein 1-like 1 OS=Mus musculus OX=10090 GN=Bod1l PE=1 SV=1 | Bod1l | 1.33 | 1.428 | 1.371 | 0.614 | 0.614 | 0.627 | 0.449 | 3.26E-06 | 0.016734442 | Down |
| Q9CXF7 | Chromodomain-helicase-DNA-binding protein 1-like OS=Mus musculus OX=10090 GN=Chd1l PE=1 SV=1 | Chd1l | 1.356 | 1.405 | 1.364 | 0.608 | 0.634 | 0.607 | 0.448 | 1.51E-06 | 0.007998833 | Down |
| Q7TQC5 | Aprataxin OS=Mus musculus OX=10090 GN=Aptx PE=1 SV=2 | Aptx | 1.406 | 1.324 | 1.411 | 0.622 | 0.627 | 0.608 | 0.448 | 3.78E-06 | 0.019188259 | Down |
| P62317 | Small nuclear ribonucleoprotein Sm D2 OS=Mus musculus OX=10090 GN=Snrpd2 PE=1 SV=1 | Snrpd2 | 1.402 | 1.352 | 1.378 | 0.618 | 0.621 | 0.609 | 0.447 | 2.98E-07 | 0.001648123 | Down |
| Q9D2D7 | Zinc finger protein 687 OS=Mus musculus OX=10090 GN=Znf687 PE=1 SV=1 | Znf687 | 1.387 | 1.392 | 1.373 | 0.611 | 0.618 | 0.618 | 0.445 | 1.37E-08 | 7.67E-05 | Down |
| Q9Z103 | Activity-dependent neuroprotector homeobox protein OS=Mus musculus OX=10090 GN=Adnp PE=1 SV=2 | Adnp | 1.376 | 1.403 | 1.392 | 0.619 | 0.643 | 0.587 | 0.443 | 7.22E-06 | 0.034971723 | Down |
| Q69ZR9 | Protein TASOR OS=Mus musculus OX=10090 GN=Tasor PE=1 SV=2 | Tasor | 1.401 | 1.398 | 1.386 | 0.587 | 0.643 | 0.622 | 0.443 | 6.96E-06 | 0.033857574 | Down |
| Q9ESX4 | Nucleolar protein of 40 kDa OS=Mus musculus OX=10090 GN=Zcchc17 PE=1 SV=1 | Zcchc17 | 1.378 | 1.432 | 1.364 | 0.624 | 0.631 | 0.591 | 0.442 | 5.31E-06 | 0.026349256 | Down |
| Q80UV9 | Transcription initiation factor TFIID subunit 1 OS=Mus musculus OX=10090 GN=Taf1 PE=1 SV=2 | Taf1 | 1.35 | 1.384 | 1.374 | 0.582 | 0.639 | 0.596 | 0.442 | 9.46E-06 | 0.044698985 | Down |
| Q9EQ06 | Estradiol 17-beta-dehydrogenase 11 OS=Mus musculus OX=10090 GN=Hsd17b11 PE=1 SV=1 | Hsd17b11 | 1.387 | 1.408 | 1.405 | 0.618 | 0.59 | 0.631 | 0.438 | 2.22E-06 | 0.011611152 | Down |
| Q8BL74 | General transcription factor 3C polypeptide 2 OS=Mus musculus OX=10090 GN=Gtf3c2 PE=2 SV=2 | Gtf3c2 | 1.404 | 1.4 | 1.352 | 0.594 | 0.606 | 0.622 | 0.438 | 1.37E-06 | 0.007274909 | Down |
| Q9DCE5 | p21-activated protein kinase-interacting protein 1 OS=Mus musculus OX=10090 GN=Pak1ip1 PE=1 SV=2 | Pak1ip1 | 1.33 | 1.434 | 1.396 | 0.591 | 0.609 | 0.619 | 0.437 | 5.71E-06 | 0.02815273 | Down |
| Q149L6 | DnaJ homolog subfamily B member 14 OS=Mus musculus OX=10090 GN=Dnajb14 PE=2 SV=1 | Dnajb14 | 1.346 | 1.439 | 1.409 | 0.609 | 0.6 | 0.614 | 0.435 | 2.34E-06 | 0.012226463 | Down |
| Q6P4S8 | Integrator complex subunit 1 OS=Mus musculus OX=10090 GN=Ints1 PE=1 SV=2 | Ints1 | 1.408 | 1.39 | 1.364 | 0.605 | 0.63 | 0.573 | 0.434 | 8.66E-06 | 0.041316692 | Down |
| Q9WVG9 | Male-specific lethal 3 homolog OS=Mus musculus OX=10090 GN=Msl3 PE=1 SV=3 | Msl3 | 1.421 | 1.406 | 1.373 | 0.596 | 0.604 | 0.624 | 0.434 | 1.03E-06 | 0.005550082 | Down |
| Q8BSQ9 | Protein polybromo-1 OS=Mus musculus OX=10090 GN=Pbrm1 PE=1 SV=4 | Pbrm1 | 1.356 | 1.406 | 1.405 | 0.569 | 0.622 | 0.615 | 0.433 | 1.04E-05 | 0.048747934 | Down |
| Q91WM1 | Spermatid perinuclear RNA-binding protein OS=Mus musculus OX=10090 GN=Strbp PE=1 SV=1 | Strbp | 1.408 | 1.403 | 1.506 | 0.629 | 0.625 | 0.609 | 0.432 | 4.74E-06 | 0.023687887 | Down |
| Q8VEK3 | Heterogeneous nuclear ribonucleoprotein U OS=Mus musculus OX=10090 GN=Hnrnpu PE=1 SV=1 | Hnrnpu | 1.357 | 1.431 | 1.399 | 0.597 | 0.609 | 0.602 | 0.432 | 8.78E-07 | 0.004738553 | Down |
| Q8CH02 | SURP and G-patch domain-containing protein 1 OS=Mus musculus OX=10090 GN=Sugp1 PE=1 SV=1 | Sugp1 | 1.389 | 1.432 | 1.387 | 0.585 | 0.641 | 0.592 | 0.432 | 1.04E-05 | 0.048585657 | Down |
| P11680 | Properdin OS=Mus musculus OX=10090 GN=Cfp PE=1 SV=2 | Cfp | 1.455 | 1.378 | 1.372 | 0.621 | 0.586 | 0.6 | 0.43 | 4.81E-06 | 0.024045564 | Down |
| P53564 | Homeobox protein cut-like 1 OS=Mus musculus OX=10090 GN=Cux1 PE=1 SV=3 | Cux1 | 1.392 | 1.405 | 1.357 | 0.575 | 0.627 | 0.586 | 0.43 | 7.50E-06 | 0.036172543 | Down |
| Q8BUH8 | Sentrin-specific protease 7 OS=Mus musculus OX=10090 GN=Senp7 PE=1 SV=1 | Senp7 | 1.378 | 1.41 | 1.419 | 0.607 | 0.609 | 0.592 | 0.43 | 2.98E-07 | 0.001650042 | Down |
| Q922U1 | U4/U6 small nuclear ribonucleoprotein Prp3 OS=Mus musculus OX=10090 GN=Prpf3 PE=1 SV=1 | Prpf3 | 1.358 | 1.423 | 1.402 | 0.597 | 0.595 | 0.604 | 0.429 | 5.22E-07 | 0.002853276 | Down |
| Q9ER69 | Pre-mRNA-splicing regulator WTAP OS=Mus musculus OX=10090 GN=Wtap PE=1 SV=3 | Wtap | 1.405 | 1.414 | 1.392 | 0.617 | 0.57 | 0.609 | 0.427 | 4.37E-06 | 0.021979591 | Down |
| Q8BVE8 | Histone-lysine N-methyltransferase NSD2 OS=Mus musculus OX=10090 GN=Nsd2 PE=1 SV=2 | Nsd2 | 1.389 | 1.461 | 1.39 | 0.611 | 0.598 | 0.598 | 0.426 | 1.24E-06 | 0.006628275 | Down |
| Q8K284 | General transcription factor 3C polypeptide 1 OS=Mus musculus OX=10090 GN=Gtf3c1 PE=1 SV=2 | Gtf3c1 | 1.35 | 1.442 | 1.418 | 0.593 | 0.603 | 0.599 | 0.426 | 1.95E-06 | 0.010268788 | Down |
| Q9D710 | Thioredoxin-related transmembrane protein 2 OS=Mus musculus OX=10090 GN=Tmx2 PE=1 SV=1 | Tmx2 | 1.405 | 1.431 | 1.403 | 0.606 | 0.626 | 0.572 | 0.426 | 6.05E-06 | 0.029704724 | Down |
| Q9ERU9 | E3 SUMO-protein ligase RanBP2 OS=Mus musculus OX=10090 GN=Ranbp2 PE=1 SV=2 | Ranbp2 | 1.366 | 1.427 | 1.401 | 0.568 | 0.605 | 0.61 | 0.425 | 4.97E-06 | 0.024760954 | Down |
| Q9D554 | Splicing factor 3A subunit 3 OS=Mus musculus OX=10090 GN=Sf3a3 PE=1 SV=2 | Sf3a3 | 1.406 | 1.458 | 1.388 | 0.616 | 0.602 | 0.589 | 0.425 | 1.65E-06 | 0.008706479 | Down |
| P70399 | TP53-binding protein 1 OS=Mus musculus OX=10090 GN=Tp53bp1 PE=1 SV=3 | Tp53bp1 | 1.392 | 1.425 | 1.388 | 0.582 | 0.599 | 0.602 | 0.424 | 3.59E-07 | 0.001977198 | Down |
| P62320 | Small nuclear ribonucleoprotein Sm D3 OS=Mus musculus OX=10090 GN=Snrpd3 PE=1 SV=1 | Snrpd3 | 1.383 | 1.353 | 1.394 | 0.603 | 0.589 | 0.558 | 0.424 | 4.04E-06 | 0.020403892 | Down |
| Q99J62 | Replication factor C subunit 4 OS=Mus musculus OX=10090 GN=Rfc4 PE=1 SV=1 | Rfc4 | 1.447 | 1.401 | 1.42 | 0.618 | 0.612 | 0.566 | 0.421 | 7.85E-06 | 0.037727895 | Down |
| Q99K74 | Mediator of RNA polymerase II transcription subunit 24 OS=Mus musculus OX=10090 GN=Med24 PE=1 SV=1 | Med24 | 1.449 | 1.436 | 1.386 | 0.601 | 0.588 | 0.608 | 0.421 | 8.37E-07 | 0.004522641 | Down |
| Q8K4X7 | 1-acyl-sn-glycerol-3-phosphate acyltransferase delta OS=Mus musculus OX=10090 GN=Agpat4 PE=1 SV=1 | Agpat4 | 1.458 | 1.438 | 1.432 | 0.606 | 0.583 | 0.63 | 0.42 | 2.97E-06 | 0.015315795 | Down |
| E9Q5F9 | Histone-lysine N-methyltransferase SETD2 OS=Mus musculus OX=10090 GN=Setd2 PE=1 SV=1 | Setd2 | 1.394 | 1.384 | 1.412 | 0.607 | 0.56 | 0.588 | 0.419 | 3.54E-06 | 0.018028873 | Down |
| Q810V0 | U3 small nucleolar ribonucleoprotein protein MPP10 OS=Mus musculus OX=10090 GN=Mphosph10 PE=1 SV=2 | Mphosph10 | 1.385 | 1.424 | 1.386 | 0.573 | 0.595 | 0.581 | 0.417 | 4.28E-07 | 0.002348676 | Down |
| Q80UZ2 | Protein SDA1 homolog OS=Mus musculus OX=10090 GN=Sdad1 PE=1 SV=1 | Sdad1 | 1.425 | 1.475 | 1.407 | 0.581 | 0.615 | 0.587 | 0.414 | 2.52E-06 | 0.01307514 | Down |
| Q9CZX5 | PIN2/TERF1-interacting telomerase inhibitor 1 OS=Mus musculus OX=10090 GN=Pinx1 PE=1 SV=2 | Pinx1 | 1.421 | 1.418 | 1.434 | 0.582 | 0.619 | 0.566 | 0.414 | 4.95E-06 | 0.024679093 | Down |
| Q69ZK6 | Probable JmjC domain-containing histone demethylation protein 2C OS=Mus musculus OX=10090 GN=Jmjd1c PE=1 SV=3 | Jmjd1c | 1.404 | 1.459 | 1.366 | 0.56 | 0.586 | 0.599 | 0.413 | 5.58E-06 | 0.02758185 | Down |
| Q9D968 | Host cell factor 2 OS=Mus musculus OX=10090 GN=Hcfc2 PE=1 SV=2 | Hcfc2 | 1.36 | 1.378 | 1.439 | 0.575 | 0.551 | 0.597 | 0.412 | 6.63E-06 | 0.032318416 | Down |
| Q8BJ71 | Nuclear pore complex protein Nup93 OS=Mus musculus OX=10090 GN=Nup93 PE=1 SV=1 | Nup93 | 1.428 | 1.43 | 1.385 | 0.571 | 0.58 | 0.597 | 0.412 | 7.53E-07 | 0.004078503 | Down |
| Q6ZPR5 | Sphingomyelin phosphodiesterase 4 OS=Mus musculus OX=10090 GN=Smpd4 PE=1 SV=2 | Smpd4 | 1.374 | 1.443 | 1.404 | 0.56 | 0.598 | 0.575 | 0.411 | 3.03E-06 | 0.015600396 | Down |
| Q61033 | Lamina-associated polypeptide 2, isoforms alpha/zeta OS=Mus musculus OX=10090 GN=Tmpo PE=1 SV=4 | Tmpo | 1.409 | 1.42 | 1.393 | 0.604 | 0.543 | 0.585 | 0.41 | 9.89E-06 | 0.046551524 | Down |
| O88291 | DBIRD complex subunit ZNF326 OS=Mus musculus OX=10090 GN=Znf326 PE=1 SV=1 | Znf326 | 1.386 | 1.412 | 1.448 | 0.566 | 0.6 | 0.573 | 0.41 | 2.13E-06 | 0.011159016 | Down |
| P97363 | Serine palmitoyltransferase 2 OS=Mus musculus OX=10090 GN=Sptlc2 PE=1 SV=2 | Sptlc2 | 1.438 | 1.353 | 1.438 | 0.577 | 0.576 | 0.581 | 0.41 | 1.67E-06 | 0.008804126 | Down |
| Q8R326 | Paraspeckle component 1 OS=Mus musculus OX=10090 GN=Pspc1 PE=1 SV=1 | Pspc1 | 1.41 | 1.439 | 1.442 | 0.58 | 0.573 | 0.6 | 0.409 | 5.42E-07 | 0.002958411 | Down |
| Q99JX7 | Nuclear RNA export factor 1 OS=Mus musculus OX=10090 GN=Nxf1 PE=1 SV=3 | Nxf1 | 1.428 | 1.422 | 1.442 | 0.567 | 0.594 | 0.593 | 0.409 | 5.79E-07 | 0.003158389 | Down |
| Q8BH74 | Nuclear pore complex protein Nup107 OS=Mus musculus OX=10090 GN=Nup107 PE=1 SV=1 | Nup107 | 1.411 | 1.466 | 1.424 | 0.554 | 0.591 | 0.613 | 0.409 | 9.41E-06 | 0.044506651 | Down |
| Q78XF5 | Oligosaccharyltransferase complex subunit OSTC OS=Mus musculus OX=10090 GN=Ostc PE=1 SV=1 | Ostc | 1.418 | 1.433 | 1.417 | 0.557 | 0.57 | 0.617 | 0.409 | 8.75E-06 | 0.041688845 | Down |
| P84104 | Serine/arginine-rich splicing factor 3 OS=Mus musculus OX=10090 GN=Srsf3 PE=1 SV=1 | Srsf3 | 1.4 | 1.449 | 1.39 | 0.588 | 0.573 | 0.568 | 0.408 | 6.87E-07 | 0.003733082 | Down |
| P61965 | WD repeat-containing protein 5 OS=Mus musculus OX=10090 GN=Wdr5 PE=1 SV=1 | Wdr5 | 1.385 | 1.412 | 1.403 | 0.556 | 0.596 | 0.56 | 0.408 | 2.47E-06 | 0.012867109 | Down |
| Q99P88 | Nuclear pore complex protein Nup155 OS=Mus musculus OX=10090 GN=Nup155 PE=1 SV=1 | Nup155 | 1.386 | 1.44 | 1.453 | 0.556 | 0.592 | 0.592 | 0.407 | 3.80E-06 | 0.019308091 | Down |
| Q8BGS0 | Protein MAK16 homolog OS=Mus musculus OX=10090 GN=Mak16 PE=1 SV=1 | Mak16 | 1.409 | 1.427 | 1.455 | 0.546 | 0.597 | 0.594 | 0.405 | 7.61E-06 | 0.036683574 | Down |
| Q3UHX0 | Nucleolar protein 8 OS=Mus musculus OX=10090 GN=Nol8 PE=1 SV=2 | Nol8 | 1.41 | 1.429 | 1.435 | 0.555 | 0.599 | 0.571 | 0.404 | 2.41E-06 | 0.012556906 | Down |
| Q9CZX9 | ER membrane protein complex subunit 4 OS=Mus musculus OX=10090 GN=Emc4 PE=1 SV=1 | Emc4 | 1.376 | 1.432 | 1.479 | 0.575 | 0.579 | 0.576 | 0.404 | 1.71E-06 | 0.00902753 | Down |
| Q8CB77 | Elongin-A OS=Mus musculus OX=10090 GN=Eloa PE=1 SV=3 | Eloa | 1.386 | 1.474 | 1.401 | 0.564 | 0.569 | 0.586 | 0.403 | 2.15E-06 | 0.011259962 | Down |
| Q99LI7 | Cleavage stimulation factor subunit 3 OS=Mus musculus OX=10090 GN=Cstf3 PE=1 SV=1 | Cstf3 | 1.411 | 1.451 | 1.37 | 0.548 | 0.564 | 0.59 | 0.402 | 4.70E-06 | 0.023531754 | Down |
| Q8VE80 | THO complex subunit 3 OS=Mus musculus OX=10090 GN=Thoc3 PE=2 SV=1 | Thoc3 | 1.405 | 1.438 | 1.412 | 0.554 | 0.577 | 0.58 | 0.402 | 5.88E-07 | 0.003203002 | Down |
| P17012 | Zinc finger X-chromosomal protein OS=Mus musculus OX=10090 GN=Zfx PE=1 SV=2 | Zfx | 1.445 | 1.52 | 1.387 | 0.588 | 0.577 | 0.578 | 0.401 | 4.64E-06 | 0.023256336 | Down |
| Q8BK35 | Ribosome biogenesis protein NOP53 OS=Mus musculus OX=10090 GN=Nop53 PE=1 SV=1 | Nop53 | 1.431 | 1.421 | 1.452 | 0.572 | 0.582 | 0.566 | 0.4 | 9.59E-08 | 0.00053478 | Down |
| Q9CPQ5 | Centromere protein Q OS=Mus musculus OX=10090 GN=Cenpq PE=2 SV=2 | Cenpq | 1.471 | 1.387 | 1.45 | 0.561 | 0.577 | 0.58 | 0.399 | 1.48E-06 | 0.007858527 | Down |
| Q6P5D8 | Structural maintenance of chromosomes flexible hinge domain-containing protein 1 OS=Mus musculus OX=10090 GN=Smchd1 PE=1 SV=2 | Smchd1 | 1.43 | 1.466 | 1.396 | 0.546 | 0.6 | 0.567 | 0.399 | 7.54E-06 | 0.036367235 | Down |
| Q9QY81 | Nuclear pore membrane glycoprotein 210 OS=Mus musculus OX=10090 GN=Nup210 PE=1 SV=2 | Nup210 | 1.376 | 1.414 | 1.433 | 0.527 | 0.582 | 0.573 | 0.398 | 9.86E-06 | 0.046412563 | Down |
| Q9CZT6 | Protein CMSS1 OS=Mus musculus OX=10090 GN=Cmss1 PE=2 SV=1 | Cmss1 | 1.419 | 1.423 | 1.422 | 0.57 | 0.577 | 0.54 | 0.396 | 1.39E-06 | 0.00741792 | Down |
| P70255 | Nuclear factor 1 C-type OS=Mus musculus OX=10090 GN=Nfic PE=1 SV=1 | Nfic | 1.469 | 1.387 | 1.428 | 0.585 | 0.555 | 0.554 | 0.395 | 2.84E-06 | 0.014678724 | Down |
| P29037 | TATA-box-binding protein OS=Mus musculus OX=10090 GN=Tbp PE=1 SV=1 | Tbp | 1.465 | 1.45 | 1.403 | 0.537 | 0.57 | 0.598 | 0.395 | 1.03E-05 | 0.048314059 | Down |
| Q6PE01 | U5 small nuclear ribonucleoprotein 40 kDa protein OS=Mus musculus OX=10090 GN=Snrnp40 PE=1 SV=1 | Snrnp40 | 1.355 | 1.481 | 1.4 | 0.577 | 0.556 | 0.539 | 0.395 | 9.00E-06 | 0.042786713 | Down |
| Q8BFQ4 | WD repeat-containing protein 82 OS=Mus musculus OX=10090 GN=Wdr82 PE=1 SV=1 | Wdr82 | 1.416 | 1.444 | 1.428 | 0.542 | 0.587 | 0.566 | 0.395 | 2.55E-06 | 0.013250615 | Down |
| Q6DFW4 | Nucleolar protein 58 OS=Mus musculus OX=10090 GN=Nop58 PE=1 SV=1 | Nop58 | 1.41 | 1.454 | 1.44 | 0.568 | 0.574 | 0.552 | 0.394 | 3.79E-07 | 0.002083678 | Down |
| Q8BU11 | TOX high mobility group box family member 4 OS=Mus musculus OX=10090 GN=Tox4 PE=1 SV=3 | Tox4 | 1.443 | 1.512 | 1.381 | 0.578 | 0.558 | 0.573 | 0.394 | 5.05E-06 | 0.02516988 | Down |
| Q8BY02 | NF-kappa-B-repressing factor OS=Mus musculus OX=10090 GN=Nkrf PE=2 SV=3 | Nkrf | 1.414 | 1.467 | 1.446 | 0.593 | 0.548 | 0.561 | 0.393 | 3.45E-06 | 0.017610184 | Down |
| O88532 | Zinc finger RNA-binding protein OS=Mus musculus OX=10090 GN=Zfr PE=1 SV=2 | Zfr | 1.428 | 1.452 | 1.429 | 0.566 | 0.565 | 0.562 | 0.393 | 9.19E-09 | 5.15E-05 | Down |
| Q9JLV6 | Bifunctional polynucleotide phosphatase/kinase OS=Mus musculus OX=10090 GN=Pnkp PE=1 SV=2 | Pnkp | 1.432 | 1.449 | 1.384 | 0.565 | 0.562 | 0.544 | 0.392 | 8.43E-07 | 0.004552297 | Down |
| Q7TPD0 | Integrator complex subunit 3 OS=Mus musculus OX=10090 GN=Ints3 PE=1 SV=2 | Ints3 | 1.434 | 1.441 | 1.416 | 0.567 | 0.53 | 0.586 | 0.392 | 6.28E-06 | 0.030776204 | Down |
| Q6ZQI3 | Malectin OS=Mus musculus OX=10090 GN=Mlec PE=1 SV=2 | Mlec | 1.432 | 1.464 | 1.43 | 0.555 | 0.58 | 0.559 | 0.392 | 4.62E-07 | 0.002534665 | Down |
| Q7JJ13 | Bromodomain-containing protein 2 OS=Mus musculus OX=10090 GN=Brd2 PE=1 SV=1 | Brd2 | 1.391 | 1.428 | 1.479 | 0.584 | 0.554 | 0.541 | 0.391 | 5.21E-06 | 0.025905534 | Down |
| Q9D0N7 | Chromatin assembly factor 1 subunit B OS=Mus musculus OX=10090 GN=Chaf1b PE=1 SV=1 | Chaf1b | 1.44 | 1.482 | 1.442 | 0.541 | 0.587 | 0.58 | 0.391 | 4.15E-06 | 0.020938516 | Down |
| P11370 | Retrovirus-related Env polyprotein from Fv-4 locus OS=Mus musculus OX=10090 GN=Fv4 PE=1 SV=2 | Fv4 | 1.476 | 1.414 | 1.423 | 0.556 | 0.594 | 0.535 | 0.391 | 9.40E-06 | 0.044464209 | Down |
| Q8C854 | Myelin expression factor 2 OS=Mus musculus OX=10090 GN=Myef2 PE=1 SV=1 | Myef2 | 1.438 | 1.47 | 1.454 | 0.552 | 0.577 | 0.568 | 0.389 | 3.28E-07 | 0.001805651 | Down |
| Q9CYX7 | RRP15-like protein OS=Mus musculus OX=10090 GN=Rrp15 PE=1 SV=2 | Rrp15 | 1.435 | 1.478 | 1.424 | 0.585 | 0.547 | 0.553 | 0.389 | 2.36E-06 | 0.012282756 | Down |
| Q6P4T2 | U5 small nuclear ribonucleoprotein 200 kDa helicase OS=Mus musculus OX=10090 GN=Snrnp200 PE=1 SV=1 | Snrnp200 | 1.411 | 1.464 | 1.426 | 0.55 | 0.562 | 0.562 | 0.389 | 2.23E-07 | 0.001238979 | Down |
| Q60668 | Heterogeneous nuclear ribonucleoprotein D0 OS=Mus musculus OX=10090 GN=Hnrnpd PE=1 SV=2 | Hnrnpd | 1.402 | 1.493 | 1.48 | 0.556 | 0.579 | 0.556 | 0.387 | 2.37E-06 | 0.012369895 | Down |
| Q6NZQ4 | PAX-interacting protein 1 OS=Mus musculus OX=10090 GN=Paxip1 PE=1 SV=1 | Paxip1 | 1.414 | 1.474 | 1.452 | 0.53 | 0.583 | 0.563 | 0.386 | 6.17E-06 | 0.030276953 | Down |
| P20152 | Vimentin OS=Mus musculus OX=10090 GN=Vim PE=1 SV=3 | Vim | 1.443 | 1.485 | 1.431 | 0.574 | 0.547 | 0.562 | 0.386 | 7.48E-07 | 0.004054078 | Down |
| O35691 | Pinin OS=Mus musculus OX=10090 GN=Pnn PE=1 SV=4 | Pnn | 1.46 | 1.437 | 1.406 | 0.541 | 0.578 | 0.536 | 0.385 | 3.34E-06 | 0.017092853 | Down |
| Q8C9B9 | Death-inducer obliterator 1 OS=Mus musculus OX=10090 GN=Dido1 PE=1 SV=4 | Dido1 | 1.448 | 1.41 | 1.491 | 0.557 | 0.553 | 0.566 | 0.385 | 6.85E-07 | 0.003718573 | Down |
| Q8BIH0 | Histone deacetylase complex subunit SAP130 OS=Mus musculus OX=10090 GN=Sap130 PE=1 SV=2 | Sap130 | 1.502 | 1.445 | 1.44 | 0.55 | 0.574 | 0.564 | 0.385 | 8.13E-07 | 0.004395107 | Down |
| Q9QX47 | Protein SON OS=Mus musculus OX=10090 GN=Son PE=1 SV=2 | Son | 1.374 | 1.442 | 1.454 | 0.549 | 0.533 | 0.555 | 0.383 | 1.48E-06 | 0.007867513 | Down |
| Q8R0G9 | Nuclear pore complex protein Nup133 OS=Mus musculus OX=10090 GN=Nup133 PE=1 SV=2 | Nup133 | 1.497 | 1.422 | 1.414 | 0.531 | 0.586 | 0.542 | 0.383 | 1.06E-05 | 0.049402996 | Down |
| Q69ZQ2 | Pre-mRNA-splicing factor ISY1 homolog OS=Mus musculus OX=10090 GN=Isy1 PE=1 SV=2 | Isy1 | 1.438 | 1.43 | 1.489 | 0.551 | 0.553 | 0.556 | 0.381 | 1.93E-07 | 0.001069335 | Down |
| A2AJK6 | Chromodomain-helicase-DNA-binding protein 7 OS=Mus musculus OX=10090 GN=Chd7 PE=1 SV=1 | Chd7 | 1.445 | 1.466 | 1.406 | 0.548 | 0.525 | 0.568 | 0.38 | 3.04E-06 | 0.015641318 | Down |
| Q3TYA6 | M-phase phosphoprotein 8 OS=Mus musculus OX=10090 GN=Mphosph8 PE=1 SV=1 | Mphosph8 | 1.438 | 1.434 | 1.407 | 0.547 | 0.532 | 0.547 | 0.38 | 1.21E-07 | 0.000671109 | Down |
| Q8R3N6 | THO complex subunit 1 OS=Mus musculus OX=10090 GN=Thoc1 PE=1 SV=1 | Thoc1 | 1.438 | 1.503 | 1.455 | 0.549 | 0.544 | 0.576 | 0.38 | 1.62E-06 | 0.008587606 | Down |
| Q9R1C7 | Pre-mRNA-processing factor 40 homolog A OS=Mus musculus OX=10090 GN=Prpf40a PE=1 SV=1 | Prpf40a | 1.402 | 1.514 | 1.418 | 0.53 | 0.556 | 0.551 | 0.378 | 4.15E-06 | 0.020918344 | Down |
| Q8VE37 | Regulator of chromosome condensation OS=Mus musculus OX=10090 GN=Rcc1 PE=1 SV=1 | Rcc1 | 1.401 | 1.461 | 1.473 | 0.547 | 0.562 | 0.531 | 0.378 | 1.74E-06 | 0.009162998 | Down |
| Q64FW2 | All-trans-retinol 13,14-reductase OS=Mus musculus OX=10090 GN=Retsat PE=1 SV=3 | Retsat | 1.482 | 1.406 | 1.446 | 0.538 | 0.535 | 0.558 | 0.376 | 1.08E-06 | 0.005786153 | Down |
| O88708 | Origin recognition complex subunit 4 OS=Mus musculus OX=10090 GN=Orc4 PE=1 SV=2 | Orc4 | 1.425 | 1.476 | 1.434 | 0.55 | 0.524 | 0.551 | 0.375 | 9.71E-07 | 0.005223708 | Down |
| Q8VBZ3 | Cleft lip and palate transmembrane protein 1 homolog OS=Mus musculus OX=10090 GN=Clptm1 PE=1 SV=1 | Clptm1 | 1.45 | 1.426 | 1.436 | 0.532 | 0.532 | 0.551 | 0.375 | 1.65E-07 | 0.000918779 | Down |
| Q3TKT4 | Transcription activator BRG1 OS=Mus musculus OX=10090 GN=Smarca4 PE=1 SV=1 | Smarca4 | 1.47 | 1.464 | 1.383 | 0.524 | 0.538 | 0.551 | 0.374 | 2.28E-06 | 0.0118921 | Down |
| Q5HZG4 | Transcription initiation factor TFIID subunit 3 OS=Mus musculus OX=10090 GN=Taf3 PE=1 SV=2 | Taf3 | 1.457 | 1.479 | 1.4 | 0.558 | 0.511 | 0.551 | 0.374 | 6.62E-06 | 0.03230314 | Down |
| Q8VDP6 | CDP-diacylglycerol--inositol 3-phosphatidyltransferase OS=Mus musculus OX=10090 GN=Cdipt PE=1 SV=1 | Cdipt | 1.47 | 1.474 | 1.444 | 0.561 | 0.554 | 0.52 | 0.373 | 2.21E-06 | 0.01154907 | Down |
| Q9Z1N5 | Spliceosome RNA helicase Ddx39b OS=Mus musculus OX=10090 GN=Ddx39b PE=1 SV=1 | Ddx39b | 1.424 | 1.485 | 1.465 | 0.566 | 0.527 | 0.54 | 0.373 | 2.22E-06 | 0.011590432 | Down |
| Q9CW03 | Structural maintenance of chromosomes protein 3 OS=Mus musculus OX=10090 GN=Smc3 PE=1 SV=2 | Smc3 | 1.41 | 1.495 | 1.465 | 0.511 | 0.553 | 0.566 | 0.373 | 9.83E-06 | 0.046288891 | Down |
| A2A791 | Zinc finger MYM-type protein 4 OS=Mus musculus OX=10090 GN=Zmym4 PE=1 SV=1 | Zmym4 | 1.48 | 1.354 | 1.38 | 0.533 | 0.518 | 0.513 | 0.371 | 4.62E-06 | 0.023159299 | Down |
| Q8BHB4 | WD repeat-containing protein 3 OS=Mus musculus OX=10090 GN=Wdr3 PE=1 SV=1 | Wdr3 | 1.432 | 1.48 | 1.463 | 0.514 | 0.556 | 0.55 | 0.37 | 2.97E-06 | 0.015324852 | Down |
| Q9DC48 | Pre-mRNA-processing factor 17 OS=Mus musculus OX=10090 GN=Cdc40 PE=2 SV=1 | Cdc40 | 1.428 | 1.493 | 1.439 | 0.538 | 0.518 | 0.556 | 0.37 | 2.24E-06 | 0.011726626 | Down |
| Q6KCD5 | Nipped-B-like protein OS=Mus musculus OX=10090 GN=Nipbl PE=1 SV=1 | Nipbl | 1.401 | 1.488 | 1.455 | 0.504 | 0.556 | 0.543 | 0.369 | 8.44E-06 | 0.040372112 | Down |
| Q9CX86 | Heterogeneous nuclear ribonucleoprotein A0 OS=Mus musculus OX=10090 GN=Hnrnpa0 PE=1 SV=1 | Hnrnpa0 | 1.481 | 1.47 | 1.487 | 0.544 | 0.546 | 0.549 | 0.369 | 2.06E-09 | 1.16E-05 | Down |
| Q6ZQF0 | DNA topoisomerase 2-binding protein 1 OS=Mus musculus OX=10090 GN=Topbp1 PE=1 SV=2 | Topbp1 | 1.467 | 1.437 | 1.431 | 0.528 | 0.538 | 0.53 | 0.368 | 5.07E-08 | 0.000283476 | Down |
| Q80YR5 | Scaffold attachment factor B2 OS=Mus musculus OX=10090 GN=Safb2 PE=1 SV=2 | Safb2 | 1.365 | 1.51 | 1.466 | 0.532 | 0.536 | 0.529 | 0.368 | 4.98E-06 | 0.024841698 | Down |
| O70252 | Heme oxygenase 2 OS=Mus musculus OX=10090 GN=Hmox2 PE=1 SV=1 | Hmox2 | 1.424 | 1.468 | 1.458 | 0.496 | 0.547 | 0.553 | 0.367 | 9.62E-06 | 0.045394656 | Down |
| Q6P9R1 | ATP-dependent RNA helicase DDX51 OS=Mus musculus OX=10090 GN=Ddx51 PE=1 SV=1 | Ddx51 | 1.466 | 1.44 | 1.44 | 0.558 | 0.526 | 0.512 | 0.367 | 2.74E-06 | 0.014199113 | Down |
| Q8R2N2 | U3 small nucleolar RNA-associated protein 4 homolog OS=Mus musculus OX=10090 GN=Utp4 PE=2 SV=3 | Utp4 | 1.53 | 1.451 | 1.503 | 0.555 | 0.549 | 0.54 | 0.367 | 5.54E-07 | 0.003023353 | Down |
| Q8CJF7 | Protein ELYS OS=Mus musculus OX=10090 GN=Ahctf1 PE=1 SV=1 | Ahctf1 | 1.4 | 1.461 | 1.48 | 0.53 | 0.521 | 0.538 | 0.366 | 7.95E-07 | 0.004299338 | Down |
| Q9Z0W3 | Nuclear pore complex protein Nup160 OS=Mus musculus OX=10090 GN=Nup160 PE=1 SV=2 | Nup160 | 1.45 | 1.469 | 1.47 | 0.513 | 0.563 | 0.527 | 0.365 | 3.55E-06 | 0.018082321 | Down |
| Q99PV0 | Pre-mRNA-processing-splicing factor 8 OS=Mus musculus OX=10090 GN=Prpf8 PE=1 SV=2 | Prpf8 | 1.414 | 1.49 | 1.458 | 0.517 | 0.536 | 0.537 | 0.365 | 8.48E-07 | 0.004576095 | Down |
| Q8VDW0 | ATP-dependent RNA helicase DDX39A OS=Mus musculus OX=10090 GN=Ddx39a PE=1 SV=1 | Ddx39a | 1.432 | 1.539 | 1.435 | 0.527 | 0.537 | 0.542 | 0.365 | 2.28E-06 | 0.011909511 | Down |
| Q6NZF1 | Zinc finger CCCH domain-containing protein 11A OS=Mus musculus OX=10090 GN=Zc3h11a PE=1 SV=1 | Zc3h11a | 1.457 | 1.445 | 1.476 | 0.502 | 0.545 | 0.55 | 0.365 | 4.47E-06 | 0.022422782 | Down |
| Q8K1K4 | Centromere protein I OS=Mus musculus OX=10090 GN=Cenpi PE=2 SV=1 | Cenpi | 1.442 | 1.472 | 1.441 | 0.513 | 0.508 | 0.566 | 0.364 | 8.78E-06 | 0.041830506 | Down |
| Q8BMC4 | Nucleolar protein 9 OS=Mus musculus OX=10090 GN=Nop9 PE=1 SV=1 | Nop9 | 1.424 | 1.448 | 1.471 | 0.504 | 0.554 | 0.517 | 0.363 | 4.42E-06 | 0.022190551 | Down |
| Q9Z1M8 | Protein Red OS=Mus musculus OX=10090 GN=Ik PE=1 SV=2 | Ik | 1.45 | 1.461 | 1.501 | 0.512 | 0.56 | 0.523 | 0.362 | 3.93E-06 | 0.019871752 | Down |
| P97386 | DNA ligase 3 OS=Mus musculus OX=10090 GN=Lig3 PE=1 SV=2 | Lig3 | 1.461 | 1.466 | 1.47 | 0.508 | 0.556 | 0.528 | 0.362 | 2.64E-06 | 0.013699023 | Down |
| Q6PGG6 | Guanine nucleotide-binding protein-like 3-like protein OS=Mus musculus OX=10090 GN=Gnl3l PE=1 SV=1 | Gnl3l | 1.509 | 1.425 | 1.452 | 0.543 | 0.523 | 0.516 | 0.361 | 1.48E-06 | 0.007855226 | Down |
| Q8R4E9 | DNA replication factor Cdt1 OS=Mus musculus OX=10090 GN=Cdt1 PE=1 SV=1 | Cdt1 | 1.465 | 1.516 | 1.444 | 0.507 | 0.567 | 0.517 | 0.36 | 1.06E-05 | 0.049384148 | Down |
| D3YXK2 | Scaffold attachment factor B1 OS=Mus musculus OX=10090 GN=Safb PE=1 SV=2 | Safb | 1.451 | 1.507 | 1.394 | 0.534 | 0.518 | 0.512 | 0.359 | 2.40E-06 | 0.012512532 | Down |
| Q91YJ3 | Thymocyte nuclear protein 1 OS=Mus musculus OX=10090 GN=Thyn1 PE=1 SV=1 | Thyn1 | 1.428 | 1.502 | 1.545 | 0.513 | 0.552 | 0.542 | 0.359 | 5.59E-06 | 0.027635133 | Down |
| Q924W5 | Structural maintenance of chromosomes protein 6 OS=Mus musculus OX=10090 GN=Smc6 PE=1 SV=1 | Smc6 | 1.46 | 1.49 | 1.497 | 0.514 | 0.558 | 0.523 | 0.359 | 2.52E-06 | 0.013091165 | Down |
| Q61827 | Transcription factor MafK OS=Mus musculus OX=10090 GN=Mafk PE=1 SV=1 | Mafk | 1.415 | 1.509 | 1.516 | 0.512 | 0.52 | 0.557 | 0.358 | 7.25E-06 | 0.035066743 | Down |
| Q4VA53 | Sister chromatid cohesion protein PDS5 homolog B OS=Mus musculus OX=10090 GN=Pds5b PE=1 SV=1 | Pds5b | 1.49 | 1.47 | 1.472 | 0.506 | 0.538 | 0.542 | 0.358 | 1.30E-06 | 0.006930151 | Down |
| A2A4P0 | ATP-dependent RNA helicase DHX8 OS=Mus musculus OX=10090 GN=Dhx8 PE=2 SV=1 | Dhx8 | 1.46 | 1.494 | 1.44 | 0.496 | 0.546 | 0.532 | 0.358 | 4.71E-06 | 0.023576346 | Down |
| Q8BJ05 | Zinc finger CCCH domain-containing protein 14 OS=Mus musculus OX=10090 GN=Zc3h14 PE=1 SV=1 | Zc3h14 | 1.426 | 1.394 | 1.486 | 0.496 | 0.544 | 0.497 | 0.357 | 8.61E-06 | 0.041090709 | Down |
| Q9QXV1 | Chromobox protein homolog 8 OS=Mus musculus OX=10090 GN=Cbx8 PE=1 SV=1 | Cbx8 | 1.516 | 1.505 | 1.447 | 0.524 | 0.511 | 0.549 | 0.355 | 2.19E-06 | 0.011442028 | Down |
| Q9DBY8 | Nuclear valosin-containing protein-like OS=Mus musculus OX=10090 GN=Nvl PE=1 SV=1 | Nvl | 1.434 | 1.478 | 1.487 | 0.5 | 0.532 | 0.52 | 0.353 | 1.05E-06 | 0.005618364 | Down |
| Q9CSN1 | SNW domain-containing protein 1 OS=Mus musculus OX=10090 GN=Snw1 PE=1 SV=3 | Snw1 | 1.442 | 1.55 | 1.5 | 0.517 | 0.552 | 0.513 | 0.352 | 4.79E-06 | 0.023925276 | Down |
| Q922V4 | Pleiotropic regulator 1 OS=Mus musculus OX=10090 GN=Plrg1 PE=1 SV=1 | Plrg1 | 1.384 | 1.503 | 1.49 | 0.502 | 0.532 | 0.507 | 0.352 | 5.09E-06 | 0.025328998 | Down |
| Q91WM3 | U3 small nucleolar RNA-interacting protein 2 OS=Mus musculus OX=10090 GN=Rrp9 PE=1 SV=1 | Rrp9 | 1.475 | 1.501 | 1.428 | 0.512 | 0.503 | 0.529 | 0.351 | 9.22E-07 | 0.004969418 | Down |
| Q8CEE0 | Centrosomal protein of 57 kDa OS=Mus musculus OX=10090 GN=Cep57 PE=1 SV=2 | Cep57 | 1.494 | 1.406 | 1.564 | 0.534 | 0.512 | 0.514 | 0.349 | 6.26E-06 | 0.030685221 | Down |
| Q9QWT9 | Kinesin-like protein KIFC1 OS=Mus musculus OX=10090 GN=Kifc1 PE=1 SV=2 | Kifc1 | 1.457 | 1.497 | 1.433 | 0.502 | 0.531 | 0.493 | 0.348 | 2.10E-06 | 0.011023879 | Down |
| Q3TXT3 | SOSS complex subunit C OS=Mus musculus OX=10090 GN=Inip PE=3 SV=1 | Inip | 1.472 | 1.481 | 1.424 | 0.522 | 0.498 | 0.502 | 0.348 | 6.23E-07 | 0.003391579 | Down |
| A1L314 | Macrophage-expressed gene 1 protein OS=Mus musculus OX=10090 GN=Mpeg1 PE=1 SV=1 | Mpeg1 | 1.499 | 1.486 | 1.431 | 0.502 | 0.532 | 0.5 | 0.347 | 1.75E-06 | 0.009217354 | Down |
| Q6ZQE4 | Nuclear envelope integral membrane protein 1 OS=Mus musculus OX=10090 GN=Nemp1 PE=1 SV=2 | Nemp1 | 1.514 | 1.459 | 1.498 | 0.501 | 0.521 | 0.528 | 0.347 | 6.47E-07 | 0.00351924 | Down |
| Q0VBD2 | Protein MCM10 homolog OS=Mus musculus OX=10090 GN=Mcm10 PE=1 SV=1 | Mcm10 | 1.513 | 1.447 | 1.493 | 0.478 | 0.536 | 0.526 | 0.346 | 9.56E-06 | 0.045112533 | Down |
| P23611 | Interferon regulatory factor 8 OS=Mus musculus OX=10090 GN=Irf8 PE=1 SV=1 | Irf8 | 1.482 | 1.467 | 1.498 | 0.492 | 0.544 | 0.497 | 0.345 | 5.15E-06 | 0.025644384 | Down |
| Q9D2X5 | MAU2 chromatid cohesion factor homolog OS=Mus musculus OX=10090 GN=Mau2 PE=1 SV=3 | Mau2 | 1.488 | 1.57 | 1.443 | 0.522 | 0.51 | 0.521 | 0.345 | 2.05E-06 | 0.010774235 | Down |
| Q9R112 | Sulfide:quinone oxidoreductase, mitochondrial OS=Mus musculus OX=10090 GN=Sqor PE=1 SV=3 | Sqor | 1.531 | 1.497 | 1.442 | 0.514 | 0.542 | 0.488 | 0.345 | 6.96E-06 | 0.033857574 | Down |
| Q8K2F0 | Bromodomain-containing protein 3 OS=Mus musculus OX=10090 GN=Brd3 PE=1 SV=2 | Brd3 | 1.446 | 1.559 | 1.449 | 0.493 | 0.521 | 0.518 | 0.344 | 3.89E-06 | 0.019716942 | Down |
| Q9CU62 | Structural maintenance of chromosomes protein 1A OS=Mus musculus OX=10090 GN=Smc1a PE=1 SV=4 | Smc1a | 1.484 | 1.513 | 1.479 | 0.511 | 0.512 | 0.517 | 0.344 | 1.85E-08 | 0.000103475 | Down |
| Q8C4J7 | Transducin beta-like protein 3 OS=Mus musculus OX=10090 GN=Tbl3 PE=2 SV=1 | Tbl3 | 1.474 | 1.503 | 1.485 | 0.484 | 0.541 | 0.511 | 0.344 | 5.21E-06 | 0.025905534 | Down |
| Q5PSV9 | Mediator of DNA damage checkpoint protein 1 OS=Mus musculus OX=10090 GN=Mdc1 PE=1 SV=1 | Mdc1 | 1.449 | 1.424 | 1.494 | 0.471 | 0.528 | 0.497 | 0.343 | 7.45E-06 | 0.035957544 | Down |
| Q3U9G9 | Delta(14)-sterol reductase LBR OS=Mus musculus OX=10090 GN=Lbr PE=1 SV=2 | Lbr | 1.449 | 1.508 | 1.53 | 0.483 | 0.527 | 0.528 | 0.343 | 5.76E-06 | 0.02838063 | Down |
| P53569 | CCAAT/enhancer-binding protein zeta OS=Mus musculus OX=10090 GN=Cebpz PE=1 SV=2 | Cebpz | 1.433 | 1.483 | 1.514 | 0.502 | 0.508 | 0.507 | 0.342 | 3.33E-07 | 0.00183489 | Down |
| Q7TSH3 | Zinc finger protein 516 OS=Mus musculus OX=10090 GN=Znf516 PE=1 SV=1 | Znf516 | 1.461 | 1.496 | 1.482 | 0.484 | 0.529 | 0.504 | 0.342 | 2.25E-06 | 0.011736864 | Down |
| Q4FZC9 | Nesprin-3 OS=Mus musculus OX=10090 GN=Syne3 PE=1 SV=1 | Syne3 | 1.418 | 1.518 | 1.536 | 0.489 | 0.504 | 0.537 | 0.342 | 8.58E-06 | 0.040983644 | Down |
| Q6NZQ6 | Zinc finger protein 740 OS=Mus musculus OX=10090 GN=Znf740 PE=1 SV=1 | Znf740 | 1.527 | 1.443 | 1.377 | 0.502 | 0.49 | 0.49 | 0.341 | 4.10E-06 | 0.020718646 | Down |
| P70279 | Surfeit locus protein 6 OS=Mus musculus OX=10090 GN=Surf6 PE=1 SV=1 | Surf6 | 1.437 | 1.571 | 1.482 | 0.507 | 0.52 | 0.499 | 0.34 | 3.02E-06 | 0.015553618 | Down |
| Q8R2M2 | Deoxynucleotidyltransferase terminal-interacting protein 2 OS=Mus musculus OX=10090 GN=Dnttip2 PE=1 SV=1 | Dnttip2 | 1.446 | 1.495 | 1.5 | 0.5 | 0.49 | 0.51 | 0.338 | 3.16E-07 | 0.001741681 | Down |
| Q8BHG9 | CGG triplet repeat-binding protein 1 OS=Mus musculus OX=10090 GN=Cggbp1 PE=1 SV=1 | Cggbp1 | 1.451 | 1.567 | 1.482 | 0.493 | 0.536 | 0.481 | 0.336 | 1.07E-05 | 0.049829198 | Down |
| Q9ESX5 | H/ACA ribonucleoprotein complex subunit DKC1 OS=Mus musculus OX=10090 GN=Dkc1 PE=1 SV=4 | Dkc1 | 1.473 | 1.532 | 1.521 | 0.474 | 0.531 | 0.515 | 0.336 | 7.04E-06 | 0.034145707 | Down |
| Q3U821 | WD repeat-containing protein 75 OS=Mus musculus OX=10090 GN=Wdr75 PE=1 SV=1 | Wdr75 | 1.5 | 1.528 | 1.47 | 0.502 | 0.515 | 0.496 | 0.336 | 2.61E-07 | 0.001444986 | Down |
| Q9D0D4 | Probable dimethyladenosine transferase OS=Mus musculus OX=10090 GN=Dimt1 PE=2 SV=1 | Dimt1 | 1.48 | 1.532 | 1.52 | 0.514 | 0.51 | 0.496 | 0.335 | 2.15E-07 | 0.001193877 | Down |
| Q9JIX8 | Apoptotic chromatin condensation inducer in the nucleus OS=Mus musculus OX=10090 GN=Acin1 PE=1 SV=3 | Acin1 | 1.51 | 1.434 | 1.516 | 0.488 | 0.49 | 0.518 | 0.335 | 2.00E-06 | 0.010532334 | Down |
| Q99LJ8 | Dehydrodolichyl diphosphate synthase complex subunit Nus1 OS=Mus musculus OX=10090 GN=Nus1 PE=2 SV=1 | Nus1 | 1.46 | 1.506 | 1.528 | 0.477 | 0.49 | 0.538 | 0.335 | 9.44E-06 | 0.044609578 | Down |
| Q4VBE8 | WD repeat-containing protein 18 OS=Mus musculus OX=10090 GN=Wdr18 PE=1 SV=1 | Wdr18 | 1.531 | 1.464 | 1.48 | 0.472 | 0.506 | 0.52 | 0.335 | 4.26E-06 | 0.021451728 | Down |
| Q3URQ0 | Testis-expressed protein 10 OS=Mus musculus OX=10090 GN=Tex10 PE=1 SV=1 | Tex10 | 1.488 | 1.48 | 1.536 | 0.501 | 0.501 | 0.502 | 0.334 | 7.52E-08 | 0.000419439 | Down |
| P59110 | Sentrin-specific protease 1 OS=Mus musculus OX=10090 GN=Senp1 PE=1 SV=1 | Senp1 | 1.479 | 1.518 | 1.504 | 0.495 | 0.477 | 0.526 | 0.333 | 3.08E-06 | 0.015836788 | Down |
| Q8CH25 | SAFB-like transcription modulator OS=Mus musculus OX=10090 GN=Sltm PE=1 SV=1 | Sltm | 1.452 | 1.478 | 1.536 | 0.496 | 0.488 | 0.493 | 0.331 | 3.55E-07 | 0.001956687 | Down |
| Q80WE4 | Kinesin-like protein KIF20B OS=Mus musculus OX=10090 GN=Kif20b PE=1 SV=3 | Kif20b | 1.422 | 1.542 | 1.496 | 0.479 | 0.484 | 0.508 | 0.33 | 3.10E-06 | 0.015954477 | Down |
| Q99MY8 | Histone-lysine N-methyltransferase ASH1L OS=Mus musculus OX=10090 GN=Ash1l PE=1 SV=3 | Ash1l | 1.462 | 1.533 | 1.53 | 0.51 | 0.477 | 0.508 | 0.33 | 2.00E-06 | 0.010510583 | Down |
| Q99KK2 | N-acylneuraminate cytidylyltransferase OS=Mus musculus OX=10090 GN=Cmas PE=1 SV=2 | Cmas | 1.454 | 1.551 | 1.488 | 0.486 | 0.49 | 0.501 | 0.329 | 7.55E-07 | 0.004089567 | Down |
| P49312 | Heterogeneous nuclear ribonucleoprotein A1 OS=Mus musculus OX=10090 GN=Hnrnpa1 PE=1 SV=2 | Hnrnpa1 | 1.559 | 1.389 | 1.498 | 0.479 | 0.496 | 0.478 | 0.327 | 6.36E-06 | 0.031129606 | Down |
| Q8VI84 | Nucleolar complex protein 3 homolog OS=Mus musculus OX=10090 GN=Noc3l PE=2 SV=2 | Noc3l | 1.392 | 1.501 | 1.548 | 0.48 | 0.503 | 0.463 | 0.326 | 9.28E-06 | 0.043947414 | Down |
| Q61464 | Zinc finger protein 638 OS=Mus musculus OX=10090 GN=Znf638 PE=1 SV=2 | Znf638 | 1.516 | 1.507 | 1.486 | 0.459 | 0.518 | 0.492 | 0.326 | 5.98E-06 | 0.029408085 | Down |
| Q6NZQ2 | Probable ATP-dependent RNA helicase DDX31 OS=Mus musculus OX=10090 GN=Ddx31 PE=2 SV=2 | Ddx31 | 1.457 | 1.462 | 1.555 | 0.489 | 0.476 | 0.489 | 0.325 | 1.05E-06 | 0.005618364 | Down |
| Q8CI11 | Guanine nucleotide-binding protein-like 3 OS=Mus musculus OX=10090 GN=Gnl3 PE=1 SV=2 | Gnl3 | 1.471 | 1.473 | 1.518 | 0.481 | 0.482 | 0.489 | 0.325 | 6.61E-08 | 0.000368727 | Down |
| Q6PDG5 | SWI/SNF complex subunit SMARCC2 OS=Mus musculus OX=10090 GN=Smarcc2 PE=1 SV=2 | Smarcc2 | 1.481 | 1.543 | 1.452 | 0.48 | 0.494 | 0.482 | 0.325 | 6.06E-07 | 0.003297765 | Down |
| Q9QZQ8 | Core histone macro-H2A.1 OS=Mus musculus OX=10090 GN=Macroh2a1 PE=1 SV=3 | Macroh2a1 | 1.481 | 1.521 | 1.563 | 0.476 | 0.514 | 0.49 | 0.324 | 2.05E-06 | 0.01076234 | Down |
| Q0VEE6 | Zinc finger protein 800 OS=Mus musculus OX=10090 GN=Znf800 PE=1 SV=1 | Znf800 | 1.475 | 1.526 | 1.464 | 0.48 | 0.493 | 0.473 | 0.324 | 3.56E-07 | 0.001959442 | Down |
| O35638 | Cohesin subunit SA-2 OS=Mus musculus OX=10090 GN=Stag2 PE=1 SV=3 | Stag2 | 1.483 | 1.563 | 1.527 | 0.474 | 0.476 | 0.529 | 0.323 | 8.45E-06 | 0.04041746 | Down |
| Q9R0Q4 | Mortality factor 4-like protein 2 OS=Mus musculus OX=10090 GN=Morf4l2 PE=1 SV=1 | Morf4l2 | 1.453 | 1.594 | 1.517 | 0.479 | 0.495 | 0.492 | 0.321 | 2.41E-06 | 0.012533757 | Down |
| Q8CG46 | Structural maintenance of chromosomes protein 5 OS=Mus musculus OX=10090 GN=Smc5 PE=1 SV=1 | Smc5 | 1.466 | 1.542 | 1.514 | 0.464 | 0.492 | 0.491 | 0.32 | 1.22E-06 | 0.006526695 | Down |
| Q08288 | Cell growth-regulating nucleolar protein OS=Mus musculus OX=10090 GN=Lyar PE=1 SV=2 | Lyar | 1.441 | 1.51 | 1.542 | 0.476 | 0.494 | 0.462 | 0.319 | 2.11E-06 | 0.011040281 | Down |
| E9Q784 | Zinc finger CCCH domain-containing protein 13 OS=Mus musculus OX=10090 GN=Zc3h13 PE=1 SV=1 | Zc3h13 | 1.502 | 1.509 | 1.542 | 0.46 | 0.506 | 0.488 | 0.319 | 2.46E-06 | 0.012794356 | Down |
| Q6ZPV2 | Chromatin-remodeling ATPase INO80 OS=Mus musculus OX=10090 GN=Ino80 PE=1 SV=2 | Ino80 | 1.508 | 1.493 | 1.544 | 0.445 | 0.482 | 0.512 | 0.317 | 1.03E-05 | 0.048443802 | Down |
| Q91YU8 | Suppressor of SWI4 1 homolog OS=Mus musculus OX=10090 GN=Ppan PE=1 SV=2 | Ppan | 1.464 | 1.561 | 1.537 | 0.499 | 0.484 | 0.462 | 0.317 | 2.62E-06 | 0.013584674 | Down |
| Q8BRH4 | Histone-lysine N-methyltransferase 2C OS=Mus musculus OX=10090 GN=Kmt2c PE=1 SV=2 | Kmt2c | 1.423 | 1.578 | 1.564 | 0.479 | 0.476 | 0.492 | 0.317 | 4.92E-06 | 0.024561838 | Down |
| Q9ER62 | Tumor necrosis factor receptor superfamily member 22 OS=Mus musculus OX=10090 GN=Tnfrsf22 PE=2 SV=3 | Tnfrsf22 | 1.569 | 1.542 | 1.469 | 0.513 | 0.462 | 0.475 | 0.317 | 6.37E-06 | 0.031178147 | Down |
| Q9QYL7 | Activator of basal transcription 1 OS=Mus musculus OX=10090 GN=Abt1 PE=2 SV=1 | Abt1 | 1.44 | 1.534 | 1.542 | 0.495 | 0.479 | 0.451 | 0.316 | 5.07E-06 | 0.025228481 | Down |
| Q99ME9 | Nucleolar GTP-binding protein 1 OS=Mus musculus OX=10090 GN=Gtpbp4 PE=1 SV=3 | Gtpbp4 | 1.481 | 1.503 | 1.537 | 0.47 | 0.491 | 0.466 | 0.316 | 4.85E-07 | 0.002656089 | Down |
| F8VPZ9 | BRD4-interacting chromatin-remodeling complex-associated protein OS=Mus musculus OX=10090 GN=Bicra PE=1 SV=1 | Bicra | 1.479 | 1.551 | 1.517 | 0.5 | 0.483 | 0.454 | 0.316 | 3.29E-06 | 0.016840487 | Down |
| Q9QXW0 | F-box/LRR-repeat protein 6 OS=Mus musculus OX=10090 GN=Fbxl6 PE=2 SV=2 | Fbxl6 | 1.494 | 1.521 | 1.56 | 0.487 | 0.458 | 0.501 | 0.316 | 2.50E-06 | 0.012982054 | Down |
| Q8VDD9 | PH-interacting protein OS=Mus musculus OX=10090 GN=Phip PE=1 SV=2 | Phip | 1.476 | 1.537 | 1.542 | 0.478 | 0.501 | 0.451 | 0.314 | 4.16E-06 | 0.020996984 | Down |
| Q3UFM5 | Nucleolar MIF4G domain-containing protein 1 OS=Mus musculus OX=10090 GN=Nom1 PE=1 SV=2 | Nom1 | 1.527 | 1.584 | 1.496 | 0.483 | 0.484 | 0.475 | 0.313 | 3.26E-07 | 0.00179983 | Down |
| Q8C6L5 | Cyclic GMP-AMP synthase OS=Mus musculus OX=10090 GN=Cgas PE=1 SV=1 | Cgas | 1.469 | 1.508 | 1.51 | 0.467 | 0.5 | 0.436 | 0.313 | 8.76E-06 | 0.041737482 | Down |
| Q9D0I8 | mRNA turnover protein 4 homolog OS=Mus musculus OX=10090 GN=Mrto4 PE=1 SV=1 | Mrto4 | 1.539 | 1.553 | 1.517 | 0.478 | 0.487 | 0.471 | 0.312 | 6.36E-08 | 0.000355054 | Down |
| Q8VCG3 | WD repeat-containing protein 74 OS=Mus musculus OX=10090 GN=Wdr74 PE=2 SV=1 | Wdr74 | 1.443 | 1.559 | 1.568 | 0.47 | 0.503 | 0.454 | 0.312 | 8.54E-06 | 0.040804943 | Down |
| Q9WTU0 | Lysine-specific demethylase PHF2 OS=Mus musculus OX=10090 GN=Phf2 PE=1 SV=2 | Phf2 | 1.466 | 1.494 | 1.492 | 0.464 | 0.455 | 0.464 | 0.311 | 2.04E-08 | 0.000114463 | Down |
| Q9JK30 | Origin recognition complex subunit 3 OS=Mus musculus OX=10090 GN=Orc3 PE=1 SV=1 | Orc3 | 1.486 | 1.58 | 1.514 | 0.439 | 0.499 | 0.481 | 0.31 | 9.96E-06 | 0.046835541 | Down |
| Q8R3N1 | Nucleolar protein 14 OS=Mus musculus OX=10090 GN=Nop14 PE=1 SV=2 | Nop14 | 1.502 | 1.537 | 1.509 | 0.446 | 0.498 | 0.468 | 0.31 | 3.63E-06 | 0.018461722 | Down |
| Q8R081 | Heterogeneous nuclear ribonucleoprotein L OS=Mus musculus OX=10090 GN=Hnrnpl PE=1 SV=2 | Hnrnpl | 1.426 | 1.506 | 1.542 | 0.448 | 0.48 | 0.458 | 0.31 | 2.86E-06 | 0.014764043 | Down |
| Q9Z1N2 | Origin recognition complex subunit 1 OS=Mus musculus OX=10090 GN=Orc1 PE=1 SV=2 | Orc1 | 1.515 | 1.554 | 1.508 | 0.451 | 0.458 | 0.505 | 0.309 | 5.61E-06 | 0.027729686 | Down |
| Q8BKT7 | THO complex subunit 5 homolog OS=Mus musculus OX=10090 GN=Thoc5 PE=1 SV=2 | Thoc5 | 1.495 | 1.56 | 1.575 | 0.457 | 0.495 | 0.478 | 0.309 | 1.96E-06 | 0.010317332 | Down |
| Q4VBD9 | GDNF-inducible zinc finger protein 1 OS=Mus musculus OX=10090 GN=Gzf1 PE=1 SV=2 | Gzf1 | 1.509 | 1.611 | 1.484 | 0.444 | 0.47 | 0.501 | 0.307 | 1.04E-05 | 0.048786192 | Down |
| Q3UHX9 | Putative methyltransferase C9orf114 homolog OS=Mus musculus OX=10090 GN=Spout1 PE=1 SV=1 | Spout1 | 1.526 | 1.554 | 1.496 | 0.473 | 0.471 | 0.46 | 0.307 | 1.18E-07 | 0.000656879 | Down |
| Q8BH07 | ADP-ribosylation factor-like protein 6-interacting protein 6 OS=Mus musculus OX=10090 GN=Arl6ip6 PE=1 SV=1 | Arl6ip6 | 1.657 | 1.516 | 1.446 | 0.459 | 0.476 | 0.48 | 0.306 | 9.70E-06 | 0.045722547 | Down |
| Q9D7Z3 | Nucleolar protein 7 OS=Mus musculus OX=10090 GN=Nol7 PE=1 SV=1 | Nol7 | 1.482 | 1.571 | 1.495 | 0.462 | 0.473 | 0.452 | 0.305 | 7.58E-07 | 0.004104083 | Down |
| Q91ZW3 | SWI/SNF-related matrix-associated actin-dependent regulator of chromatin subfamily A member 5 OS=Mus musculus OX=10090 GN=Smarca5 PE=1 SV=1 | Smarca5 | 1.51 | 1.53 | 1.547 | 0.457 | 0.469 | 0.458 | 0.302 | 4.04E-08 | 0.000225949 | Down |
| Q9CYH6 | Ribosome biogenesis regulatory protein homolog OS=Mus musculus OX=10090 GN=Rrs1 PE=1 SV=1 | Rrs1 | 1.556 | 1.463 | 1.482 | 0.455 | 0.46 | 0.438 | 0.301 | 9.41E-07 | 0.005069194 | Down |
| Q8R0K4 | Coiled-coil domain-containing protein 137 OS=Mus musculus OX=10090 GN=Ccdc137 PE=2 SV=1 | Ccdc137 | 1.588 | 1.504 | 1.603 | 0.483 | 0.461 | 0.465 | 0.3 | 1.03E-06 | 0.005515282 | Down |
| Q9Z280 | Phospholipase D1 OS=Mus musculus OX=10090 GN=Pld1 PE=1 SV=1 | Pld1 | 1.581 | 1.564 | 1.49 | 0.45 | 0.489 | 0.448 | 0.299 | 3.68E-06 | 0.018692627 | Down |
| Q60862 | Origin recognition complex subunit 2 OS=Mus musculus OX=10090 GN=Orc2 PE=1 SV=1 | Orc2 | 1.6 | 1.459 | 1.5 | 0.463 | 0.446 | 0.455 | 0.299 | 2.11E-06 | 0.011042997 | Down |
| Q9CRY7 | Lysophospholipase D GDPD1 OS=Mus musculus OX=10090 GN=Gdpd1 PE=1 SV=1 | Gdpd1 | 1.467 | 1.58 | 1.546 | 0.424 | 0.475 | 0.476 | 0.299 | 1.06E-05 | 0.049510076 | Down |
| Q99PP2 | Zinc finger protein 318 OS=Mus musculus OX=10090 GN=Znf318 PE=1 SV=3 | Znf318 | 1.524 | 1.535 | 1.575 | 0.43 | 0.471 | 0.481 | 0.298 | 4.57E-06 | 0.022897133 | Down |
| P14733 | Lamin-B1 OS=Mus musculus OX=10090 GN=Lmnb1 PE=1 SV=3 | Lmnb1 | 1.492 | 1.581 | 1.522 | 0.438 | 0.469 | 0.457 | 0.297 | 1.29E-06 | 0.006897301 | Down |
| Q8C7V3 | U3 small nucleolar RNA-associated protein 15 homolog OS=Mus musculus OX=10090 GN=Utp15 PE=1 SV=1 | Utp15 | 1.493 | 1.537 | 1.589 | 0.426 | 0.481 | 0.466 | 0.297 | 7.43E-06 | 0.035875027 | Down |
| Q8VIJ6 | Splicing factor, proline- and glutamine-rich OS=Mus musculus OX=10090 GN=Sfpq PE=1 SV=1 | Sfpq | 1.52 | 1.538 | 1.508 | 0.422 | 0.465 | 0.465 | 0.296 | 3.16E-06 | 0.016206986 | Down |
| Q9D1J3 | SAP domain-containing ribonucleoprotein OS=Mus musculus OX=10090 GN=Sarnp PE=1 SV=3 | Sarnp | 1.509 | 1.594 | 1.522 | 0.473 | 0.441 | 0.454 | 0.296 | 1.34E-06 | 0.00713716 | Down |
| Q499D0 | Forkhead box protein N3 OS=Mus musculus OX=10090 GN=Foxn3 PE=1 SV=1 | Foxn3 | 1.581 | 1.582 | 1.491 | 0.46 | 0.462 | 0.448 | 0.294 | 6.13E-07 | 0.003337373 | Down |
| Q8K0L9 | Zinc finger and BTB domain-containing protein 20 OS=Mus musculus OX=10090 GN=Zbtb20 PE=1 SV=1 | Zbtb20 | 1.508 | 1.623 | 1.516 | 0.446 | 0.463 | 0.458 | 0.294 | 1.25E-06 | 0.006663799 | Down |
| Q80X41 | Serine/threonine-protein kinase VRK1 OS=Mus musculus OX=10090 GN=Vrk1 PE=1 SV=2 | Vrk1 | 1.549 | 1.521 | 1.544 | 0.459 | 0.455 | 0.435 | 0.292 | 2.49E-07 | 0.001378552 | Down |
| Q8BG81 | Polymerase delta-interacting protein 3 OS=Mus musculus OX=10090 GN=Poldip3 PE=1 SV=1 | Poldip3 | 1.524 | 1.49 | 1.59 | 0.418 | 0.456 | 0.456 | 0.289 | 3.63E-06 | 0.018481804 | Down |
| Q8CIB9 | N-acetyltransferase ESCO2 OS=Mus musculus OX=10090 GN=Esco2 PE=2 SV=3 | Esco2 | 1.545 | 1.528 | 1.559 | 0.449 | 0.411 | 0.48 | 0.289 | 1.05E-05 | 0.049240373 | Down |
| Q3TEA8 | Heterochromatin protein 1-binding protein 3 OS=Mus musculus OX=10090 GN=Hp1bp3 PE=1 SV=1 | Hp1bp3 | 1.516 | 1.592 | 1.552 | 0.424 | 0.479 | 0.437 | 0.288 | 5.87E-06 | 0.028917886 | Down |
| P11103 | Poly [ADP-ribose] polymerase 1 OS=Mus musculus OX=10090 GN=Parp1 PE=1 SV=3 | Parp1 | 1.525 | 1.559 | 1.548 | 0.414 | 0.452 | 0.463 | 0.287 | 3.51E-06 | 0.017925257 | Down |
| Q9EQ61 | Pescadillo homolog OS=Mus musculus OX=10090 GN=Pes1 PE=1 SV=1 | Pes1 | 1.473 | 1.63 | 1.537 | 0.418 | 0.448 | 0.461 | 0.286 | 7.07E-06 | 0.034296619 | Down |
| Q6P3Y5 | Zinc finger protein 280C OS=Mus musculus OX=10090 GN=Znf280c PE=1 SV=1 | Znf280c | 1.486 | 1.618 | 1.536 | 0.416 | 0.454 | 0.448 | 0.284 | 4.35E-06 | 0.021890357 | Down |
| Q921F2 | TAR DNA-binding protein 43 OS=Mus musculus OX=10090 GN=Tardbp PE=1 SV=1 | Tardbp | 1.544 | 1.568 | 1.543 | 0.424 | 0.47 | 0.43 | 0.284 | 2.72E-06 | 0.014058374 | Down |
| D0QMC3 | Myeloid cell nuclear differentiation antigen-like protein OS=Mus musculus OX=10090 GN=Mndal PE=1 SV=1 | Mndal | 1.554 | 1.606 | 1.538 | 0.415 | 0.444 | 0.47 | 0.283 | 5.01E-06 | 0.024974451 | Down |
| Q8BVY0 | Ribosomal L1 domain-containing protein 1 OS=Mus musculus OX=10090 GN=Rsl1d1 PE=1 SV=1 | Rsl1d1 | 1.488 | 1.53 | 1.592 | 0.454 | 0.431 | 0.42 | 0.283 | 1.94E-06 | 0.010222277 | Down |
| Q3U1G5 | Interferon-stimulated 20 kDa exonuclease-like 2 OS=Mus musculus OX=10090 GN=Isg20l2 PE=1 SV=2 | Isg20l2 | 1.544 | 1.518 | 1.604 | 0.438 | 0.45 | 0.432 | 0.283 | 3.95E-07 | 0.002171109 | Down |
| Q8BRG8 | Transmembrane protein 209 OS=Mus musculus OX=10090 GN=Tmem209 PE=2 SV=1 | Tmem209 | 1.531 | 1.558 | 1.543 | 0.445 | 0.448 | 0.42 | 0.283 | 4.69E-07 | 0.002574898 | Down |
| Q8BHS3 | Pre-mRNA-splicing factor RBM22 OS=Mus musculus OX=10090 GN=Rbm22 PE=1 SV=1 | Rbm22 | 1.602 | 1.551 | 1.541 | 0.408 | 0.469 | 0.441 | 0.281 | 7.13E-06 | 0.034565314 | Down |
| Q8R3C6 | Probable RNA-binding protein 19 OS=Mus musculus OX=10090 GN=Rbm19 PE=1 SV=1 | Rbm19 | 1.53 | 1.552 | 1.558 | 0.402 | 0.436 | 0.456 | 0.279 | 4.32E-06 | 0.021762284 | Down |
| Q9JJ89 | Coiled-coil domain-containing protein 86 OS=Mus musculus OX=10090 GN=Ccdc86 PE=1 SV=2 | Ccdc86 | 1.578 | 1.629 | 1.487 | 0.436 | 0.406 | 0.462 | 0.278 | 9.83E-06 | 0.046286753 | Down |
| Q8K4L0 | ATP-dependent RNA helicase DDX54 OS=Mus musculus OX=10090 GN=Ddx54 PE=1 SV=1 | Ddx54 | 1.5 | 1.647 | 1.526 | 0.413 | 0.446 | 0.415 | 0.273 | 4.36E-06 | 0.02193199 | Down |
| Q9EPA7 | Nicotinamide/nicotinic acid mononucleotide adenylyltransferase 1 OS=Mus musculus OX=10090 GN=Nmnat1 PE=1 SV=2 | Nmnat1 | 1.694 | 1.509 | 1.555 | 0.449 | 0.403 | 0.426 | 0.269 | 9.43E-06 | 0.044573852 | Down |
| Q5XG71 | Small subunit processome component 20 homolog OS=Mus musculus OX=10090 GN=Utp20 PE=1 SV=2 | Utp20 | 1.56 | 1.578 | 1.601 | 0.41 | 0.449 | 0.413 | 0.268 | 1.64E-06 | 0.008645998 | Down |
| Q6TEK5 | Vitamin K epoxide reductase complex subunit 1-like protein 1 OS=Mus musculus OX=10090 GN=Vkorc1l1 PE=1 SV=1 | Vkorc1l1 | 1.516 | 1.712 | 1.51 | 0.406 | 0.436 | 0.429 | 0.268 | 9.33E-06 | 0.044162966 | Down |
| Q8BKS9 | Pumilio homolog 3 OS=Mus musculus OX=10090 GN=Pum3 PE=1 SV=2 | Pum3 | 1.531 | 1.602 | 1.578 | 0.382 | 0.436 | 0.441 | 0.267 | 1.03E-05 | 0.048420881 | Down |
| O35368 | Interferon-activable protein 203 OS=Mus musculus OX=10090 GN=Ifi203 PE=1 SV=1 | Ifi203 | 1.525 | 1.623 | 1.546 | 0.383 | 0.43 | 0.432 | 0.265 | 6.97E-06 | 0.033857574 | Down |
| Q9CXS4 | Centromere protein V OS=Mus musculus OX=10090 GN=Cenpv PE=1 SV=2 | Cenpv | 1.552 | 1.506 | 1.556 | 0.39 | 0.385 | 0.438 | 0.263 | 5.98E-06 | 0.029399115 | Down |
| Q91YK2 | Ribosomal RNA processing protein 1 homolog B OS=Mus musculus OX=10090 GN=Rrp1b PE=1 SV=2 | Rrp1b | 1.506 | 1.563 | 1.632 | 0.378 | 0.434 | 0.413 | 0.261 | 8.55E-06 | 0.040855623 | Down |
| Q9D903 | Probable rRNA-processing protein EBP2 OS=Mus musculus OX=10090 GN=Ebna1bp2 PE=2 SV=1 | Ebna1bp2 | 1.472 | 1.599 | 1.595 | 0.377 | 0.42 | 0.402 | 0.257 | 5.18E-06 | 0.025779671 | Down |
| Q9JI13 | Something about silencing protein 10 OS=Mus musculus OX=10090 GN=Utp3 PE=1 SV=1 | Utp3 | 1.566 | 1.67 | 1.494 | 0.384 | 0.423 | 0.397 | 0.255 | 5.80E-06 | 0.028601521 | Down |
| Q9QZ67 | Protein phosphatase 1D OS=Mus musculus OX=10090 GN=Ppm1d PE=2 SV=2 | Ppm1d | 1.634 | 1.506 | 1.656 | 0.396 | 0.441 | 0.387 | 0.255 | 1.07E-05 | 0.049807624 | Down |
| Q8R1J3 | Zinc finger CCHC domain-containing protein 9 OS=Mus musculus OX=10090 GN=Zcchc9 PE=2 SV=2 | Zcchc9 | 1.567 | 1.674 | 1.498 | 0.388 | 0.406 | 0.405 | 0.253 | 2.65E-06 | 0.013755226 | Down |
| Q99P91 | Transmembrane glycoprotein NMB OS=Mus musculus OX=10090 GN=Gpnmb PE=1 SV=2 | Gpnmb | 1.601 | 1.683 | 1.546 | 0.376 | 0.394 | 0.432 | 0.249 | 8.17E-06 | 0.039157915 | Down |
| Q5SSI6 | U3 small nucleolar RNA-associated protein 18 homolog OS=Mus musculus OX=10090 GN=Utp18 PE=1 SV=1 | Utp18 | 1.607 | 1.608 | 1.609 | 0.381 | 0.408 | 0.407 | 0.248 | 4.00E-07 | 0.00219897 | Down |
| Q8R3Y5 | Uncharacterized protein C19orf47 homolog OS=Mus musculus OX=10090 PE=1 SV=2 | -- | 1.568 | 1.646 | 1.534 | 0.375 | 0.39 | 0.406 | 0.247 | 1.43E-06 | 0.007617719 | Down |
| Q9DBE9 | pre-rRNA 2'-O-ribose RNA methyltransferase FTSJ3 OS=Mus musculus OX=10090 GN=Ftsj3 PE=1 SV=1 | Ftsj3 | 1.552 | 1.618 | 1.591 | 0.373 | 0.423 | 0.382 | 0.247 | 4.17E-06 | 0.021036395 | Down |
| Q8C5L7 | RNA-binding protein 34 OS=Mus musculus OX=10090 GN=Rbm34 PE=1 SV=2 | Rbm34 | 1.58 | 1.658 | 1.526 | 0.36 | 0.397 | 0.406 | 0.244 | 5.67E-06 | 0.028005571 | Down |
| Q922G2 | Protein FAM76A OS=Mus musculus OX=10090 GN=Fam76a PE=2 SV=1 | Fam76a | 1.481 | 1.591 | 1.605 | 0.371 | 0.381 | 0.388 | 0.244 | 1.01E-06 | 0.005424773 | Down |
| Q80W00 | Serine/threonine-protein phosphatase 1 regulatory subunit 10 OS=Mus musculus OX=10090 GN=Ppp1r10 PE=1 SV=1 | Ppp1r10 | 1.404 | 1.559 | 1.571 | 0.352 | 0.386 | 0.364 | 0.243 | 6.21E-06 | 0.030424312 | Down |
| O54864 | Histone-lysine N-methyltransferase SUV39H1 OS=Mus musculus OX=10090 GN=Suv39h1 PE=1 SV=1 | Suv39h1 | 1.636 | 1.542 | 1.668 | 0.376 | 0.416 | 0.384 | 0.243 | 3.37E-06 | 0.017223976 | Down |
| O55187 | E3 SUMO-protein ligase CBX4 OS=Mus musculus OX=10090 GN=Cbx4 PE=1 SV=2 | Cbx4 | 1.62 | 1.662 | 1.578 | 0.376 | 0.429 | 0.368 | 0.241 | 9.22E-06 | 0.043726978 | Down |
| Q61656 | Probable ATP-dependent RNA helicase DDX5 OS=Mus musculus OX=10090 GN=Ddx5 PE=1 SV=2 | Ddx5 | 1.618 | 1.626 | 1.637 | 0.36 | 0.406 | 0.408 | 0.241 | 4.10E-06 | 0.020700621 | Down |
| Q8C6B9 | Active regulator of SIRT1 OS=Mus musculus OX=10090 GN=Rps19bp1 PE=1 SV=1 | Rps19bp1 | 1.63 | 1.576 | 1.61 | 0.376 | 0.383 | 0.394 | 0.239 | 1.14E-07 | 0.00063462 | Down |
| Q61029 | Lamina-associated polypeptide 2, isoforms beta/delta/epsilon/gamma OS=Mus musculus OX=10090 GN=Tmpo PE=1 SV=4 | Tmpo | 1.46 | 1.636 | 1.617 | 0.351 | 0.381 | 0.389 | 0.238 | 7.36E-06 | 0.035580958 | Down |
| P13864 | DNA (cytosine-5)-methyltransferase 1 OS=Mus musculus OX=10090 GN=Dnmt1 PE=1 SV=5 | Dnmt1 | 1.565 | 1.644 | 1.612 | 0.38 | 0.38 | 0.384 | 0.237 | 6.60E-08 | 0.000368458 | Down |
| E9PVX6 | Proliferation marker protein Ki-67 OS=Mus musculus OX=10090 GN=Mki67 PE=1 SV=1 | Mki67 | 1.528 | 1.641 | 1.642 | 0.354 | 0.388 | 0.391 | 0.236 | 3.45E-06 | 0.017613635 | Down |
| Q9CTH6 | rRNA-processing protein FCF1 homolog OS=Mus musculus OX=10090 GN=Fcf1 PE=2 SV=2 | Fcf1 | 1.64 | 1.551 | 1.678 | 0.386 | 0.402 | 0.361 | 0.236 | 3.21E-06 | 0.016462265 | Down |
| Q80TG1 | KAT8 regulatory NSL complex subunit 1 OS=Mus musculus OX=10090 GN=Kansl1 PE=1 SV=1 | Kansl1 | 1.619 | 1.656 | 1.602 | 0.362 | 0.394 | 0.384 | 0.234 | 7.03E-07 | 0.003814028 | Down |
| Q9Z277 | Tyrosine-protein kinase BAZ1B OS=Mus musculus OX=10090 GN=Baz1b PE=1 SV=2 | Baz1b | 1.529 | 1.642 | 1.637 | 0.358 | 0.4 | 0.369 | 0.234 | 3.61E-06 | 0.018377995 | Down |
| Q99LH1 | Nucleolar GTP-binding protein 2 OS=Mus musculus OX=10090 GN=Gnl2 PE=1 SV=2 | Gnl2 | 1.63 | 1.629 | 1.587 | 0.371 | 0.362 | 0.394 | 0.233 | 6.67E-07 | 0.003623953 | Down |
| Q02780 | Nuclear factor 1 A-type OS=Mus musculus OX=10090 GN=Nfia PE=1 SV=1 | Nfia | 1.645 | 1.585 | 1.662 | 0.398 | 0.362 | 0.373 | 0.232 | 1.28E-06 | 0.00682752 | Down |
| Q9DCC3 | Coiled-coil domain-containing protein 107 OS=Mus musculus OX=10090 GN=Ccdc107 PE=2 SV=1 | Ccdc107 | 1.525 | 1.747 | 1.602 | 0.373 | 0.398 | 0.361 | 0.232 | 7.57E-06 | 0.036483303 | Down |
| Q9DCA5 | Ribosome biogenesis protein BRX1 homolog OS=Mus musculus OX=10090 GN=Brix1 PE=1 SV=3 | Brix1 | 1.57 | 1.663 | 1.56 | 0.376 | 0.378 | 0.346 | 0.23 | 1.94E-06 | 0.010191957 | Down |
| Q8BG15 | CTD small phosphatase-like protein 2 OS=Mus musculus OX=10090 GN=Ctdspl2 PE=1 SV=1 | Ctdspl2 | 1.668 | 1.638 | 1.62 | 0.378 | 0.374 | 0.345 | 0.223 | 9.60E-07 | 0.005166298 | Down |
| A6PWY4 | WD repeat-containing protein 76 OS=Mus musculus OX=10090 GN=Wdr76 PE=1 SV=1 | Wdr76 | 1.532 | 1.691 | 1.674 | 0.336 | 0.381 | 0.37 | 0.222 | 6.82E-06 | 0.03320249 | Down |
| Q9JIK5 | Nucleolar RNA helicase 2 OS=Mus musculus OX=10090 GN=Ddx21 PE=1 SV=3 | Ddx21 | 1.574 | 1.632 | 1.67 | 0.338 | 0.378 | 0.362 | 0.221 | 2.12E-06 | 0.01109484 | Down |
| Q8CGC6 | RNA-binding protein 28 OS=Mus musculus OX=10090 GN=Rbm28 PE=1 SV=4 | Rbm28 | 1.58 | 1.715 | 1.669 | 0.359 | 0.342 | 0.378 | 0.217 | 2.21E-06 | 0.011545998 | Down |
| P60762 | Mortality factor 4-like protein 1 OS=Mus musculus OX=10090 GN=Morf4l1 PE=1 SV=2 | Morf4l1 | 1.606 | 1.756 | 1.584 | 0.329 | 0.349 | 0.378 | 0.214 | 7.41E-06 | 0.035812223 | Down |
| Q3UFY0 | Ribosomal RNA processing protein 36 homolog OS=Mus musculus OX=10090 GN=Rrp36 PE=1 SV=1 | Rrp36 | 1.599 | 1.611 | 1.674 | 0.333 | 0.361 | 0.346 | 0.213 | 5.80E-07 | 0.003164821 | Down |
| Q9WU62 | Inner centromere protein OS=Mus musculus OX=10090 GN=Incenp PE=1 SV=2 | Incenp | 1.557 | 1.637 | 1.69 | 0.312 | 0.359 | 0.365 | 0.212 | 9.50E-06 | 0.044877993 | Down |
| Q99K43 | Protein regulator of cytokinesis 1 OS=Mus musculus OX=10090 GN=Prc1 PE=1 SV=2 | Prc1 | 1.51 | 1.687 | 1.705 | 0.335 | 0.339 | 0.361 | 0.211 | 4.28E-06 | 0.0215639 | Down |
| Q8BVK9 | Sp110 nuclear body protein OS=Mus musculus OX=10090 GN=Sp110 PE=1 SV=1 | Sp110 | 1.52 | 1.686 | 1.63 | 0.342 | 0.342 | 0.329 | 0.209 | 1.21E-06 | 0.006472261 | Down |
| P55065 | Phospholipid transfer protein OS=Mus musculus OX=10090 GN=Pltp PE=1 SV=1 | Pltp | 1.691 | 1.58 | 1.616 | 0.318 | 0.347 | 0.318 | 0.201 | 1.40E-06 | 0.007447697 | Down |
| Q7TSY8 | Shugoshin 2 OS=Mus musculus OX=10090 GN=Sgo2 PE=1 SV=1 | Sgo2 | 1.58 | 1.634 | 1.688 | 0.342 | 0.316 | 0.309 | 0.197 | 1.48E-06 | 0.007858527 | Down |
| O70201 | Baculoviral IAP repeat-containing protein 5 OS=Mus musculus OX=10090 GN=Birc5 PE=1 SV=1 | Birc5 | 1.564 | 1.802 | 1.669 | 0.348 | 0.314 | 0.313 | 0.194 | 6.85E-06 | 0.033329444 | Down |
| Q01237 | 3-hydroxy-3-methylglutaryl-coenzyme A reductase OS=Mus musculus OX=10090 GN=Hmgcr PE=1 SV=3 | Hmgcr | 1.592 | 1.664 | 1.699 | 0.286 | 0.338 | 0.323 | 0.191 | 6.44E-06 | 0.031502858 | Down |
| A2APB8 | Targeting protein for Xklp2 OS=Mus musculus OX=10090 GN=Tpx2 PE=1 SV=1 | Tpx2 | 1.591 | 1.746 | 1.721 | 0.307 | 0.301 | 0.331 | 0.186 | 2.08E-06 | 0.010900231 | Down |
| P30415 | NK-tumor recognition protein OS=Mus musculus OX=10090 GN=Nktr PE=1 SV=4 | Nktr | 1.823 | 1.57 | 1.728 | 0.291 | 0.303 | 0.316 | 0.178 | 4.12E-06 | 0.020801102 | Down |
| Q9CR47 | Ribosome biogenesis protein NSA2 homolog OS=Mus musculus OX=10090 GN=Nsa2 PE=2 SV=1 | Nsa2 | 1.698 | 1.756 | 1.684 | 0.27 | 0.322 | 0.317 | 0.177 | 7.30E-06 | 0.035288624 | Down |
| Q8VDF2 | E3 ubiquitin-protein ligase UHRF1 OS=Mus musculus OX=10090 GN=Uhrf1 PE=1 SV=2 | Uhrf1 | 1.573 | 1.723 | 1.598 | 0.31 | 0.258 | 0.279 | 0.173 | 8.20E-06 | 0.03927775 | Down |
| Q922M5 | Cell division cycle-associated 7-like protein OS=Mus musculus OX=10090 GN=Cdca7l PE=1 SV=1 | Cdca7l | 1.572 | 1.784 | 1.788 | 0.265 | 0.292 | 0.3 | 0.167 | 6.03E-06 | 0.029624246 | Down |
| P52927 | High mobility group protein HMGI-C OS=Mus musculus OX=10090 GN=Hmga2 PE=1 SV=1 | Hmga2 | 1.552 | 1.666 | 1.843 | 0.302 | 0.27 | 0.262 | 0.165 | 1.07E-05 | 0.049833441 | Down |
| Q01320 | DNA topoisomerase 2-alpha OS=Mus musculus OX=10090 GN=Top2a PE=1 SV=2 | Top2a | 1.623 | 1.719 | 1.688 | 0.257 | 0.258 | 0.3 | 0.162 | 4.49E-06 | 0.022546051 | Down |
| P17897 | Lysozyme C-1 OS=Mus musculus OX=10090 GN=Lyz1 PE=1 SV=1 | Lyz1 | 1.556 | 1.822 | 1.857 | 0.223 | 0.199 | 0.22 | 0.123 | 6.05E-06 | 0.029725631 | Down |
