# Supplementary material for: Glycometabolism change during Burkholderia pseudomallei infection in RAW264.7 cells by proteomic analysis
Source: Sci Rep. 2022 Jul 22;12:12560. doi: 10.1038/s41598-022-16716-z (PMC9307605; doi:10.1038/s41598-022-16716-z)
Supplement: Supplementary file 3 — Supplementary Table 2. [file 41598_2022_16716_MOESM3_ESM.docx]

Supplementary table 2: The top 20 significantly altered Biological Processes of up-regulated and down-regulated proteins in which p.adjust represented the Benjamini - Hochberg adjusted P value.

| **Cluster** | **ID** | **Description** | **p.adjust** |
| --- | --- | --- | --- |
| Down | GO:0022613 | ribonucleoprotein complex biogenesis | 2.9866E-48 |
| Down | GO:0042254 | ribosome biogenesis | 2.5685E-44 |
| Down | GO:0006364 | rRNA processing | 1.2361E-36 |
| Down | GO:0016072 | rRNA metabolic process | 8.1814E-36 |
| Down | GO:0006397 | mRNA processing | 6.2812E-35 |
| Down | GO:0008380 | RNA splicing | 1.2362E-34 |
| Down | GO:0034470 | ncRNA processing | 6.9935E-31 |
| Down | GO:0034660 | ncRNA metabolic process | 1.3399E-26 |
| Down | GO:0006403 | RNA localization | 5.9509E-26 |
| Down | GO:0050657 | nucleic acid transport | 5.16E-23 |
| Down | GO:0050658 | RNA transport | 5.16E-23 |
| Down | GO:0051236 | establishment of RNA localization | 8.8889E-23 |
| Down | GO:0000375 | RNA splicing, via transesterification reactions | 1.5159E-22 |
| Down | GO:0000377 | RNA splicing, via transesterification reactions with bulged adenosine as nucleophile | 1.5159E-22 |
| Down | GO:0000398 | mRNA splicing, via spliceosome | 1.5159E-22 |
| Down | GO:0015931 | nucleobase-containing compound transport | 1.767E-20 |
| Down | GO:0000819 | sister chromatid segregation | 4.1171E-20 |
| Down | GO:0071426 | ribonucleoprotein complex export from nucleus | 1.0739E-19 |
| Down | GO:0071166 | ribonucleoprotein complex localization | 1.448E-19 |
| Down | GO:0051028 | mRNA transport | 1.474E-19 |
| Up | GO:0009117 | nucleotide metabolic process | 1.9851E-22 |
| Up | GO:0006753 | nucleoside phosphate metabolic process | 4.8028E-22 |
| Up | GO:0006163 | purine nucleotide metabolic process | 1.2237E-19 |
| Up | GO:0006418 | tRNA aminoacylation for protein translation | 2.3805E-19 |
| Up | GO:0043039 | tRNA aminoacylation | 1.0724E-18 |
| Up | GO:0043038 | amino acid activation | 1.7175E-18 |
| Up | GO:0019693 | ribose phosphate metabolic process | 2.5034E-18 |
| Up | GO:0072521 | purine-containing compound metabolic process | 6.7745E-18 |
| Up | GO:0009165 | nucleotide biosynthetic process | 3.047E-17 |
| Up | GO:0009259 | ribonucleotide metabolic process | 4.072E-17 |
| Up | GO:1901293 | nucleoside phosphate biosynthetic process | 5.9414E-17 |
| Up | GO:0006520 | cellular amino acid metabolic process | 1.2789E-16 |
| Up | GO:0009150 | purine ribonucleotide metabolic process | 7.0799E-16 |
| Up | GO:0006399 | tRNA metabolic process | 1.6344E-15 |
| Up | GO:0090407 | organophosphate biosynthetic process | 3.0546E-15 |
| Up | GO:0009127 | purine nucleoside monophosphate biosynthetic process | 1.4748E-14 |
| Up | GO:0009168 | purine ribonucleoside monophosphate biosynthetic process | 1.4748E-14 |
| Up | GO:0009167 | purine ribonucleoside monophosphate metabolic process | 7.352E-14 |
| Up | GO:0009126 | purine nucleoside monophosphate metabolic process | 1.2045E-13 |
| Up | GO:0009156 | ribonucleoside monophosphate biosynthetic process | 2.0468E-13 |
