# Supplementary material for: Glycometabolism change during Burkholderia pseudomallei infection in RAW264.7 cells by proteomic analysis
Source: Sci Rep. 2022 Jul 22;12:12560. doi: 10.1038/s41598-022-16716-z (PMC9307605; doi:10.1038/s41598-022-16716-z)
Supplement: Supplementary file 4 — Supplementary Table 3. [file 41598_2022_16716_MOESM4_ESM.docx]

Supplementary talbe 3: The significantly enriched KEGG pathways in which NES and p.adjust represented normalized enrichment score and Benjamini - Hochberg adjusted P value, respectively.

| ID | Description | NES | p.adjust |
| --- | --- | --- | --- |
| mmu00970 | Aminoacyl-tRNA biosynthesis | 2.84182188 | 2.30833E-09 |
| mmu00230 | Purine metabolism | 2.83472698 | 2.30833E-09 |
| mmu03050 | Proteasome | 2.81239178 | 2.30833E-09 |
| mmu01230 | Biosynthesis of amino acids | 2.80200655 | 2.30833E-09 |
| mmu01200 | Carbon metabolism | 2.74162732 | 2.30833E-09 |
| mmu00520 | Amino sugar and nucleotide sugar metabolism | 2.63931491 | 2.07815E-08 |
| mmu05100 | Bacterial invasion of epithelial cells | 2.61632045 | 8.77456E-09 |
| mmu00030 | Pentose phosphate pathway | 2.52410732 | 1.87568E-07 |
| mmu00051 | Fructose and mannose metabolism | 2.51314517 | 4.63049E-07 |
| mmu01240 | Biosynthesis of cofactors | 2.49773284 | 5.53979E-09 |
| mmu00270 | Cysteine and methionine metabolism | 2.4807003 | 1.20956E-06 |
| mmu00010 | Glycolysis / Gluconeogenesis | 2.38800218 | 3.62537E-06 |
| mmu00052 | Galactose metabolism | 2.34510184 | 5.96727E-06 |
| mmu00240 | Pyrimidine metabolism | 2.34022143 | 1.63166E-05 |
| mmu00480 | Glutathione metabolism | 2.27397439 | 4.11802E-05 |
| mmu04722 | Neurotrophin signaling pathway | 2.24856586 | 1.63166E-05 |
| mmu04150 | mTOR signaling pathway | 2.237593 | 1.63166E-05 |
| mmu04922 | Glucagon signaling pathway | 2.13846068 | 0.000133797 |
| mmu04136 | Autophagy - other | 2.09621961 | 0.000362579 |
| mmu04660 | T cell receptor signaling pathway | 2.06161659 | 0.000707719 |
| mmu00983 | Drug metabolism - other enzymes | 2.03599767 | 0.001000803 |
| mmu00040 | Pentose and glucuronate interconversions | 1.97363413 | 0.001587763 |
| mmu00500 | Starch and sucrose metabolism | 1.89345165 | 0.004452653 |
| mmu03060 | Protein export | -1.87903088 | 0.011112792 |
| mmu03020 | RNA polymerase | -1.88758103 | 0.010989861 |
| mmu00100 | Steroid biosynthesis | -2.2430841 | 0.000204999 |
